# Checklist and provisional atlas of singing cicadas (Hemiptera: Cicadidae) of Bulgaria, based on bioacoustics

**DOI:** 10.3897/BDJ.8.e54424

**Published:** 2020-10-09

**Authors:** Tomi Trilar, Ilia Gjonov, Matija Gogala

**Affiliations:** 1 Slovenian Museum of Natural History, Ljubljana, Slovenia Slovenian Museum of Natural History Ljubljana Slovenia; 2 Sofia University, Faculty of Biology, Department of Zoology and Anthropology, Sofia, Bulgaria Sofia University, Faculty of Biology, Department of Zoology and Anthropology Sofia Bulgaria; 3 Slovenian Academy of Sciences and Arts, Ljubljana, Slovenia Slovenian Academy of Sciences and Arts Ljubljana Slovenia

**Keywords:** Hemiptera, Cicadoidea, singing cicadas, fauna, bioacoustics, distribution, Bulgaria

## Abstract

**Background:**

The singing cicadas (Hemiptera: Cicadidae) of Bulgaria were poorly known. There are published records for 14 species: *Cicada orni*, *Lyristes plebejus*, *Cicadatra atra*, *Cicadatra hyalina*, *Cicadatra persica*, *Cicadetta montana*, *Cicadetta mediterranea*, *Dimissalna dimissa*, *Oligoglena tibialis*, *Tympanistalna gastrica*, *Pagiphora annulata*, *Saticula coriaria*, *Tibicina haematodes* and *Tibicina steveni*.

**New information:**

Two species from this list were doubtful in the beginning of our study, since *Tympanistalna gastrica* is distributed in central and southern Portugal and *Saticula coriaria* is a north African species.

We checked three major institutional collections housed in Sofia, Bulgaria: the National Museum of Natural History (SOFM), the Institute of Zoology (ZISB) and the Biology Faculty of Sofia University "St. Kliment Ohridski" (BFUS). We confirmed 11 of the species mentioned in literature, except *Cicadetta mediterranea* and found two additional species: *Cicadatra platyptera* and *Cicadetta macedonica* (the specimens in BFUS were bioacoustically confirmed).

Based on this knowledge, we further investigated the singing cicadas of Bulgaria with the use of morphological and bioacoustic methods in the years 2008, 2009, 2010, 2012, 2016, 2018 and 2019. We were not able to confirm the presence of *Cicadatra persica* and *Cicadetta mediterranea*, but found three additional species: *Cicadetta brevipennis* s. lat., *Cicadetta cantilatrix* and *Tettigettula pygmea*. Using the bioacoustic methods, we also detected unknown singing patterns, which could belong to three or four additional taxa, which need to be described.

The Bulgarian fauna of singing cicadas at the moment consists of 16 confirmed and 3-4 potential species.

## Introduction

The singing cicadas (Hemiptera: Cicadidae) of Bulgaria were poorly known and have received little attention until recently. There are published records for 14 species (Dlabola 1955, Arabadzhiev 1963, Dirimanow and Harisanow 1965, Janković 1971, Bairyamova 1976, Bairamova 1978, Bajrjamova 1990, Bairyamova 1992, Gogala et al. 2005, Háva 2016, Holzinger et al. 2003, Lodos and Kalkandelen 1981, Nast 1972, Nast 1987, Nedyalkov 1908, Pelov 1968, Quartau and Fonseca 1988, Sander 1985, Schedl 1986, Yoakimov 1909): *Cicada orni* Linnaeus, 1758, *Lyristes plebejus* (Scopoli, 1763), *Cicadatra atra* (Olivier, 1790), *Cicadatra hyalina* (Fabricius, 1798), *Cicadatra persica* Kirkaldy, 1909, *Cicadetta montana* s. lat. (Scopoli, 1772), *Cicadetta mediterranea* Fieber, 1876, *Dimissalna dimissa* (Hagen, 1856), *Oligoglena tibialis* (Panzer, 1798), *Tympanistalna gastrica* (Stål, 1854), *Pagiphora annulata* (Brullé, 1832), *Saticula coriaria* Stål, 1866, *Tibicina haematodes* (Scopoli, 1763) and *Tibicina steveni* (Krynicki, 1837).

The present paper provides an overview of the literature, data from the major collections housed in Bulgaria and new faunistic data collected mainly with bioacoustic methods, which significantly extend the earlier knowledge about the fauna of singing cicadas in this part of Europe.

## Materials and methods

Between 2008 and 2019, we investigated the singing cicadas (Hemiptera: Cicadidae) of Bulgaria with morphological and bioacoustic methods. In the periods from 22nd to 27th July 2008, from 15th to 24th June 2009, from 23rd to 30th July 2010, from 27th June to 7th July 2012, from 18th to 28th June 2016, from 29th June to 11th July 2018 and from 26th June to 9th July 2019 we carried out the joint fieldwork (in 2010 and 2012, the first author could not participate in the fieldwork).

For the sound recordings, we used microphones which are sensitive in the sonic range (Telinga Pro 6 stereo and Telinga Pro Science - parabola diameter 57 cm, frequency response 40-20,000 Hz) and in the ultrasonic range (ultrasonic detector Pettersson D-200 (frequency range 10-120 kHz) with microphones mounted in front of a Telinga reflector (57 cm diameter), Wildtronics Pro Mini Parabolic Dish (29 cm diameter), smaller metal reflector (15 cm diameter) and self made parabolas (diameters 12 cm, 20 cm and 40 cm) in combination with the Solid State recorders Marantz PMD660, PMD661 and PMD670, Tascam DR-600mkII, Zoom H2 and H4. For sound analysis, we used Raven 1.3 to 1.5 (Cornell Lab of Ornithology), Amadeus Pro 2.4.5. (HairerSoft), Wave Pad (NHC Software) and Seewave package (Sueur et al. 2008) as a part of R statistics software (R Development Core Team 2008). With these methods, we were able to obtain much more representative data on the presence and distribution of singing cicadas.

We first localised the cicadas acoustically, recorded them and then, if possible, collected them with an entomological net. Morphological studies were carried out on dry prepared specimens. Higher classification and morphological terminology are according to Marshall et al. (2018).

The specimens collected during the joint fieldwork are preserved in the collections of the Biology Faculty, Sofia University "St. Kliment Ohridski" (BFUS) in Sofia, Bulgaria and the Slovenian Museum of Natural History (PMSL) in Ljubljana, Slovenia. The audio recordings are stored at the Slovenian Wildlife Sound Archive of Slovenian Museum of Natural History (PMSL) in Ljubljana, Slovenia and at the file-hosting service of the Department of Zoology and Anthropology, Biology Faculty, Sofia University "St. Kliment Ohridski" (BFUS) in Sofia, Bulgaria. Selected sound samples are also available on the web pages *Songs of the European singing cicadas* (Gogala 2020).

The following information is given for each locality: metadata of the locality (province and municipality), name of the locality, coordinates, altitude (a.s.l.), date, type of data (song recorded, species recorded in the background, song heard, photographed or collected), number of specimens, if collected, repository or source and collectors of the data. Geographic coordinates are given in decimal degrees (datum WGS84). The number of decimal places depends on the accuracy of the data.

**Figure 1.**
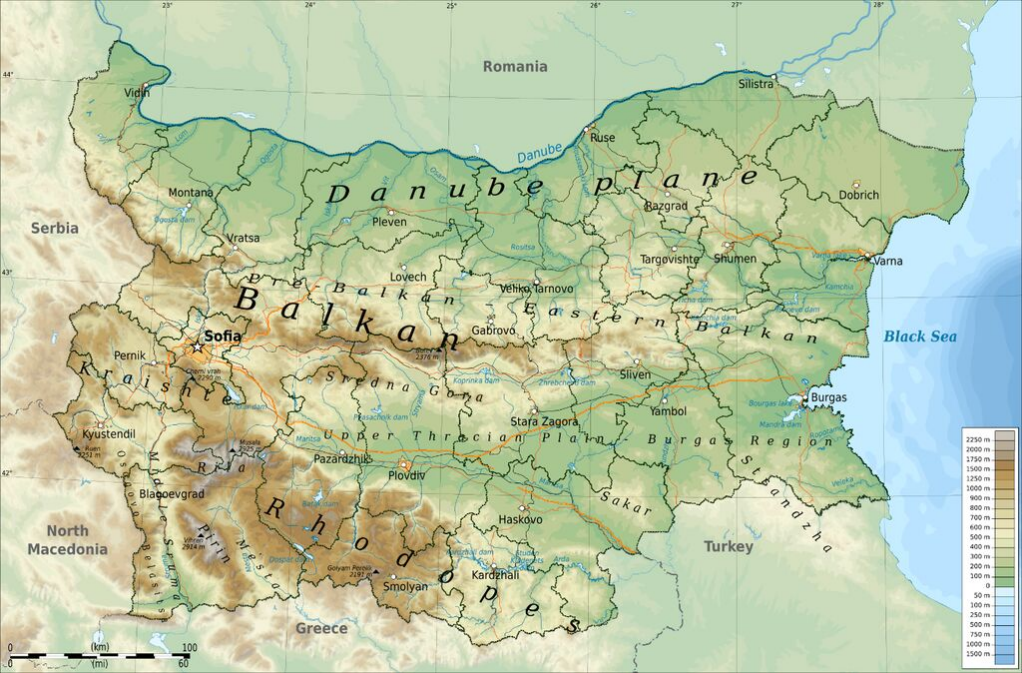
**Geographic map of Bulgaria** with the main geographical units and marked borders of the provinces and capitals. Source of the map Ikonact (2013).

To describe the distribution of the singing cicadas in Bulgaria, we used the geographical subdivision of Bulgaria (Fig. 1) (Kopralev et al. 2002). Distribution maps were created with the GPS Visualizer (Schneider 2020), spectrograms and oscillograms with the Seewave package (Sueur et al. 2008) as a part of R statistics software (R Development Core Team 2008).

In addition to the fieldwork, records of the cicadas from the public collections of the National Museum of Natural History (SOFM), the Institute of Zoology (ZISB) and the Biology Faculty of Sofia University "St. Kliment Ohridski" (BFUS), all located in Sofia, Bulgaria, were checked for usable data.

We also tried to check all potentially-relevant regional, national and international zoological journals or series for references of cicadas from Bulgaria.

ACRONYMS

Collections (see http://hbs.bishopmuseum.org/codens/codens-inst.html)

BFUS – Biology Faculty, Sofia University "St. Kliment Ohridski", Sofia, Bulgaria

MZPW – Museum and Institute of Zoology, Polish Academy of Science, Warszawa, Poland

PMSL – Slovenian Museum of Natural History, Ljubljana, Slovenia

SOFM – National Museum of Natural History, Sofia, Bulgaria

ZISB – Institute of Zoology, Sofia, Bulgaria

## Data resources

### Overview on the data set

Some material that is in the public collections of the National Museum of Natural History (SOFM), the Institute of Zoology (ZISB) and the Biology Faculty of Sofia University "St. Kliment Ohridski" (BFUS), located in Sofia, have been collected by different collectors. We have examined, reviewed, evaluated and re-determined all the material in the collections. In addition to 11 species already mentioned in literature, with the exception of *Cicadetta mediterranea*, *Tympanistalna gastrica* and *Saticula coriaria*, we found two additional species: *Cicadatra platyptera* Fieber, 1876 and *Cicadetta macedonica* Schedl, 1999. In collections, we also found *Pagiphora annulata*, which was falsely identified as *Tympanistalna gastrica*.

**Figure 2.**
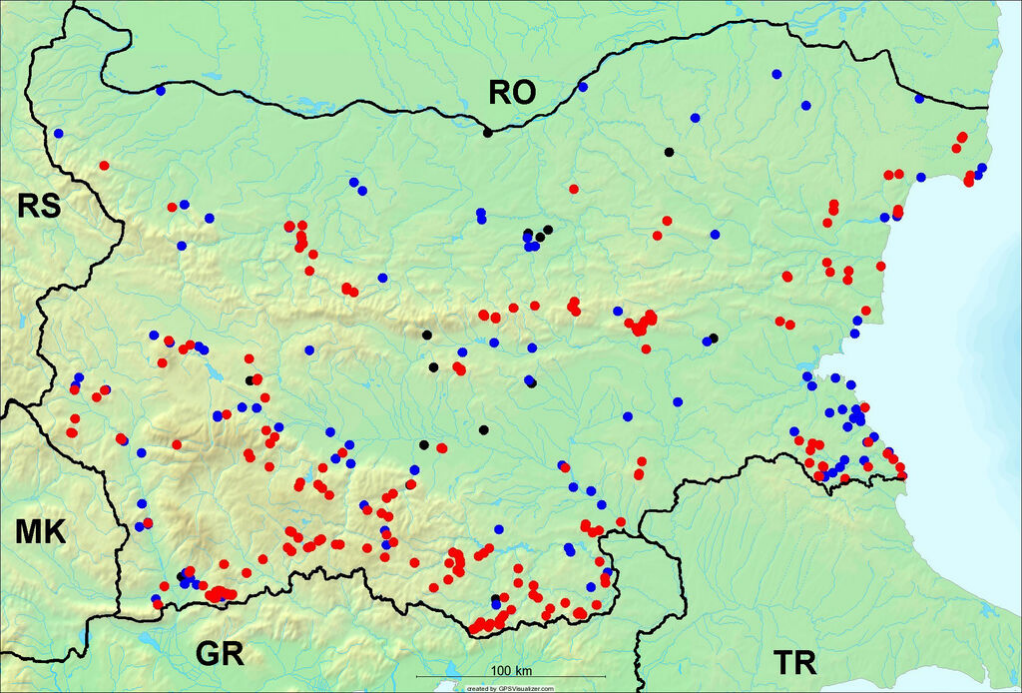
**Map with localities with cicada records in Bulgaria.** Black - literature data, blue - data from collections, red - bioacoustic data collected in this survey. GR - Greece, MK - North Macedonia, RO - Romania, RS - Serbia, TR - Turkey.

**Figure 3.**
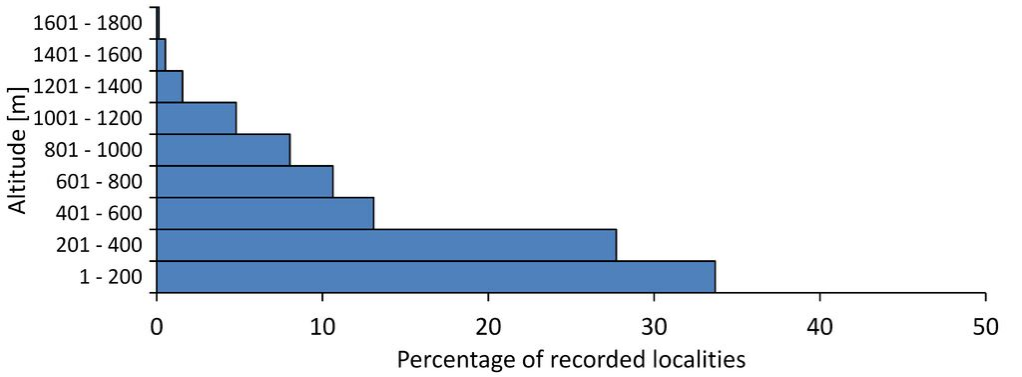
**Altitudinal distribution of all data.** The percentage of the recorded localities is displayed in 200-metre altitude zones.

In the joint fieldwork, we mapped singing cicadas at 250 localities using morphological and bioacoustic methods (Figs 2, 3). At 23 localities, singing cicadas were not present for various reasons, but mainly due to unsuitable weather. From the remaining 226 localities, we collected 549 faunistic data (song recorded, species recorded in the background, song heard, photographed or collected).

Faunistic data for single species are presented below.

## Checklists

### Annotated checklist of the cicadas of Bulgaria

#### Cicadinae

Latreille, 1802

F8CEA849-783D-5EDB-9D94-34327BE3CA19

#### Cicadini

Latreille, 1802

2AEEACB0-7F87-55B5-9DDC-2FB9FCA55F9A

#### Cicada orni

Linnaeus, 1758

29E0B5CC-DA25-5A97-804D-5F3046BC6F31

##### Distribution

**General distribution: Southern Europe**: Albania, Bulgaria, Croatia, France (including Corsica), Greece, Italy (including Sardinia and Sicily), Montenegro (Trilar & Gogala, unpublished data), North Macedonia, Portugal, Romania, Serbia, Slovenia, Spain (including Balearic Islands); **Central Europe**: Austria, Hungary, Germany, Slovakia, Switzerland; **Eastern Europe**: Russia (South European Russia), Ukraine; **Middle East**: Cyprus, Iran, Israel, Jordan, Lebanon, Turkey; **Transcaucasia**: Armenia, Azerbaijan, Georgia, Russia (Chechnya); **Northern Africa**: Algeria, Egypt, Tunisia; **Central Asia**: Turkmenistan (all data except for the countries with the citation in brackets are summarised by Duffels and van der Laan (1985) and Sanborn (2014).

*Cicada orni* is in older literature cited also for the Greek island Crete (Nast 1972, Quartau and Fonseca 1988), from where *Cicada cretensis* Quartau & Simões 2005 was later described (Quartau and Simões 2005, Pinto-Juma et al. 2009, Simões and Quartau 2009, Trilar and Gogala 2010).

*Cicada orni* is on the eastern side of Rechinger's line in the Aegean Sea replaced by *Cicada mordoganensis* Boulard, 1979 (Simões and Quartau 2013, Gogala and Trilar 2014), which is bioacoustically proven in the east Aegean Islands: Chios, Ikaria, Samos, Kos and Rhodes (Simões and Quartau 2008, Pinto-Juma et al. 2009, Gogala and Trilar 2014), as well as Kalymnos (Trilar & Gogala, unpublished data) and further east in south-western Turkey along the sea coast (at least in the Provinces of Izmir, Aydin, Muǧla and Antalya) (Boulard 1979, Lodos and Kalkandelen 1981, Boulard 1995, Kemal and Koçak 2010, Zeybekoğlu et al. 2011).

**Distribution in Bulgaria**: *Cicada orni* is a very abundant and widespread species of singing cicadas distributed in Bulgaria. Data are available for 74 localities (Fig. 5). In literature, we found data for Bulgaria in Nedyalkov (1908), Yoakimov (1909) and Sander (1985). Bairyamova (1992) lists the species for Rhodope Mts. The species is also listed in overviews by Nast (1972), Nast (1987), but without the exact localities for Bulgaria.

In Bulgaria, *Cicada orni* is distributed all over the country: in eastern Danubian Plane, northern lowest hills of the Pre-Balkan, southern Lower Mountain Pre-Balkan, northern Balkan Mts., southern Balkan Mts., Eastern Sub-Balkan valleys, Kraishte-Ichtiman, Kyustendil-Blagoevgrad Middle Struma valley, Sandanski-Petrich Middle Struma valley, Pirin Mt., Prespa-Chernatitsa Western Rhodope Mts., Upper Thracian Plain, Tundzha-Burgas Valley, Eastern Rhodope Mts. and Strandzha Mt. (Fig. 5).

In this survey the majority of the populations were found between sea level and 800 metres (95% of the population) (Fig. 6). The highest points where we recorded the species were in the Eastern Rhodopes at Hvoynova Polyana at Gyumyurdzhiyski Snezhnik (917 m a.s.l.) and at the slope above the Pass Makaza (909 m a.s.l.).

##### Notes

**Acoustic behaviour:** The song was described by Popov (1975), Joermann and Schneider (1987) and Boulard (1995). The variability as a function of location and environmental conditions was described in later works (Claridge et al. 1979, Quartau et al. 1999, Quartau et al. 2000, Simões et al. 2000, Pinto-Juma et al. 2005, Seabra et al. 2008, Mehdipour et al. 2016). The sound reception of this species was recently investigated by Sueur et al. (2010).

The males of *Cicada orni* can sing for hours without interruption from a single spot, sometimes chorusing with other males. The calling song is a pattern of regular repetitions (5-8 times per second) of echemes and interecheme intervals, where the echemes are composed of a variable number of groups of pulses (Fig. 7). The duration of the echemes is 84-116 ms; the interecheme interval 84-93 ms, the frequency range is 4.45 ± 0.2 kHz and the peak frequency 4.24 kHz (Popov 1975, Joermann and Schneider 1987, Fonseca 1991, Boulard 1995, Claridge et al. 1979, Quartau et al. 1999, Quartau et al. 2000, Simões et al. 2000, Gogala 2002, Pinto-Juma et al. 2005, Quartau and Simões 2005, Seabra et al. 2006, Simões et al. 2006, Sueur et al. 2010, Zeybekoğlu et al. 2011, Mehdipour et al. 2016).

Selected sound samples of *Cicada orni* are available on the web pages *Songs of the European singing cicadas* (Gogala 2020).

**Materials**: Suppl. material 1

##### Diagnosis

**Figure 4.**
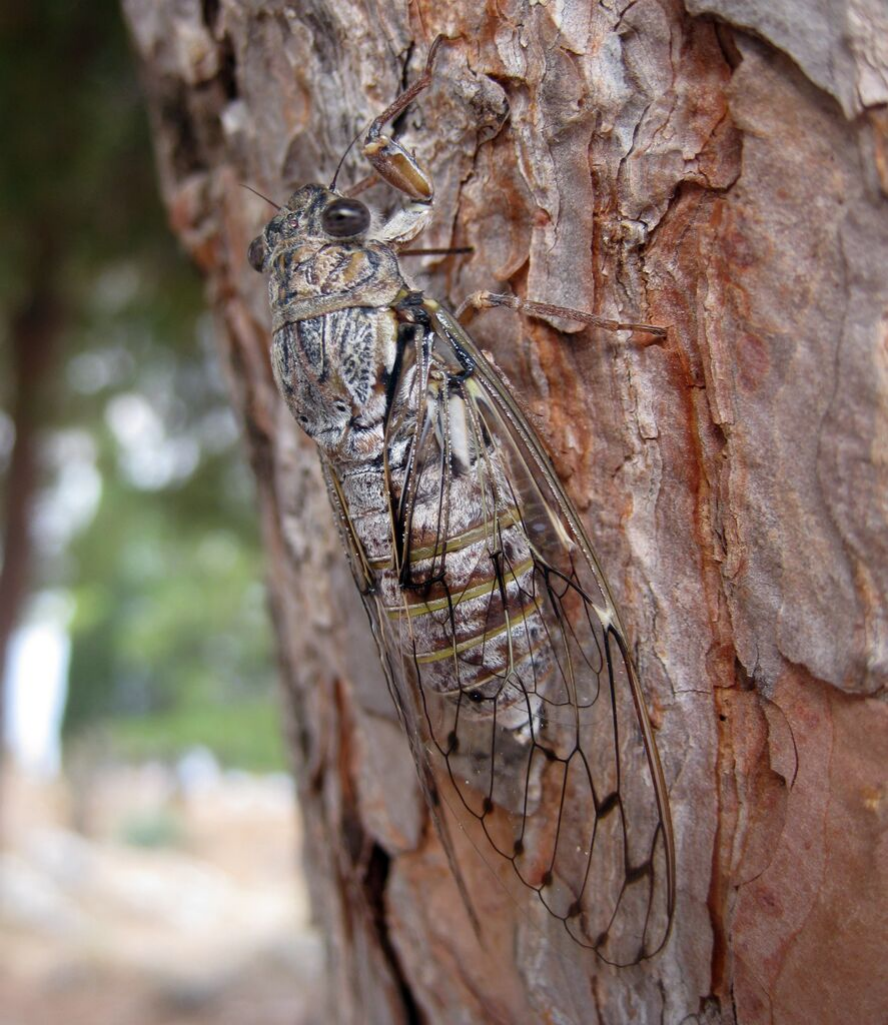
***Cicada orni* male.**

*Cicada orni* (Fig. 4) is one of the most abundant and common cicadas throughout the Mediterranean area. The singing males are often singing in chorus and are commonly observed in closed high shrublands and woodlands, as well as in olive trees, fruit trees, vineyards and gardens and also on fences and poles (Popov 1975, Patterson et al. 1997, Puissant and Sueur 2001, Sueur et al. 2004, Pinto-Juma et al. 2005, Hertach and Nagel 2013).

#### Cryptotympanini

Handlirsch, 1925

21EF8830-4850-5CA5-9DDE-734209A55AB4

#### Lyristes plebejus

(Scopoli, 1763)

EF271103-DC22-55E3-B4DE-F1AEBC452E4A

##### Distribution

**General distribution: Southern Europe**: Albania, Bulgaria, Croatia (Novak and Wagner 1962, Schedl 1986, Joermann and Schneider 1987), France, Greece, Italy (including Sicily), Montenegro (Trilar & Gogala, unpublished data), North Macedonia, Portugal, Romania, Serbia (Petrik 1958, Janković 1975), Slovenia, Spain; **Central Europe**: Austria, Hungary, Poland, Slovakia, Switzerland; **Eastern Europe**: Russia (South European Russia), Ukraine; **Middle East**: Iran; **Transcaucasia**: Armenia, Azerbaijan, Georgia (all data except for the countries with the citation in brackets are summarised by Duffels and van der Laan 1985 and Sanborn 2014).

There is a doubtful citation of *Lyristes plebejus* for Germany (Nast 1972, Wolf 1974, Nast 1987, Lodos and Kalkandelen 1981, Quartau and Fonseca 1988), as it is not listed in the latest overview of fauna (Nickel et al. 2016). There are also doubtful records for Corsica (Servadei 1967, Nast 1972, Boulard 1992b, Guglielmino et al. 2000) and Sardinia (Servadei 1967, Nast 1972, Boulard 1992b, Quartau and Fonseca 1988, Guglielmino et al. 2000), as *Lyristes plebejus* was never found in recent fieldwork with bioacoustic methods on these Islands (Puissant and Sueur 2001; Thomas Hertach, personal communication).

*Lyristes plebejus* is on the eastern side of Rechinger's line in the Aegean Sea replaced by *Lyristes gemellus* Boulard, 1988 (Simões and Quartau 2013, Gogala and Trilar 2014), which is bioacoustically proven on the east Aegean Islands: Lesbos, Chios, Ikaria, Samos and Rhodes (Simões and Quartau 2013, Gogala and Trilar 2014), as well as Kalymnos and Kos (Trilar & Gogala, unpublished data) and further east in Turkey (Provinces of Aydin, Antalya and Mersin) (Trilar & Gogala, unpublished data) and Cyprus (Simões and Quartau 2013). *Lyristes plebejus* is also cited in literature for Israel (Schedl 1999) and Syria (Lodos and Kalkandelen 1981), but in our opinion, *Lyristes gemellus should also be distributed here*, but this assumption needs to be bioacoustically investigated.

**Distribution in Bulgaria**: In Bulgaria, data are available for 76 localities (Fig. 9). In literature, we found the data for Bulgaria in Yoakimov (1909), Sander (1985) and Háva 2016. Bairyamova (1992) lists the species for Rhodope Mts. and Nedyalkov (1908) for northern and southern Bulgaria, with the remark that it does not occur above the altitude limit of oak distribution. Arabadzhiev (1963) considers the species as a pest. The species is also listed in overviews by Nast (1972), Nast (1987), but without exact location information for Bulgaria.

In Bulgaria, *Lyristes plebejus* is distributed all over the country with the known data in eastern Danubian Plane, northern lowest hills of the Pre-Balkan, eastern Sub-Balkan valleys, Ograzhden-Vlachina Mts., Sandanski-Petrich Middle Struma valley, Pirin Mt., Dabrash-Batak western Rhodope Mts., Prespa-Chernatitsa western Rhodope Mts., Upper Thracian Plain, Tundzha-Burgas Valley, eastern Rhodope Mts., Haskovo Hills Land and Strandzha Mt. (Fig. 9).

In this survey, the majority of the population was found between sea level and 800 m (93% of the population) (Fig. 10). At the highest altitude, the male specimen from the SOFM collection was recorded, which was found in Chepelare by Vl. Nonev (1105 m a.s.l.).

##### Notes

**Acoustic behaviour**: The song was described by Popov (1969), Popov (1975), Joermann and Schneider (1987), Claridge et al. (1979), Boulard (1995), Gogala (2002), Sueur et al. (2004), Puissant (2012) and Mehdipour et al. 2015. The sound reception of this species has recently been investigated by Sueur et al. (2010).

The song of *Lyristes plebejus* has a very complex structure (Fig. 11). It is a continuous repetition of phrases (duration 7-30 s) without a pause lasting for many minutes. Each phrase can be divided into three parts. The first two parts consist of a fast sequence of short echemes (SE) and the third of a steady buzz (Gogala 2002, Joermann and Schneider 1987). In the first part, which extends over half of the phrase, 30-240 SE are repeated without pause. This is followed by the transition in the second part, which consists of 25-80 SE, separated by intervals of 15-30 ms. The echeme period (85 ms) is constant over almost the entire first two phrases. Finally, the transition occurs in the third part (duration 1.5-3.0 s) which is characterised by the loss of the echeme structure and has become a steady buzz (Joermann and Schneider 1987). In the first and second part of the phrase, there is an amplitude modulation of the echemes, which is higher in the first part than in the second part (Joermann and Schneider 1987). The bandwidth measured 10 dB below the maximum is fairly uniform and ranges from 4.4 kHz to 8.34 kHz, but there is some variation in peak frequency between the phrases (Joermann and Schneider 1987).

Selected sound samples of *Lyristes plebejus* can be found on the web pages *Songs of the European singing cicadas* (Gogala 2020).

**Materials**: Suppl. material 2

##### Diagnosis

*Lyristes plebejus* (Fig. 8) is the second largest European cicada after the south-eastern European species *Lyristes gemellus* Boulard, 1988 and has a very loud and distinct song (Hertach and Nagel 2013). The species occurs mainly in closed high shrubland and woodland on various plants, such as olive trees, pines, oaks, as well as on fruit trees (Sueur et al. 2004, Drosopoulos et al. 2005, Simões and Quartau 2013). In some areas, they can be observed in large numbers singing together in a chorus (Gogala 2002).

#### Cicadettinae

A2C55679-AB3A-5BD4-BDC1-43C0DECCBA8F

#### Cicadatrini

Distant, 1905

FCB88D84-49F3-5157-B294-317C583218AA

#### Cicadatra atra

(Olivier, 1790)

FE2EACF2-C52A-5A73-A11F-CB574C1C20D3

##### Distribution

**General distribution: Southern Europe**: Albania, Bulgaria, Croatia, France, Greece, Italy (including Sicily), Montenegro (Trilar & Gogala, unpublished data), North Macedonia, Romania, Serbia, Slovenia, Spain; **Eastern Europe**: Russia (South European Russia), Ukraine; **Middle East**: Cyprus, Iran, Israel, Lebanon, Palestine, Syria, Turkey; **Transcaucasia**: Armenia, Azerbaijan, Georgia, Russia (Chechnya) (all data except for the countries with the citation in brackets are summarised by Duffels and van der Laan (1985), Quartau and Fonseca (1988) and Sanborn (2014).

There are dubious citations of *Cicadatra atra* for Czech Republic (Nast 1972, Schedl 2000, Holzinger et al. 2003), Germany (Schedl 1986) and Switzerland (Nast 1972, Schedl 1986, Nast 1987, Lodos and Kalkandelen 1981, Schedl 2000, Günthart and Mühlenthaler 2002), as it is not listed in the new faunistic papers for these countries (Malenovský and Lauterer 2017, Nickel et al. 2016, Hertach and Nagel 2013). The same is probably true for Hungary (Schedl 1986). Doubtful are also quotations for Corsica (Servadei 1967, Nast 1972, Boulard 1992b) and Sardinia (Servadei 1967, Nast 1972, Quartau and Fonseca 1988), since *Cicadatra atra* was never found in recent fieldwork with bioacoustic methods on these islands (Thomas Hertach, personal communication).

**Distribution in Bulgaria**: *Cicadatra atra* is abundant throughout Bulgaria and data are known for 79 localities (Fig. 13). In literature, we found data for Bulgaria in Nedyalkov (1908), Yoakimov (1909), Dirimanow and Harisanow (1965) and Sander (1985). It is also cited in Háva (2016), but misidentified as *Cicadatra persica*. *Cicadatra atra* is also mentioned in the overviews by Nast (1972), Nast (1987) and Lodos and Kalkandelen (1981), but without precise location information for Bulgaria.

In Bulgaria, it is distributed all over the country with the known data in western Danubian Plane, eastern Danubian Plane, northern lowest hills of the Pre-Balkan, Kraishte-Ichtiman, Kyustendil-Blagoevgrad Middle Struma valley, Sandanski-Petrich Middle Struma valley, Rila Mt., Pirin Mt., Dabrash-Batak western Rhodope Mts., Prespa-Chernatitsa western Rhodope Mts., Upper Thracian Plain, Tundzha-Burgas Valley, eastern Rhodope Mts., Haskovo Hills Land, Sakar Mt. and Strandzha Mt. (Fig. 13).

In this study, the majority of the population was found between sea level and 600 m a.s.l. (86% of the population) (Fig. 14), but also include populations up to 1570 m a.s.l. where two female specimens kept in the SOFM collection were collected by an unknown collector in Pamporovo.

##### Notes

**Acoustic behaviour**: The song was described by Popov (1975), Boulard (1992b) and Boulard (1995), while the mechanism of wing clicking was described by Gogala and Trilar (2003) and the sound reception by Sueur et al. (2010).

Four types of songs are registered in *Cicadatra atra*: continuous calling song, intermittent calling song, courtship song and alarm song (rivalry song or distress call) (Popov 1975, Boulard 1992b).

*Continuous calling song* consists of a sound with a constant amplitude, which is up to several minutes long and resembles a smooth high-frequency buzz (Fig. 15), which is randomly interrupted by short pauses (Popov 1975, Boulard 1992b, Boulard 1995). Due to the alternating tymbals, the pulse structure is poorly defined (Popov 1975). The frequency range is between 8.0 and 16.0 kHz and the main peak frequency is about 10.0 kHz and the second is about 12.2 kHz (Boulard 1995).

*Intermittent calling song* consists of short echemes (duration 95-129 ms) and the interecheme periods of approximately equal length (Fig. 15). During the short echemes, the intensity of the song rises smoothly and ends steeply at the end. Each echeme consists of successive sequences of pulses whose frequency increases by 1/3 towards the end of the echeme. The frequency characteristics are almost the same as in the continuous calling song (Popov 1975).

*Courtship song* (Fig. 16) have the same structure, temporal parameters and frequency characteristics of short echemes as intermittent calling song with additional wing clicks in the middle of the interecheme interval (Boulard 1992b, Gogala 2002, Gogala et al. 2005). The clicks always occur in connection with the opening and closing of the forewings and the hind wings also move a little bit. The click appears in the phase of closing the wings, but in the phase of opening the wings, the soft preclicks of the wings are clearly visible, which occurred 8-9 ms before the main click. The inner edges of the tegmina remain attached to the mesonotum throughout the whole cycle and the forewings open to an almost horizontal plane with wing tips raised above the abdomen (Gogala and Trilar 2003).

Boulard (1992b) also described the second phase of courtship behaviour in which male and female stand side by side and the male produces a chirping song shortly before copulation begins.

Selected sound samples of *Cicadatra atra* are available on the web pages *Songs of the European singing cicadas* (Gogala 2020).

**Materials**: Suppl. material 3

##### Diagnosis

*Cicadatra atra* (Fig. 12) is a highly polyphagous species (Boulard 1992b, Holzinger et al. 2003), which is environmentally undemanding. It is found in very different habitats with trees, shrubs and herbs, in the garrigue, in orchards, more rarely in olive groves, sometimes in vineyards (Boulard 1992b). In the Black Sea area, it is reported to occur exclusively in grass (Popov 1975). The males of *Cicadatra atra* never sing very high in the trees, usually less than 3 m above the ground (Boulard 1992b).

#### Cicadatra hyalina

(Fabricius, 1798)

FFE502BC-32CF-58BE-B471-6C76813F7687

##### Distribution

**General distribution: Southern Europe**: Greece, North Macedonia, Romania; **Eastern Europe**: Russia (South European Russia), Ukraine; **Middle East**: Iran, Israel, Jordan, Palestine, Syria, Turkey; **Transcaucasia**: Armenia, Azerbaijan, Georgia; **Central Asia**: Turkmenistan (summarised by Duffels and van der Laan 1985 and Sanborn 2014).

**Distribution in Bulgaria**: In Bulgaria, data are known for four localities from eastern Danubian Plane, Sandanski-Petrich Middle Struma valley and Upper Thracian Plain (Fig. 18). In literature, we found the data in Nedyalkov (1908), who cites *Cicadatra hyalina* for Sadovo and Pazardzhik. One female specimen is stored in the BFUS collection and was collected by the third author in Harsovo. During this study, it was recorded and collected at Kaliakra Cape in steppe habitats close to the Black Sea coast (Fig. 18). All these sites are located between 60 and 260 m a.s.l. (Fig. 19).

##### Notes

**Acoustic behaviour**: Four types of songs are registered in *Cicadatra hyalina*: continuous calling song, intermittent calling song, courtship song and alarm song (rivalry song or distress call) (Popov 1975, Boulard 1995).

*Continuous calling song* (Fig. 20) consists of a sound with constant amplitude, which is up to several minutes long and resembles a high-frequency buzz (Popov 1975) with the frequency range 8.3 to 12.5 kHz and the peak frequency around 11.0 kHz (Boulard 1995, Popov et al. 1991).

The *intermittent calling song* resembles a rumbling noise and consists of echemes of 0.8-1.6 s in length and the interecheme periods of approximately the same length (Fig. 21). Echemes consist of successive pulse sequences with frequencies of 47-60 Hz (Fig. 20B). Exceptions are the first few pulses of the echeme, which follow at intervals 1.5-2.0 times longer than the next (Popov 1975). The frequency range of short echemes has a relatively low and wide band between 3 and 11 kHz (Popov 1975, Boulard 1995), with two peak frequencies at 4.5 kHz and 10.5 kHz (Popov et al. 1991).

The *courtship song* is a sequence of pulses that is regularly repeated at a frequency of 54-59 Hz. It has a similar rumbling sound as the intermittent calling song with the same spectral characteristics, but it lacks the segmentation in echemes and is much quieter than the intermittent calling song (Popov 1975).

Selected sound samples of *Cicadatra hyalina* are available on the web pages *Songs of the European singing cicadas* (Gogala 2020).

**Materials**: Suppl. material 4

##### Diagnosis

*Cicadatra hyalina* (Fig. 17) lives in hot and dry open grasslands (Gogala et al. 2005), where it inhabits grass or low shrubs at a height of up to 1 m (Popov 1975). Sometimes, it is even found on rocky semi-desert grasslands with very little vegetation.

#### Cicadatra persica

(Kirkaldy, 1909)

742A0C50-C87E-5CB3-B2E1-9E5FF1948E7C

##### Distribution

**General distribution: Southern Europe**: Bulgaria (this study), North Macedonia; **Eastern Europe**: Russia (south European Russia); **Middle East**: Iran, Israel, Syria, Turkey; **Transcaucasia**: Armenia, Azerbaijan, Georgia; **Central Asia**: Pakistan (summarised by Duffels and van der Laan 1985 and Sanborn 2014).

According to Kudryasheva (1979) the western limit of distribution of *Cicadatra persica* in the Mediterranean reaches as far as Sicily, but has not been confirmed by recent fieldwork with bioacoustic methods (Thomas Hertach, personal communication).

**Distribution in Bulgaria**: Two male and one female specimen of *Cicadatra persica* from Sliven are kept in the collections of SOFM and MZPW (Figs 23, 24). These data have never been published before.

*Cicadatra persica* is also mentioned for Bulgaria in literature by Háva (2016) from Sozopol on the Black Sea coast, but in this study, we find that the specimen was misidentified with an unusually coloured *Cicadatra atra* because of the large differences in body size.

Using bioacoustic methods, we searched in Sliven and its surroundings under ideal weather conditions for singing cicadas and in suitable habitat (based on our own field experiences from North Macedonia (Gogala and Trilar 1998, Gogala and Trilar 2003, Gogala et al. 2005)), but could not detect the presence of *Cicadatra persica*.

##### Notes

**Acoustic behaviour**: The song with intensive wing clicking was described by Gogala and Trilar (1998). Some years later, Gogala and Trilar (2003) also described the mechanism of wing clicking.

Like many other *Cicadatra* species, *Cicadatra persica* also has two types of songs: continuous calling song and courtship song with wing clicks. It is obvious that many species of this genus combine the normal tymbal sounds with clicks produced by wing beats against the body or substrate during their courtship song (Gogala and Trilar 1998).

The phrases of a *continuous calling song* can last for many minutes without interruption (Fig. 25). They begin without a distinct pattern of amplitude modulation and sometimes end with one or a few separate, irregular echemes. The frequency range is between 5.8 and 12.4 kHz (at -20 dB level) with maximum amplitude close to 8.4 kHz. In this wide frequency band, there are many small frequency peaks, 670-1300 Hz apart due to the ultrastructure of this song, which consists of pulses (tymbal rib clicks) with a repetition period of about 0.8 to 1.5 ms (repetition frequency 670 - 1250 Hz) (Gogala and Trilar 1998).

*Courtship song* (Fig. 26) consists of phrases (duration 5-7 s, average 6 ± 1.3 s) with a long series (N = 59 ± 14) of rapidly repeated wing clicks (repetition period 89 ± 15 ms and repetition frequency approx. 11 Hz), which ends with a loud tymbal echeme with increasing amplitude (duration 186 ± 26 ms). After a short pause, the wing clicks of the next phrase begin again. The first interval between the clicks is usually a bit longer and the last 2-6 intervals of a phrase are even longer. The interval between the last click and a tymbal echeme is extremely variable and can last between 60 and 600 ms. The frequency spectrum of tymbal echemes is similar to the spectrum of a continuous song, but the wing clicks have a wider frequency spectrum (2-20 kHz) with a maximum amplitude between 3 and 7 kHz (Gogala and Trilar 1998). The clicks always occur in connection with the opening and closing of the forewings. The hindwings remain more or less in the same position during this cycle (Gogala and Trilar 2003). The males singing close together often synchronise the phrases almost simultaneously, so that the tymbal echemes appear, but are slightly shifted for about 50-150 ms (Gogala and Trilar 1998).

In addition to these two types of song, a few irregular tymbal echemes are recorded with the same spectrum as the continuous song, usually before the cicadas flew away. This may be an alarm song (rivalry song or distress call) by animals singing or courting too close together (Gogala and Trilar 1998).

Selected sound samples of *Cicadatra persica* are available on the web pages *Songs of the European singing cicadas* (Gogala 2020).

**Materials**: Suppl. material 5

##### Diagnosis

*Cicadatra persica* (Fig. 22) is an ecologically very peculiar species that lives on steep slopes of river gorges overgrown with thermophilic Mediterranean vegetation.

#### Cicadatra platyptera

Fieber, 1876

3A698F03-BAE0-5FE4-A023-8220E27DA6DE

##### Distribution

**General distribution: Southern Europe**: Greece, North Macedonia; **Middle East**: Iran, Israel, Jordan, Lebanon, Palestine, Syria, Turkey (summarised by Duffels and van der Laan 1985 and Sanborn 2014).

The citation of the distribution in Italy (Lodos and Kalkandelen 1981, Mozaffarian and Sanborn 2010) is most likely a mistake, since the species was not found in recent bioacoustic fieldwork (Thomas Hertach, personal communication).

**Distribution in Bulgaria**: *Cicadatra platyptera* was found for the first time in Bulgaria at seven localities in eastern Danubian Plane, Upper Thracian Plain and Tundzha-Burgas Valley (Fig. 28) with an altitude distribution between 60 and 194 m (Fig. 29).

##### Notes

**Acoustic behaviour**: In *Cicadatra platyptera* three types of songs are registered: calling song, courtship song and alarm song (rivalry song or distress call) (Boulard 1995, Gogala et al. 2005, Mol et al. 2013).

*Calling song* (Fig. 30) sometimes begins with irregular echemes of 5–15 ms duration, followed a long pattern of many minutes of regular repetition of echemes (duration 122.7 (50–188) ms) and interecheme intervals (duration 91.2 (40–213) ms), generated by the tymbal (Boulard 1995, Gogala et al. 2005, Mol et al. 2013). The calling song is similar to the closely-related species *Cicadatra atra*, but the repetition rate in *Cicadatra platyptera* is almost twice as high (Gogala et al. 2005, Mol et al. 2013). If the calling song in *Cicadatra platyptera* follows the courtship song, the echeme duration is 133 (75–277) ms and the duration of interecheme interval is 80 (36–212) ms (Mol et al. 2013). The frequency range is from 6.2 to 11.9 kHz with a maximum at 7.9 kHz and 9.75 kHz (Boulard 1995).

*Courtship song* consists of echemes, produced by the tymbal and clicks, produced by wing flapping (Fig. 31), which follow each other (Gogala et al. 2005, Mol et al. 2013). Echeme duration is 104 (83–132) ms, interecheme interval duration 132 (48–176) ms and the wing clicks are in the middle of the intervals between echemes (Gogala et al. 2005, Mol et al. 2013). Courtship song can follow the calling song without interruption, lasts from one minute to many minutes and can switch back to the calling song again without interruption (Mol et al. 2013). The frequency range of echemes is from 5.5 to 12 kHz with a maximum between 6 kHz and 6.5 kHz and amplitude spectra show audible frequencies in the range of 1.7–4.6 kHz with a maximum around 3 kHz (Mol et al. 2013).

Selected sound samples of *Cicadatra platyptera* are available on the web pages *Songs of the European singing cicadas* (Gogala 2020).

**Materials**: Suppl. material 6

##### Diagnosis

*Cicadatra platyptera* (Fig. 27) occurs in steppe habitats in arid regions. It usually sits and sings in grass and low herbaceous vegetation.

#### Cicadettini

Buckton, 1890

F9C289E9-8727-5B4D-8AB8-F56482B8471D

#### Cicadetta montana

s. lat. (Scopoli, 1772)

86118892-CAB8-5679-A508-BE50AB3F3AE1

##### Notes

**General distribution: Northwest Europe**: Denmark, Estonia, Finland, Lithuania, Luxembourg, Norway, Sweden; **Southern Europe**: Bulgaria, Croatia, France, Greece, Italy (including Sicily), Montenegro, North Macedonia, Romania, Slovenia, Serbia, Spain; **Central Europe**: Austria, Belgium, Czech Republic, Germany, Hungary, Netherlands, Poland, Slovakia, Switzerland; **British Isles**: United Kingdom; **Eastern Europe**: Moldavia, Russia, Ukraine; **Middle East**: Iran, Palestine, Turkey; **Transcaucasia**: Azerbaijan, Georgia, Russia (Dagestan); **Central Asia**: Kazakhstan, Kyrgyzstan, Russia (Bashkiria, Chuvashia), Tajikistan; **Siberia**: Russia (Southern Siberia); **Eastern Asia**: China (Heilongjiang, Sichuan), Korea, Russia (Primorsky Krai, Sakhalin) (summarised by Nast 1972, Kudryasheva 1979, Duffels and van der Laan 1985 and Sanborn 2014).

**Distribution in Bulgaria**: In addition to a single literature citation by Yoakimov (1909), the specimens of *Cicadetta montana* s. lat. were found in the BFUS, SOFM and ZISB collections (Fig. 32). With the exception of some specimens of *Cicadetta macedonica*, we could not determine the majority at the specific level only on the basis of morphological characteristics. Additionally, the altitudinal distribution (Fig. 33) of this material with three peaks between sea level and 200 m a.s.l., 400 and 600 m a.s.l. and 1000 and 1200 m a.s.l., shows that within the material in the collections, there is most probably more than one species.

**Materials**: Suppl. material 7

##### Diagnosis

Singing activity is a very important mechanism used by male cicadas to attract females and is therefore species-specific (e.g. Gogala & Trilar 2004). Recent bioacoustic studies have shown that *Cicadetta montana* (Scopoli 1772), once thought to be a single widespread Palearctic cicada species, is, in fact, a complex of morphologically-similar sister species that are best characterised by their song patterns. With the help of bioacoustic methods, we find four species from this complex in Bulgaria: *Cicadetta montana* s. str. (Scopoli, 1772), *Cicadetta brevipennis* Fieber, 1876, *Cicadetta cantilatrix* Sueur and Puissant, 2007 and *Cicadetta macedonica* Schedl, 1999.

#### Cicadetta montana

s. str. (Scopoli, 1772)

E317FB68-D260-524D-95B7-B1927732A017

##### Distribution

**General distribution** (only acoustically-validated data): **Southern Europe**: Croatia (Trilar & Gogala, unpublished data), France (Sueur and Puissant 2007a, Sueur and Puissant 2007b, Wade et al. 2015), Greece (Gogala et al. 2008, Gogala et al. 2009), Italy (Trilar and Hertach 2008, Wade et al. 2015) (including Sicily (Hertach 2011)), Montenegro (Trilar & Gogala, unpublished data), North Macedonia (Gogala and Trilar 1999, Gogala et al. 2005), Romania (Trilar et al. 2006a, Trilar and Gogala 2008), Serbia (Gogala and Trilar 2016), Slovenia (Gogala and Gogala 1999, Gogala and Trilar 2004, Wade et al. 2015); **Central Europe**: Austria (Trilar and Holzinger 2004), Germany (Meineke 2012, Meineke 2015), Hungary (Trilar and Gogala 2012b), Poland (Trilar & Gogala, unpublished data), Switzerland (Hertach 2007, Hertach and Pollini Paltrinieri 2012, Wade et al. 2015); **Eastern Europe**: Russia (Mikhailenko and Benediktov 2016), Ukraine (Mikhailenko and Benediktov 2016); **British Isles**: United Kingdom (Grant 1970, Gogala and Trilar 2004); **Middle East**: Iran (Gogala, unpublished data).

**Distribution in Bulgaria**: These are the first bioacoustically verified data for *Cicadetta montana* s. str. in Bulgaria. This mountain species was found in Bulgaria at 40 localities in the northern lowest hills of the Pre-Balkan, southern Lower Mountain Pre-Balkan, northern Balkan Mts., southern Balkan Mts., Kraishte-Ichtiman, Vitosha Mt., Sarnena Sredna Gora Mt., Osogovo Mt. and Rila Mt. (Fig. 35).

In this survey, the majority of the population was found between 600 and 1400 m (90% of the population) (Fig. 36). It was also found at Byala (290 m a.s.l.) and at Golitsa on Kamchiyska Planina (215 m a.s.l.), which is quite low for this species.

##### Notes

**Acoustic behaviour**: The song was described by Gogala (2002), Gogala (2006), Gogala and Trilar (1999), Gogala and Trilar (2004), Trilar and Holzinger (2004), Sueur and Puissant (2007a), Hertach (2007) and Trilar and Gogala (2007).

The *calling song* (Fig. 37) consists of a single long phrase of buzzing sound that slowly increases in intensity and ends with a sudden decrease in amplitude (Fig. 37B) (Gogala and Trilar 1999, Gogala 2002, Trilar and Gogala 2007). The single long echeme can last half a minute or even up to two minutes, but on average, 43.6 ± 29.7 s (Gogala and Trilar 2004).

Selected sound samples of *Cicadetta montana* s. str. are available on the web pages *Songs of the European singing cicadas* (Gogala 2020).

**Materials**: Suppl. material 8

##### Diagnosis

*Cicadetta montana* s. str. (Fig. 34) inhabits mountain forests, mainly beech forests and also mixed deciduous forests, where some pines or spruces could be found. The males sing high up in the canopies, very often higher than 5 m and up to the treetops. The males sing one or two echemes at the same spot and then fly away for a few tens of metres and start singing at a new spot. Only in the morning, when it starts to sing and in the late afternoon, before it stops singing, it can sing in the same place for a long time.

#### Cicadetta brevipennis

s. lat. Fieber, 1876

F2462452-EE16-5973-AEB6-57C01F547C59

##### Distribution

**General distribution** (only acoustically-validated data): **Southern Europe**: Bulgaria, Croatia, Italy, Romania, Serbia, Slovenia; **Central Europe**: Austria, Hungary (summarised by Hertach et al. 2016).

**Distribution in Bulgaria**: During our research, *Cicadetta brevipennis* s. lat. was found for the first time in Bulgaria and some of the song recordings were used in the research of Hertach et al. (2016). The species was found at 33 localities in the eastern Danubian Plane, southern Lower Mountain Pre-Balkan, southern Lower Mountain Pre-Balkan, southern Balkan Mts., Pirin Mt., Tundzha-Burgas Valley, eastern Rhodope Mts. and Strandzha Mt. (Fig. 39).

In this survey, the majority of the population was found between sea level and 600 m (97% of the population) (Fig. 40). It was also recorded at Tvarditshki Prohod on Eleno-Tvarditshka Planina (1079 m a.s.l.), which is extremely high for this species.

##### Notes

**Acoustic behaviour**: The song was described by Gogala and Trilar (1999), Gogala (2002), Gogala and Trilar (2004), Trilar and Holzinger (2004), Gogala (2006), Trilar et al. (2006a) and Hertach et al. (2016).

The *calling song* (Fig. 41) is a repetitive pattern of a long echeme (duration 4.2 ± 3.3 s) increasing in intensity, followed after a short interval (duration 0.05 s) by a short and loud echeme (duration not exceeding 0.05 s) (Boulard 1995, Gogala 2002, Gogala and Trilar 2004). The next long and short echeme sequence follows after a pause of 1.1 ± 0.3 s (Hertach et al. 2016). The dominant frequency is 14.6 ± 0.8 kHz (Hertach et al. 2016).

Selected sound samples of *Cicadetta brevipennis* are available on the web pages *Songs of the European singing cicadas* (Gogala 2020).

**Materials**: Suppl. material 9

##### Diagnosis

Hertach et al. (2016) based on acoustic, morphological, molecular, ecological and spatial data of *Cicadetta brevipennis* s. lat. (Fig. 38) from Spain, France, Italy, Switzerland, Germany, Austria, Slovenia, Croatia, Serbia, Bulgaria and Romania recognised five lineages. *Cicadetta petryi* (Schumacher, 1924) was recognised as a valid species and *Cicadetta brevipennis* was grouped into three subspecies. However, some populations of *Cicadetta brevipennis* s. lat. from Bulgaria were recognised as separate lineage and may be a completely new taxon, but the dataset was not informative enough for the conclusions (Hertach et al. 2016). In the meantime, we have collected new data from Bulgaria and Greece and further comprehensive analysis is needed to clarify the systematics for this region.

#### Cicadetta cantilatrix

Sueur and Puissant, 2007

7A6D38C8-AD5E-5813-AFB2-6231CF9CB4E6

##### Distribution

**General distribution** (only acoustically-validated data): **Southern Europe**: Bulgaria, France, Italy, Montenegro, North Macedonia, Romania, Serbia, Slovenia; **Central Europe**: Austria, Czech Republic (Malenovský and Lauterer 2017), Germany, Hungary, Poland, Switzerland; **Eastern Europe**: Russia (Benediktov and Mikhailenko 2017) (all data except for the countries with the citation in brackets are summarised by Hertach et al. 2015 and Hertach et al. 2016).

**Distribution in Bulgaria**: In our survey *Cicadetta cantilatrix* was found for the first time in Bulgaria. The species was found in four localities in southern Lower Mountain Pre-Balkan, southern Balkan Mts., Kraishte-Ichtiman and Kyustendil-Blagoevgrad Middle Struma valley (Fig. 43) with altitude distribution between 400 and 1200 m (Fig. 44). The lowest point where the species was found was the Berende izvor of the Nishava River at Stara Mt. (495 m a.s.l.) and the highest point at Tsarvenyano below the Radomir peak at Konyavska Mt. (1168 m a.s.l.).

##### Notes

**Acoustic behaviour**: The song was described by Gogala and Trilar (2004), Hertach (2004), Trilar and Holzinger (2004), Gogala et al. (2005), Trilar et al. (2006b) (all previous citations under the name *Cicadetta cerdaniensis*), Sueur and Puissant (2007b), Hertach (2011) and Benediktov and Mikhailenko (2017).

The *calling song* is a sequence of repeated two-phase echemes (Fig. 45): the first phase is long with low-intensity (duration 120-1030 ms) and the second phase is short high-intensity pulse (duration 23-60 ms) (Sueur and Puissant 2007b, Benediktov and Mikhailenko 2017). At the beginning of the sequence, the low-intensity parts of the echemes are missing or are of the same duration as the high-intensity pulses. Later, the duration of the low-intensity parts increases steadily and is longest at the end of the sequence. Echeme duration and inter-echeme duration are negatively correlated (Hertach 2011), which is not the case in closely-related species. Singing males can emit echemes at equal intervals for 7-10 minutes or even longer (Benediktov and Mikhailenko 2017). Most of the spectral energy in the frequency domain is between 11 kHz and 18 kHz. The dominant frequency is not constant, but does not show a clear modulation pattern and averages 13 kHz (Sueur and Puissant 2007b).

The courtship song and the alarm song (rivalry song or distress call) were described by Benediktov and Mikhailenko (2017).

Selected sound samples of *Cicadetta cantilatrix* are available on the web pages *Songs of the European singing cicadas* (Gogala 2020).

**Materials**: Suppl. material 10

##### Diagnosis

*Cicadetta cantilatrix* (Fig. 42) inhabits variable, well-structured margins of thermophilic forests along extensively used meadows. Males usually sit on low trees or shrubs, sometimes even in the herb layer when they emit their calling song (Hertach 2007).

#### Cicadetta macedonica

Schedl, 1999

7B5EE386-1A22-5653-A768-A793D808D969

##### Distribution

**General distribution: Southern Europe**: Greece, North Macedonia, Romania, Serbia (Gogala and Trilar 2016); **Central Europe**: Czech Republic (Malenovský and Lauterer 2017) (all data except for the countries with the citation in brackets are summarised by Sanborn 2014).

**Distribution in Bulgaria**: *Cicadetta macedonica* was found for the first time in Bulgaria in 50 localities in northern lowest hills of the Pre-Balkan, southern Lower Mountain Pre-Balkan, northern Balkan Mts., southern Balkan Mts., Kraishte-Ichtiman, Pirin Mt., Dabrash-Batak western Rhodope Mts., Prespa-Chernatitsa western Rhodope Mts., eastern Rhodope Mts. and Strandzha Mt. (Fig. 47). The majority of the population is distributed between 400 and 1200 m (90% of the population) (Fig. 48). The lowest point where we have recorded this species is near Kalovo at Mladezhka reka in Strandzha Mt. (162 m a.s.l.) and the highest western Rhodopes near the deviation Batak-Dospat-Sarnitsa (1491 m a.s.l.).

##### Notes

**Acoustic behaviour**: The song was described by Gogala and Trilar (1999), Gogala and Trilar (2004) and Gogala et al. (2008).

The many-minutes lasting *calling songs* of *Cicadetta macedonica* consist of a series of short echemes (SE) with a single long echeme (LE) at the end (Fig. 49). The song sequence usually begins with very long period of SE repetitions lasting over one minute and ends with LE. The sequences of phrases then follow with shorter series of SE followed by one LE. The SE duration is 26 ± 5 ms, the interval between them 63.4 ±12 ms, the repetition frequency is about 11 ± 1 Hz and the LE duration is 161 ± 20 ms. The next phrase starts after an interval of half a second. The song contains frequencies from 5 to 20 kHz with a maximum between 12 and 14 kHz (Gogala and Trilar 1999, Gogala and Trilar 2004, Gogala et al. 2008).

Selected sound samples of *Cicadetta macedonica* are available on the web pages *Songs of the European singing cicadas* (Gogala 2020).

**Materials**: Suppl. material 11

##### Diagnosis

*Cicadetta macedonica* (Fig. 46) inhabits sparse thermophilic forests, mainly oak forests and also mixed deciduous forests. The males sing in the trees on prominent branches, but also on isolated smaller trees or shrubs or even in the grass in forest clearings, where females gather to find mating partners (Gogala and Trilar 1999).

#### Dimissalna dimissa

(Hagen, 1856)

87E55C52-1DCC-5931-A5E5-BDF03F56202C

##### Distribution

**General distribution: Southern Europe**: Albania, Bosnia (Puissant and Sueur 2011), Bulgaria, Croatia, France (Puissant and Sueur 2011, Gurcel 2011, Puissant 2012), Greece, Italy, Montenegro (Trilar & Gogala, unpublished data), North Macedonia, Romania, Serbia (Janković 1966, Lekić 1966, Janković 1975, Gogala and Trilar 2016), Slovenia; **Eastern Europe**: Russia (South European Russia) (Popov 1975); **Middle East**: Israel, Syria, Turkey; **Transcaucasia**: Armenia, Azerbaijan, Russia (Chechnya); **Central Asia**: Kazakhstan, Russia (Siberia) (all data except for the countries with the citation in brackets are summarised by Duffels and van der Laan 1985 and Sanborn 2014).

The records from Kazakhstan and Central Asia are doubtful as they could refer to unidentified Central-Asiatic closely-related species (Tishechkin, personal communication). There are doubtful records for Sicily (Servadei 1967, Nast 1972) and Crete (Nast 1972), since *Dimissalna dimissa* was never found in recent fieldwork with bioacoustic methods on these Islands (Thomas Hertach, personal communication, Trilar and Gogala 2010, Trilar and Gogala 2012a). The presence of *Dimissalna dimissa* in China, as reported by Nast (1972), can be considered highly doubtful, as it has not been mentioned in any recent work on Chinese cicadas (e.g. Chou et al. 1997).

**Distribution in Bulgaria**: *Dimissalna dimissa* is the second most common and widespread species of singing cicada distributed in Bulgaria. Data are known for 122 localities (Fig. 51). In literature, we found data for Bulgaria in Nedyalkov (1908) and Háva (2016). Arabadzhiev (1963) considers *Dimissalna dimissa* as a pest in the orchards in the area of Pavlikeni. The species is also listed in the overviews by Nast (1972), Nast (1987) and Holzinger et al. (2003), but without precise locality data for Bulgaria.

In Bulgaria, *Dimissalna dimissa* is a generally distributed species with known data in western Danubian Plane, eastern Danubian Plane, northern lowest hills of the Pre-Balkan, southern Lower Mountain Pre-Balkan, northern Balkan Mts., Kraishte-Ichtiman, Osogovo Mt., Belasitsa Mt., Osogovo Mt., Kyustendil-Blagoevgrad Middle Struma valley, Sandanski-Petrich Middle Struma valley, Pirin Mt., Gotse Delchev Mesta valley, Dabrash-Batak western Rhodope Mts., Prespa-Chernatitsa western Rhodope Mts., Upper Thracian Plain, Tundzha-Burgas Valley, Eastern Rhodope Mts., Haskovo Hills Land, Sakar Mt. and Strandzha Mt. (Fig. 51).

In this survey, the majority of the population was found between sea level and 1000 m (98% of the population) (Fig. 52). Only three localities were above this limit. We found *Dimissalna dimissa* near Sinchets (1009 m a.s.l.). In the ZISB collection, one female is kept from Mt. Vedernik peak near Belogradchik (1080 m a.s.l.), collected by B. Zaharieva and the other female from Mt. Persenk in western Rhodopes (1680 m a.s.l.) collected by V. Bayryamova.

##### Notes

**Acoustic behaviour**: The song was described by Popov (1975) and Gogala and Popov (2000). Sound emission consists of two types of songs, between which the animals can switch without interruption (Popov 1975, Gogala and Popov 2000).

The first type of song (Fig. 53) can last from a few seconds to many minutes and consists of a sequence of short, identical phrases made up of 5-6 short echemes (duration (d) 4.7-6.3 ms, repetition rate (rr) 43.1 Hz) and one long echeme (d = 25.8-59.3 ms, rr = 5.7 Hz) (Fig. 53B) (Gogala and Popov 2000).

The second type (Fig. 54) also lasts for several minutes and comprises a series of very complex sequences (d = 4.5-9.4 s). Each sequence consists of four segments that follow each other in a strictly defined order. The first segment (Fig. 54C) (d = 0.65-2.62 s) consists of a simple series of short echemes (d = 31.1 ± 5.8 s, rr = 32.2 Hz). In the second segment (Fig. 54C) (d = 2 ± 0.7 s), the pattern is similar to the first type of song, but the number of short echemes and the duration of long echemes increases gradually (from 13-16 ms at the beginning to 60-71 ms at the end). In the third segment (Fig. 54D) (d = 1.55-4.0 s) the echemes finally merge into a continuous buzzing sound. After a short pause (d = 37.4 ± 5 ms), the final long echeme (Fig. 54D) (d=70.7±6.9 ms) follows and, after a short interval (d = 123 ± 24 ms), the entire sequence starts again. The frequency band of sound emission is between 10 and 18.5 kHz with a peak around 13 kHz (Popov 1975, Gogala and Popov 2000).

Selected sound samples of *Dimissalna dimissa* are available on the web pages *Songs of the European singing cicadas* (Gogala 2020).

**Materials**: Suppl. material 12

##### Diagnosis

*Dimissalna dimissa* (Fig. 50) emits sound in high frequencies, usually in the community of many other lower pitched and loud singing cicada species, so it is difficult to hear them without ultrasonic detectors and therefore is often overlooked. The animals usually live on trees or tall shrubs and can be found high up in the tree canopies from 2-4 m and up to the treetops (Popov 1975, Trilar and Gogala 2008, Puissant 2012).

#### Oligoglena tibialis

(Panzer, 1798)

026C3AA9-F364-54F5-B23C-2B1D9027FA08

##### Distribution

**General distribution: Southern Europe**: Albania, Bosnia, Bulgaria, Croatia, France, Greece, Italy, North Macedonia, Portugal, Romania, Slovenia, Serbia, Spain; **Central Europe**: Austria, Czech Republic, Hungary, Poland, Slovakia; **Eastern Europe**: Moldova, Russia (South European Russia), Ukraine; **Middle East**: Iran, Israel, Jordan, Lebanon, Turkey; **Transcaucasia**: Azerbaijan, Georgia, Russia (Chechnya); **Central Asia**: Kyrgyzstan, Uzbekistan; **Northern Africa**: Morocco, Tunisia (summarised by Duffels and van der Laan 1985 and Sanborn 2014).

There is a doubious citation of *Oligoglena tibialis* for Germany (Kaltenbach et al. 1972, Nast 1972, Lodos and Kalkandelen 1981, Nast 1987, Quartau and Fonseca 1988), as it is not listed in the latest overview of fauna (Nickel et al. 2016). There are also doubtful citations for Sicily (Nast 1972, Quartau and Fonseca 1988) and Crete (Nast 1972, Quartau and Fonseca 1988), as *Oligoglena tibialis* has never been found in recent field research with bioacoustic methods on these Islands (Thomas Hertach, personal communication, Trilar and Gogala 2010, Trilar and Gogala 2012a).

**Distribution in Bulgaria**: This small cicada is the most abundant and most widespread species of singing cicadas found in Bulgaria. The data for 125 localities are known (Fig. 56). In literature, we found the data in Nedyalkov (1908), Yoakimov (1909), Dlabola (1955), Bairyamova (1976) and Pelov (1968). Bairyamova (1992) lists the species for Rhodope Mts. for xerophylous and mesophylous oak-horn forests *(Quercus-Carpinus)* between 700 and 1000 m and beech forests *(Fagus)* between 1000 and 1400 m and Bairamova (1978) mentions the species for oak forests *(Quercus)*. The species is also listed in overviews by Nast (1972), Nast (1987), Lodos and Kalkandelen (1981) and Quartau and Fonseca (1988), but without exact location data for Bulgaria.

In Bulgaria, it is distributed all over the country with known data in western Danubian Plane, eastern Danubian Plane, northern lowest hills of the Pre-Balkan, southern Lower Mountain Pre-Balkan, northern Balkan Mts., western Sub-Balkan valleys, Kraishte-Ichtiman, Vitosha Mt., True Sredna Gora Mt., Ograzhden-Vlachina Mts., Kyustendil-Blagoevgrad Middle Struma valley, Sandanski-Petrich Middle Struma valley, Pirin Mt., Dabrash-Batak western Rhodope Mts., Prespa-Chernatitsa western Rhodope Mts., Upper Thracian Plain, Tundzha-Burgas Valley, eastern Rhodope Mts., Haskovo Hills Land, Sakar Mt. and Strandzha Mt. (Fig. 56).

In this study, the majority of the population of *Oligoglena tibialis* was found between sea level and 800 m (91% of the population) (Fig. 57), but the rest is distributed between 801 and 1400 m. The highest find is registered at 1570 m a.s.l., where an unknown collector in Pamporovo collected one male and one female specimen, which are kept in the SOFM collection.

##### Notes

**Acoustic behaviour**: The song was described by Popov (1975), Joermann and Schneider (1987), Boulard (1995), Gogala et al. (1996) and Sueur and Puissant (2000).

The calling song contains two types of phrases (Fig. 58). The first phrase (duration 920 ± 89 ms) is a sequence of short echemes (SE) (duration 48 ± 8 ms) followed by a long echeme (LE) (duration 292 ± 50 ms). Usually groups of short first phrases with 2-5 SE are separated by longer first phrases with a higher number of SE (11-13), but there is little regularity in this pattern. The second phrase consists of regularly repeated SE (duration 54 ± 11 ms, 38-76 SE) (Gogala et al. 1996, Joermann and Schneider 1987). The first phrase could last for minutes, then the animal suddenly switches to a second phrase, which could last only a few or ten seconds, then the first phrase appears again. It has often been observed that, after the second phrase, the males stopped singing and flew away. The spectrum of the calling song contains two frequency bands: a main band between 12 and 22 kHz with a maximum between 14 and 18 kHz and sometimes with a secondary peak near 12 kHz and a second band with a maximum between 7 and 8 kHz (Gogala et al. 1996).

Selected sound samples of *Oligoglena tibialis* are available on the web pages *Songs of the European singing cicadas* (Gogala 2020).

**Materials**: Suppl. material 13

##### Diagnosis

*Oligoglena tibialis* (Fig. 55) is one of the smallest singing cicadas in Europe, usually found on bushes and small trees. In areas where the shrubs were predominantly occupied by another species, i.e. *Tettigettula pygmea*, which has a song of a similar spectrum, *Oligoglena tibialis* is regularly found singing in meadows, alfalfa fields and on other green plants. The males sing all day long when the weather is fine and the ambient temperature is not too low (above 20°C). The males chirp their calling song from one place, a small branch or leaf, for one or more minutes, then fly away, find another position and start singing again (Gogala et al. 1996).

#### Tettigettula pygmea

(Olivier, 1790)

107A6085-7EB8-57B5-91D5-7AF7A3BF630F

##### Distribution

**General distribution: Southern Europe**: Albania, Austria (Schedl 2004), Croatia, France, Greece, Italy (including Sicily (Hertach 2011)), North Macedonia, Romania, Serbia (Horváth 1903, Janković 1966, Janković 1975, Gogala and Trilar 2016), Slovenia (all data except for the countries with the citation in brackets are summarised by Duffels and van der Laan 1985 and Sanborn 2014).

It was not found in Spain (Puissant 2006, Puissant and Sueur 2010) and on the eastern side of Rechinger’s line in the Aegean Sea (Gogala and Trilar 2014), which represents the zoogeographical border between the European and Asian fauna.

**Distribution in Bulgaria**: *Tettigettula pygmea* was found for the first time in Bulgaria at three localities in Strandzha Mt. (Fig. 60) with an altitude distribution between the sea level and 200 m (18-146 m a.s.l.) (Fig. 61).

##### Notes

**Acoustic behaviour**: The song was described by Boulard (1995), Popov et al. (1997), Gogala (2002) and Puissant and Sueur (2010).

The *calling song* consists of two phrases with repeating patterns (Figs 62, 63). In the first phrase (duration 250-500 ms), the long echeme (duration 175 ± 41 ms) is usually followed by 2-4 short echemes (duration 5-6 ms) (Fig. 62). The second phrase (duration 320-560 ms) also consists of a long echeme (duration 273 ± 41 ms), followed by one longer short echeme (duration 60 ± 6 ms) or without it (30% of the phrases) (Fig. 63) (Popov et al. 1997). Sequences of the first phrase can change to the second phrase without interruption (Popov et al. 1997, Gogala 2002). The main energy of both singing patterns is between 15 and 25 kHz, mainly in inaudible ultrasound (Figs 62, 63), therefore the use of an ultrasonic detector is appropriate (Popov et al. 1997, Gogala 2002).

Selected sound samples of *Tettigettula pygmea* are available on the web pages *Songs of the European singing cicadas* (Gogala 2020).

**Materials**: Suppl. material 14

##### Diagnosis

*Tettigettula pygmea* (Fig. 59) is the smallest European singing cicada. It sings in the high frequency range (i.e. Popov et al. 1997), usually high up in the tree canopies, especially in oaks (Gogala 2002), usually higher than 2 m (Puissant 2006). Only in some areas are the shrubs predominantly populated. The species inhabit warm Mediterranean vegetation (Puissant 2006).

#### Pagiphorini

Moulds & Marshall, 2018

E9269CAB-F29A-553C-938B-F01705EB8D8F

#### Pagiphora annulata

(Brullé, 1832)

6F3A5B2D-B279-5CD3-8B17-45C925EFCFFC

##### Distribution

**General distribution: Southern Europe**: Albania, Bulgaria, Greece, Moldova, North Macedonia, Romania, Serbia, Spain; **Central Europe**: Hungary, Slovakia; **Middle East**: Turkey (summarised by Duffels and van der Laan 1985 and Sanborn 2014).

The populations from north Africa (Algeria, Tunisia) (Nast 1972) were later described as *Pagiphora maghrebensis* Boulard, 1981 (Boulard 1981a, Boulard 1992b).

One of the biodiversity hotspots for the genus *Pagiphora* is in Turkey, where at least three species can be found. *Pagiphora annulata* (Brullé, 1832) is distributed in the northwest at least in Hatay Province (Linnavuori 1965, Demir 2008), Izmir Province (Lodos and Kalkandelen 1981, Demir 2008) and Aydim Province (Trilar & Gogala, unpublished data). *Pagiphora yanni* Boulard, 1992 occurs in the south at least in Adana Province (Boulard 1992a, Boulard 1993, Demir 2008) and Mersin Province (Trilar, unpublished data). For the south-western part of Turkey (Antalya Province) Kartal (1983) and Demir (2007) cite *Pagiphora aschei* Kartal, 1978 which is most likely an endemic species of the Greek Island Crete (Trilar and Gogala 2012a). *Pagiphora hauptosa* Boulard, 1981 was also described from Antalya Province and the song recordings from this area show that this population is acoustically different from the Cretan population (Trilar, unpublished data). This species richness and the peculiar distribution in Turkey raises the question, which *Pagiphora* species are distributed in the Middle East (Iran, Syria) and Central Asia (Kyrgyzstan, Uzbekistan).

**Distribution in Bulgaria**: In Bulgaria, data are available for 45 localities (Fig. 65). In literature, we found the data for Bulgaria in Dlabola (1955). Arabadzhiev (1963) considers *Pagiphora annulata* as a serious pest in the orchards in the area of the Pavlikeni and gives the recipe for eradication by spraying. The species is also listed in overviews by Nast (1972), Nast (1987) and Lodos and Kalkandelen (1981), but without the exact location data for Bulgaria.

In Bulgaria, *Pagiphora annulata* is scattered all over the country, with the known data in eastern Danubian Plane, northern lowest hills of the Pre-Balkan, northern Balkan Mts., eastern Sub-Balkan valleys, Kyustendil-Blagoevgrad Middle Struma valley, Rila Mt., Tundzha-Burgas Valley, eastern Rhodope Mts., Haskovo Hills Land and Strandzha Mt. (Fig. 65).

In this survey, the majority of the population was found between sea level and 600 m (93% of the population) (Fig. 66). The highest point where we recorded the species was Polska Skakavitsa at Zemenska Mt. (817 m a.s.l.).

##### Notes

**Acoustic behaviour**: The characteristic of the song emission of *Pagiphora annulata* (the same applies to all other species of the genus *Pagiphora*) is a surprisingly low frequency band for such relatively-small animals. According to Bennet-Clark and Young (1994), the emitted frequency of such small cicadas should be about 12 kHz, but for *Pagiphora annulata*, the main energy was measured between 3 and 4.5 kHz (Gogala and Trilar 2000, Gogala et al. 2005).

The song was described by Gogala and Trilar (2000) and wing clicking by Gogala and Trilar (2003).

The *calling song* consists of phrases (duration 2.1-2.9 s), consisting of 7 to 10 echemes, whose duration and intensity increases towards the end of a phrase (Fig. 67). The repetition frequency of the echemes in a phrase is 3 ± 0.5 Hz. Phrases are repeated irregularly, usually after an interval of about 2 to 10 seconds. The frequency band of these sounds is surprisingly low for small cicadas, from 3 to 4.5 kHz with a peak around 3.9 kHz (Gogala and Trilar 2000).

The second part of the last 3 to 5 echemes is accompanied by wing movements and the sound is louder and sharper. The spectrograms show a wider spectrum and oscillograms show the additional short sound pulses (repetition frequency 130-140 Hz) only roughly synchronised with the regular tymbal pulses (Fig. 67B marked with arrows). A high-speed video analysis proved that the additional clicks with a wide frequency range occur only in connection with wing flapping (last 3 to 5 echemes) and that the wings remain in closed position in the tymbal generated vocal parts (first 4 to 6 echemes) (Gogala and Trilar 2003).

Selected sound sample of *Pagiphora annulata* is available on the web pages *Songs of the European singing cicadas* (Gogala 2020).

**Materials**: Suppl. material 15

##### Diagnosis

*Pagiphora annulata* (Fig. 64) is one of the smallest European singing cicada that are normally found on shrubs and small trees. They are also found on olive trees, fruit trees and in gardens.

#### Tibicininae

A475E9E5-A609-51D1-860B-1EEA30FDA69E

#### Tibicinini

Distant, 1905

E890495B-544E-5FDF-B9C1-182F6AE0D70D

#### Tibicina haematodes

(Scopoli, 1763)

FAC7ADFC-FE74-5C37-BA0D-23BA553B0C4D

##### Distribution

**General distribution: Southern Europe**: Albania, Bulgaria, Croatia, France, Greece, Italy, Moldova, North Macedonia, Portugal, Romania, Serbia, Slovenia, Spain; **Central Europe**: Austria, Czech Republic, Germany, Hungary, Slovakia, Switzerland; **Eastern Europe**: Russia (south European Russia), Ukraine; **Middle East**: Iran, Palestine, Turkey; **Transcaucasia**: Armenia, Azerbaijan, Georgia (summarised by Duffels and van der Laan 1985 and Sanborn 2014).

There are dubious citations for Corsica (Nast 1972, Quartau and Fonseca 1988) and Sicily (Servadei 1967, Nast 1972, Quartau and Fonseca 1988), as*Tibicina haematodes* was never found during recent fieldwork with bioacoustic methods on these Islands (Thomas Hertach, personal communication).

**Distribution in Bulgaria**: This colourful cicada was found in many localities in Bulgaria and the data are known for 45 localities (Fig. 69). In literature, we found the data for Bulgaria in Yoakimov (1909) and Pelov (1968). Nedyalkov (1908) lists the species for northern and southern Bulgaria and points out that they are less common than *Lyristes plebejus*. Arabadzhiev (1963) considers *Tibicina haematodes* as a pest in orchards in the area of Pavlikeni. The species is also listed in overviews by Nast (1972), Schedl (1986), Nast (1987) and Quartau and Fonseca (1988), but without the exact location data for Bulgaria.

In Bulgaria, it is generally distributed with known data in western Danubian Plane, eastern Danubian Plane, northern lowest hills of the Pre-Balkan, southern Lower Mountain Pre-Balkan, northern Balkan Mts., western Sub-Balkan valleys, eastern Sub-Balkan valleys, Kraishte-Ichtiman, Sandanski-Petrich Middle Struma valley and eastern Rhodope Mts. (Fig. 69).

In this survey, the majority of the population was found between sea level and 600 m (91% of the population) (Fig. 70). At the highest point, one male was found on the Kachulka peak from Slivenski Balkan (1072 m a.s.l.) collected by N. Atanassov, which is kept in the SOFM collection.

##### Notes

**Acoustic behaviour**: The song recorded during our fieldwork in Bulgaria does not differ from the pattern recorded, analysed and described by Boulard (1990), Boulard (1995), Boulard and Mondon (1995), Gogala (2002), Sueur and Aubin (2002) and Sueur and Aubin (2003).

The *calling song* (Fig. 71) begins with 1 to 8 introductory echemes (average 3.6 ± 1.6), which continue in a simple monotonous buzz lasting from 15 seconds to half a minute, averaging 14.0 ± 2.3 s (Sueur and Aubin 2002, Sueur and Aubin 2003). The complete song is repeated after a pause of approximately the same length (Gogala 2002). The buzz is made up of groups of pulses (Fig. 71C) consisting of 6-8 pulses arranged in two subgroups of 3-4 pulses (Fig. 71D). The number in the group of pulses is stable within and between individuals. The pulse group generates a large amplitude modulation at a rate of 98.3 ± 1.6 per second, the pulse-group duration is 8.2 ± 0.4 ms and the pulse-group period is 10.2 ± 0.4 ms. The single pulse lasts about 1 ms and generates a fast amplitude modulation at a rate of about 1000 Hz. The frequency range is about 5.2 to 8.0 kHz with three main peaks: one band with higher energy at about 7.4 kHz and two sidebands at about 6.4 and 8.4 kHz (Boulard 1995, Sueur and Aubin 2002, Sueur and Aubin 2003).

Selected sound samples of *Tibicina haematodes* are available on the web pages *Songs of the European singing cicadas* (Gogala 2020).

**Materials**: Suppl. material 16

##### Diagnosis

*Tibicina haematodes* (Fig. 68) is the most colourful European cicada and one of the largest and loudest European species of the singing cicada. The species occurs mainly in closed high shrubland and woodland on deciduous trees. The males usually sing high up in the tree canopies.

#### Tibicina steveni

(Krynicki, 1837)

51370C9F-E1E0-5CF6-87A0-64E8F44A3646

##### Distribution

**General distribution: Southern Europe**: Bulgaria, France, Greece (Gogala and Trilar 2014), Italy (Hertach and Nagel 2013); **Central Europe**: Switzerland; **Eastern Europe**: Russia (South European Russia), Ukraine; **Middle East**: Turkey; **Transcaucasia**: Armenia, Azerbaijan, Georgia (all data except for the countries with the citation in brackets are summarised by Duffels and van der Laan 1985 and Sanborn 2014).

**Distribution in Bulgaria**: This species is reported for Bulgaria as literature data, but without the citation of the source, by Janković (1971) for Petrich, which was erroneously placed in North Macedonia (Gogala et al. 2005). Petrich is a town in the Strumeshnitsa valley in SW Bulgaria, not far from the North Macedonian border. We recorded the singing males of this species on 23 July 2008 near Bozhkinoto on the slope of Belasitsa Mt. and confirmed their presence in this area.

In Bulgaria, data are known for 32 localities (Fig. 73). *Tibicina steveni* is distributed in the southern part of the country along the Greek and Turkish borders. The data are known from Belasitsa Mt., Tundzha-Burgas Valley, eastern Rhodope Mts., Haskovo Hills Land, Sakar Mt. and Strandzha Mt. (Fig. 73) with altitude distribution between 20 and 820 m a.s.l. (Fig. 74).

##### Notes

**Acoustic behaviour**: The song was described by Popov (1975), Sueur et al. (2003) and Sueur and Aubin (2003).

The calling song (Fig. 75) is a long, simple, monotonous buzz with easily audible fibrillation, lasting from one minute to many minutes without interruption, on average 186 ± 187 s (Sueur et al. 2003, Hertach 2020). It consists of a long, sustained sequence of groups of pulses (Fig. 75C) with a slow increase in amplitude at the beginning. The groups of pulses are generated at a rate of 58.5 ± 3.9 per second, the pulse-group duration is 12.3 ± 1.3 ms and the pulse-group period is 16.8 ± 1.0 ms. The pulse-groups consists of 9-10 pulses (Fig. 75D) (Sueur and Aubin 2003, Sueur et al. 2003). The frequency range of the calling song is between 5.5 and 9.5 kHz with three frequency peaks: a frequency band with the highest energy at 7.2 kHz with two lateral frequency bands at about 6.4 kHz and 8.2 kHz, the lateral bands being generated by pulse amplitude modulations (Sueur et al. 2003).

Selected sound sample of *Tibicina steveni* is available on the web pages *Songs of the European singing cicadas* (Gogala 2020).

**Materials**: Suppl. material 17

##### Diagnosis

*Tibicina steveni* (Fig. 72) is one of the largest European species of the singing cicada. The species occurs mainly in closed high shrubland and woodland on deciduous trees such as oaks, sycamores and chestnuts. The males usually sing high up in the tree canopies.

## Discussion

Due to extensive field research and the use of bioacoustic methods, the number of known cicada species in Bulgaria has increased in recent years and the knowledge about ecology and distribution has improved.

The three ubiquitous, loudest and largest singing cicadas with spectacular songs, *Cicada orni*, *Lyristes plebejus* and *Tibicina haematodes*, were recorded acoustically and also collected in many localities in Bulgaria. Some other species were not so easy to detect and locate acoustically and especially for the *Cicadetta montana* group of sister species, ultrasonic detectors were needed to confirm their presence.

During the fieldwork for this survey, we recorded and/or collected 15 species: *Cicada orni*, *Lyristes plebejus*, *Cicadatra atra*, *Cicadatra hyalina*, *Cicadatra platyptera*, *Cicadetta montana* s. str., *Cicadetta brevipennis* s. lat., *Cicadetta cantilatrix*, *Cicadetta macedonica*, *Dimissalna dimissa*, *Oligoglena tibialis*, *Tettigettula pygmea*, *Pagiphora annulata*, *Tibicina haematodes* and *Tibicina steveni*.

There are published records for 10 of them: *Cicada orni* (Nedyalkov 1908, Yoakimov 1909, Nast 1972, Sander 1985, Nast 1987, Bairyamova 1992), *Lyristes plebejus* (Nedyalkov 1908, Yoakimov 1909, Arabadzhiev 1963, Nast 1972, Sander 1985, Nast 1987, Bairyamova 1992, Háva 2016), *Cicadatra atra* (Nedyalkov 1908, Yoakimov 1909, Dirimanow and Harisanow 1965, Nast 1972, Sander 1985, Nast 1987, Lodos and Kalkandelen 1981), *Cicadatra hyalina* (Nedyalkov 1908), *Cicadetta montana* s.lat. (Yoakimov 1909, Nast 1972, Nast 1987), *Dimissalna dimissa* (Nedyalkov 1908, Arabadzhiev 1963, Nast 1972, Nast 1987, Holzinger et al. 2003, Háva 2016), *Oligoglena tibialis* (Nedyalkov 1908, Yoakimov 1909, Dlabola 1955, Pelov 1968, Nast 1972, Bairamova 1978, Bairyamova 1976, Lodos and Kalkandelen 1981, Nast 1987, Bairyamova 1992), *Pagiphora annulata* (Dlabola 1955, Arabadzhiev 1963, Nast 1972, Lodos and Kalkandelen 1981, Nast 1987), *Tibicina haematodes* (Nedyalkov 1908, Yoakimov 1909, Arabadzhiev 1963, Pelov 1968, Nast 1972, Schedl 1986, Nast 1987, Quartau and Fonseca 1988) and *Tibicina steveni* (Janković 1971, Gogala et al. 2005).

In the public collections of the National Museum of Natural History (SOFM), the Institute of Zoology (ZISB) and the Biology Faculty of Sofia University »St. Kliment Ohridski« (BFUS), which are all located in Sofia, we found, besides the nine species already mentioned in literature, three more species: *Cicadatra persica*, *Cicadatra platyptera* and *Cicadetta macedonica*. For all three species, the occurrence in Bulgaria has not yet been published.

**Figure 5.**
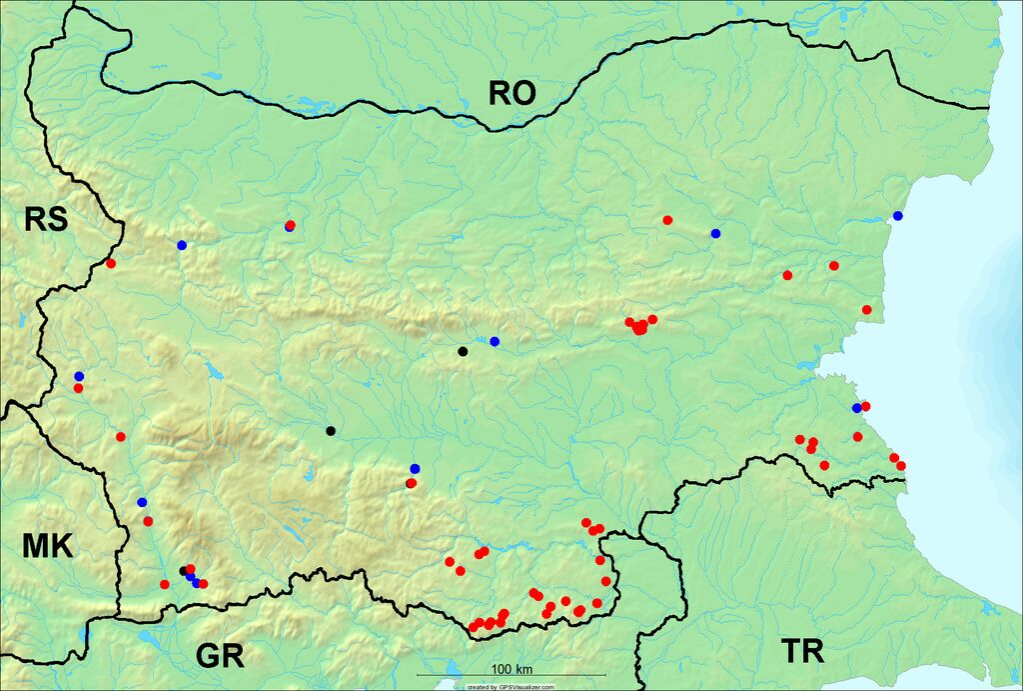
**Map with localities of *Cicada orni* in Bulgaria.** Black - literature data, blue - data from collections, red - bioacoustic data collected in this survey.

**Figure 6.**
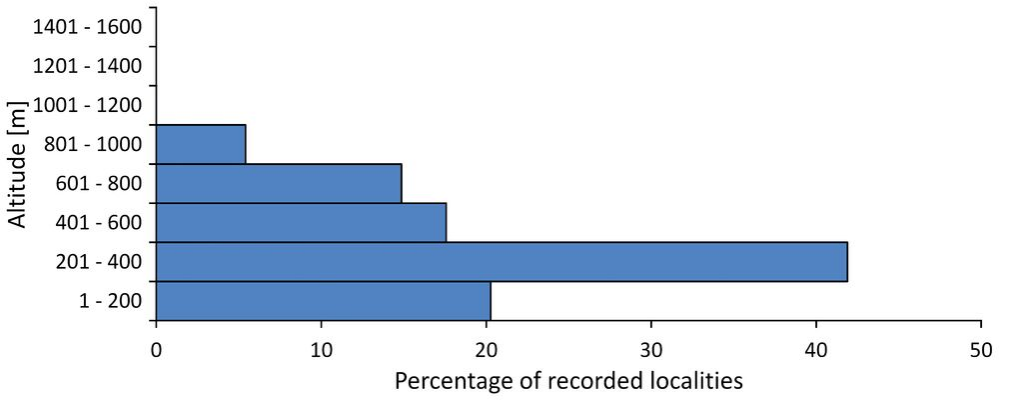
**Altitudinal distribution of *Cicada orni*.** The percentage of recorded localities is shown in 200-metre altitude zones.

**Figure 7.**
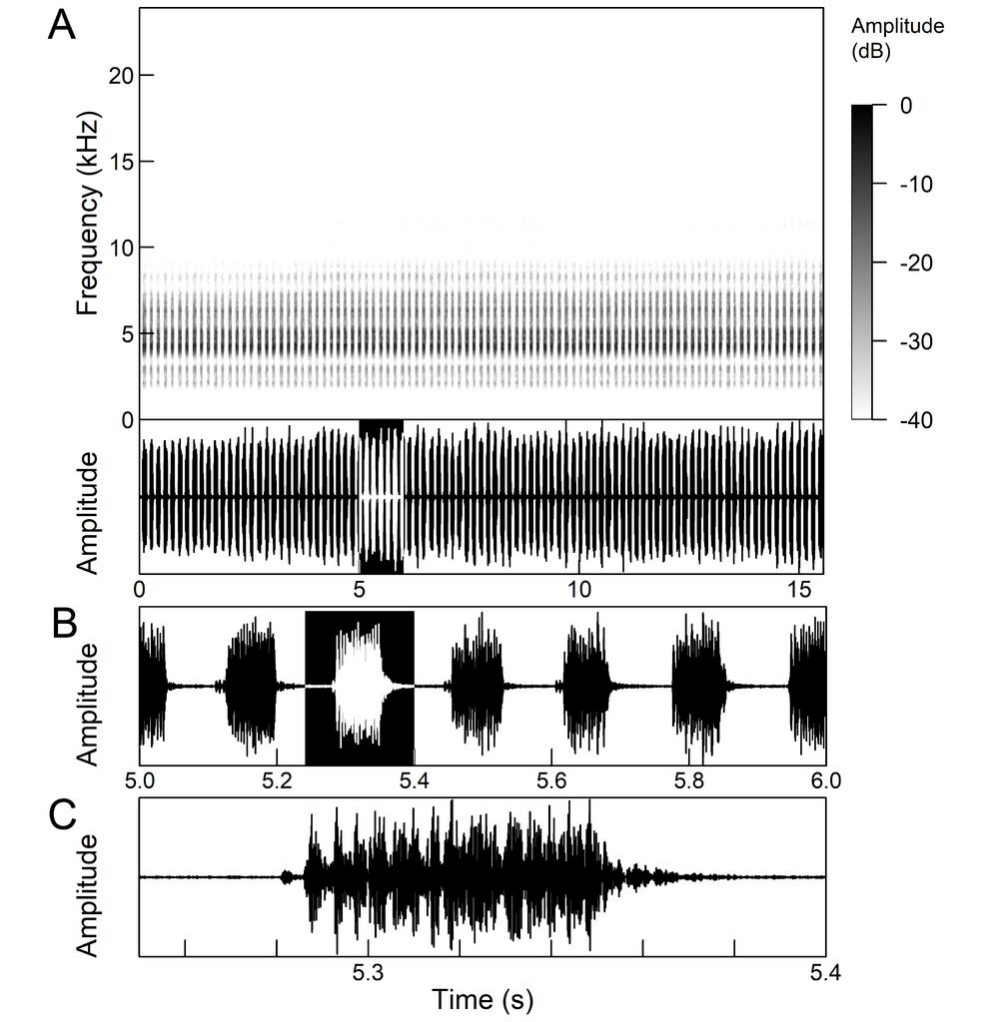
**Calling song of *Cicada orni*.** (A) spectrogram (time *vs.* frequency *vs.* amplitude, amplitude scale is given on the right) and oscillogram (time *vs.* amplitude) of the selected part of the calling song; (B) oscillogram of six echemes corresponding to the inverted window in (A); (C) oscillogram of one echeme corresponding to the inverted window in (B).

**Figure 8.**
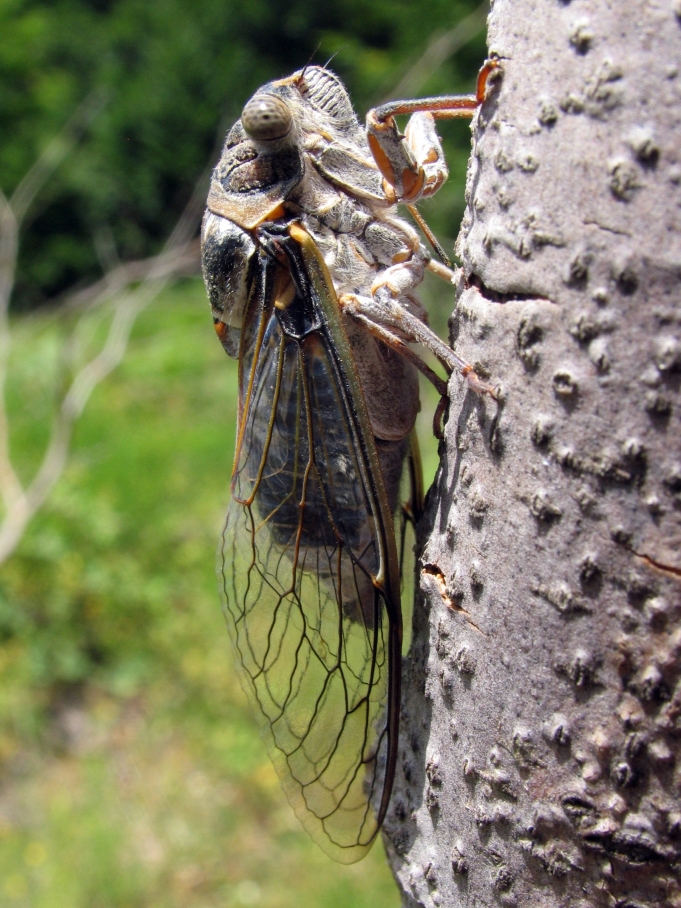
***Lyristes plebejus* male.**

**Figure 9.**
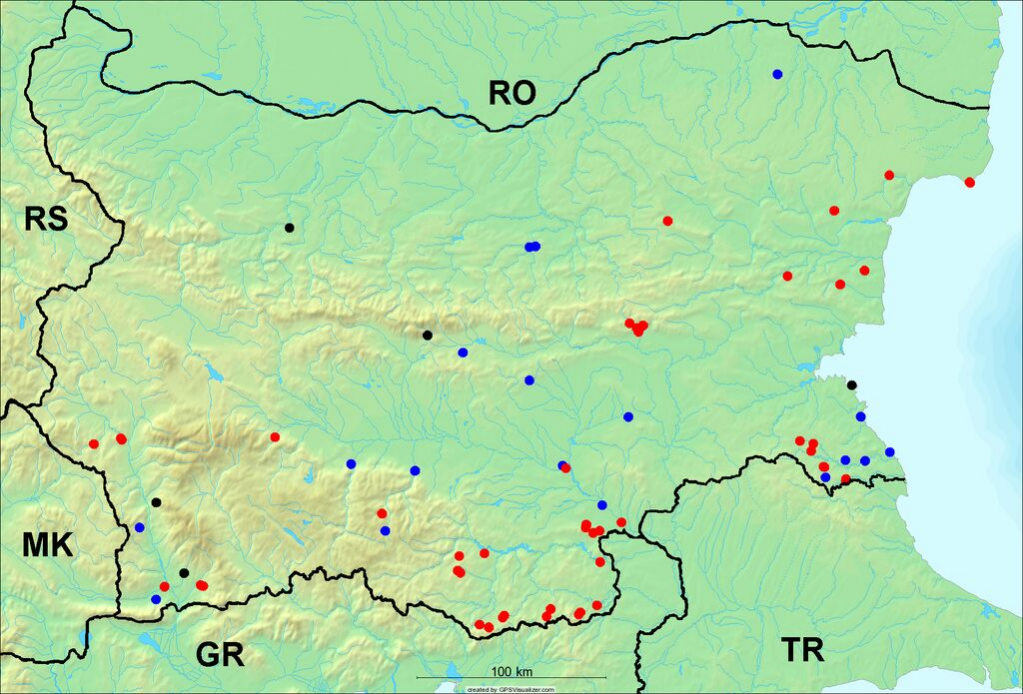
**Map with localities of *Lyristes plebejus* in Bulgaria.** Black - literature data, blue - data from collections, red - bioacoustic data collected in this survey.

**Figure 10.**
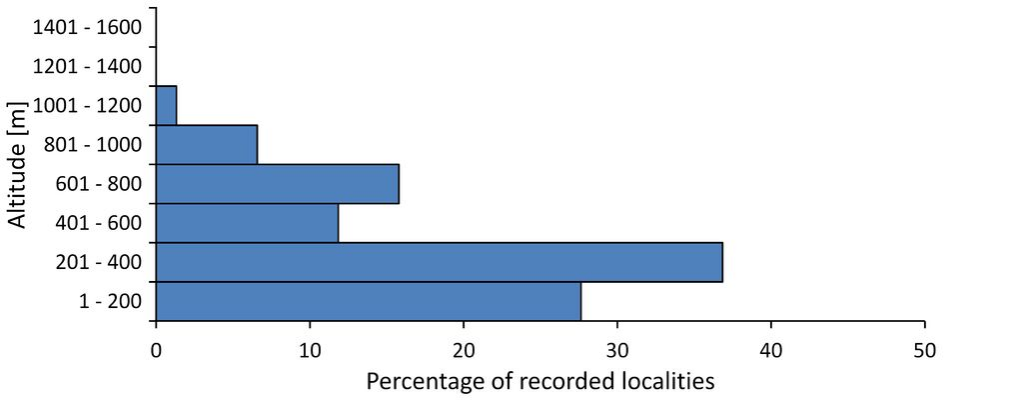
**Altitudinal distribution of *Lyristes plebejus*.** The percentage of the recorded localities is displayed in 200-metre altitude zones.

**Figure 11.**
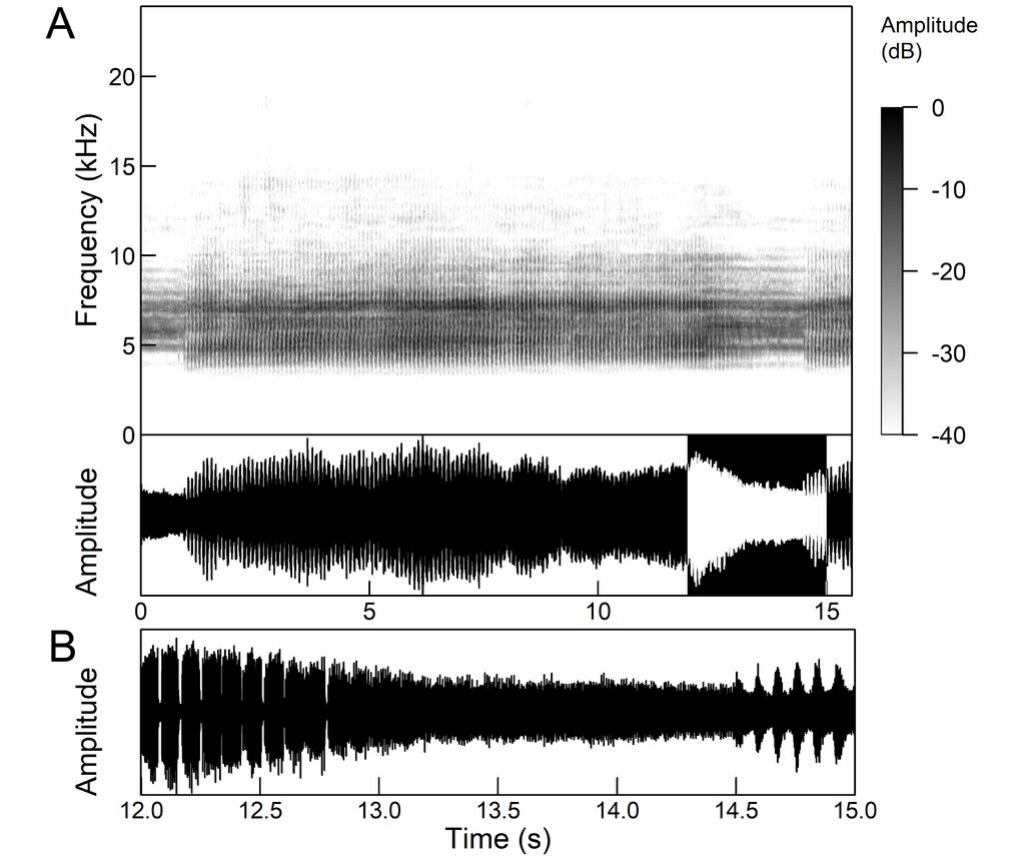
**Calling song of *Lyristes plebejus*.** (A) spectrogram and oscillogram of one phrase of the calling song; (B) oscillogram of the last part of the phrase and beginning of a new one corresponding to the inverted window in (A).

**Figure 12.**
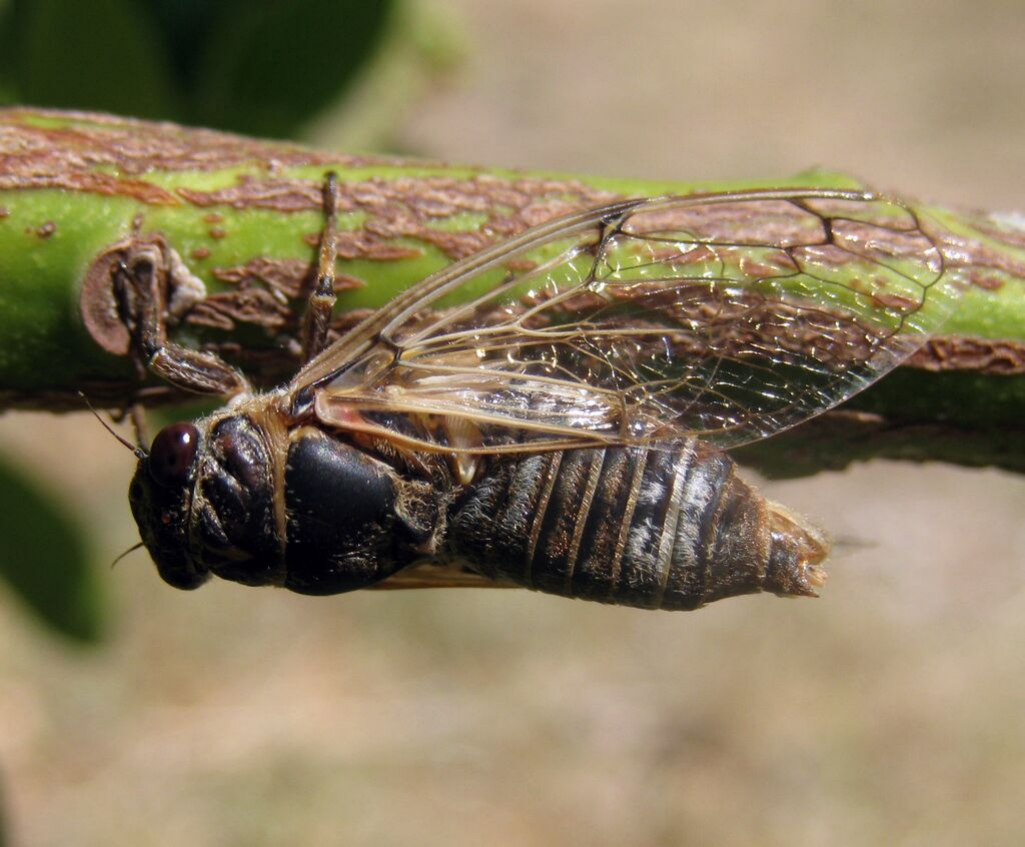
***Cicadatra atra* singing male.**

**Figure 13.**
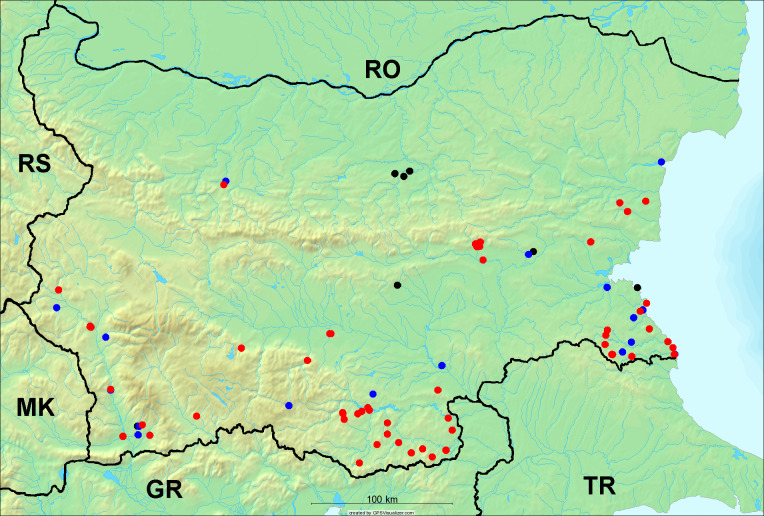
**Map with localities of *Cicadatra atra* in Bulgaria.** Black - literature data, blue - data from collections, red - bioacoustic data collected in this survey.

**Figure 14.**
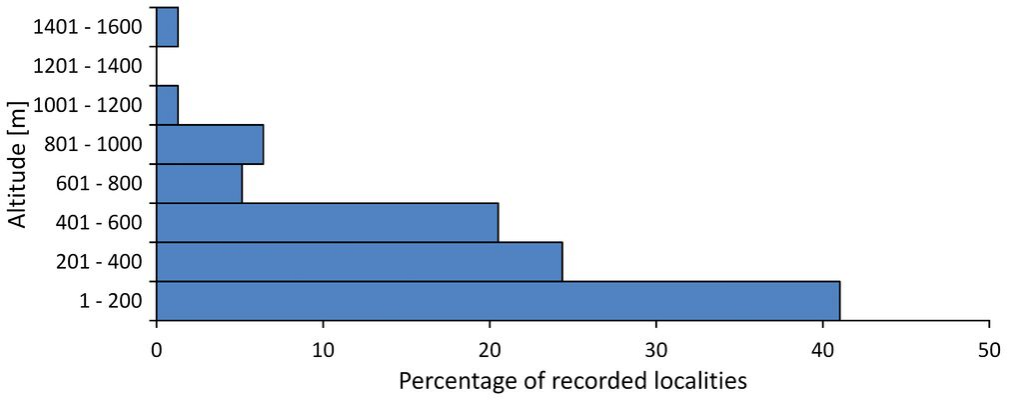
**Altitudinal distribution of *Cicadatra atra*.** The percentage of the recorded localities is displayed in 200-metre altitude zones.

**Figure 15.**
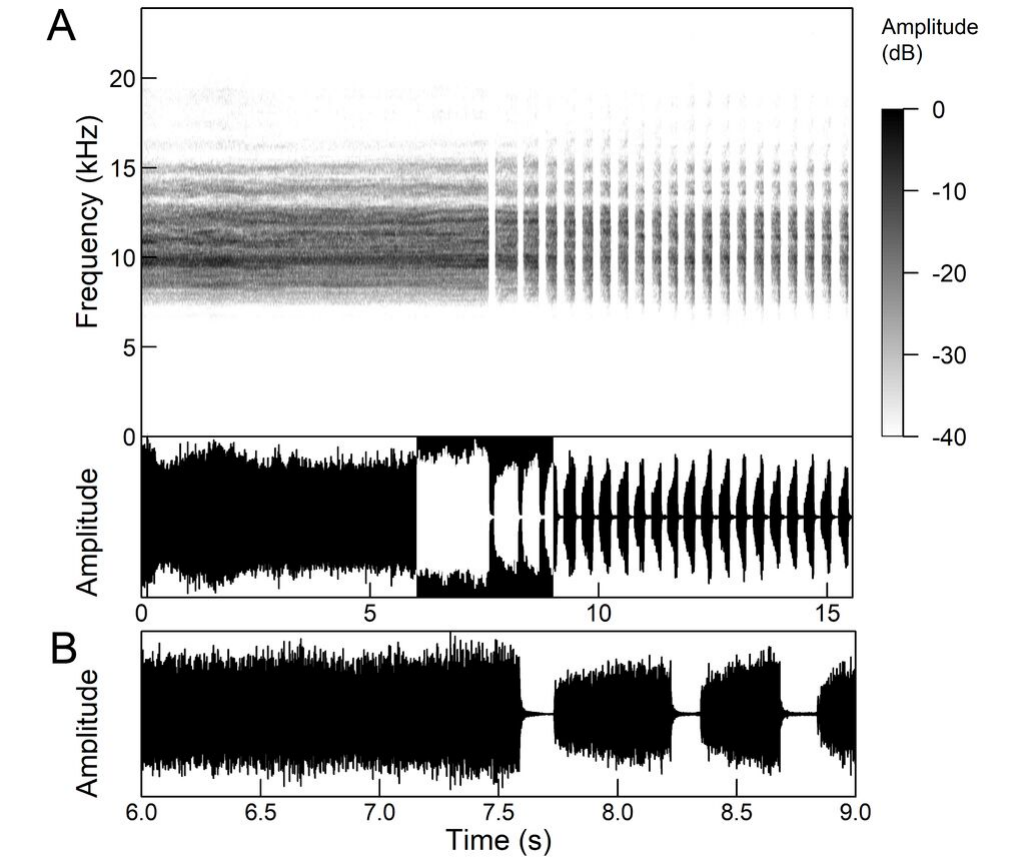
**Continuous and intermittent calling song of *Cicadatra atra*.** (A) spectrogram and oscillogram of the song showing the transition from continuous song to the calling song with short echemes; (B) oscillogram of the enlarged part of the transition from the continuous song to the calling song with short echemes corresponding to the inverted window in (A).

**Figure 16.**
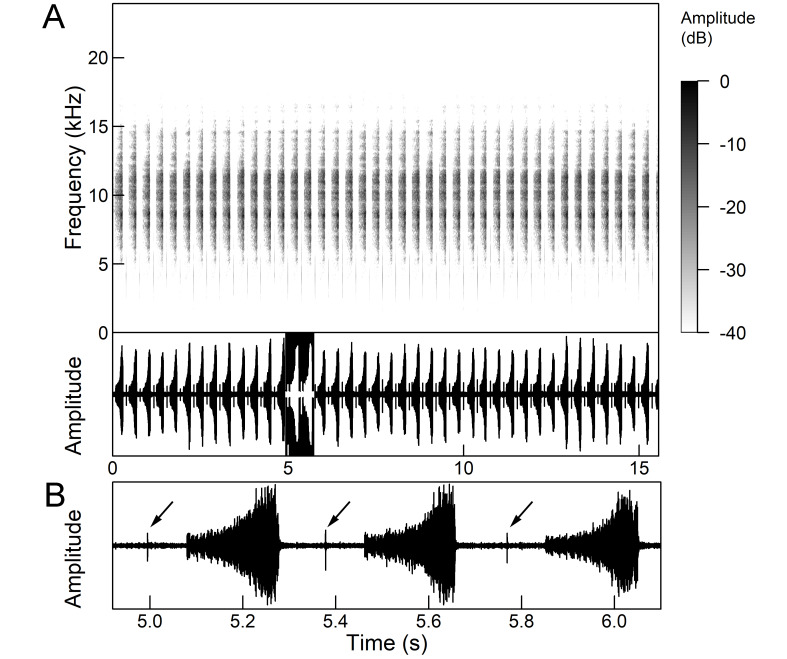
**Courtship song of *Cicadatra atra*.** (A) spectrogram and oscillogram of the courtship song; (B) oscillogram of the enlarged part corresponding to the inverted window in (B), arrows indicate the wing clicks.

**Figure 17.**
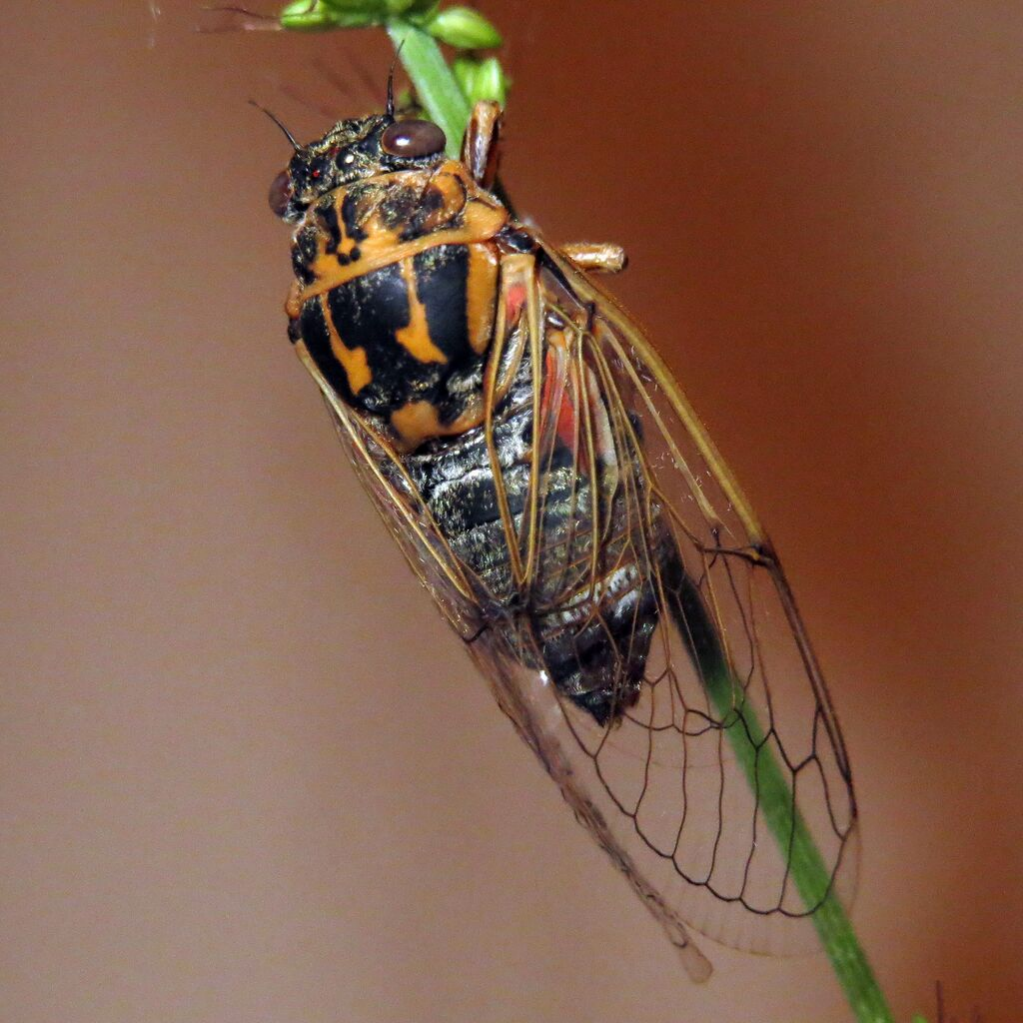
***Cicadatra hyalina* male.**

**Figure 18.**
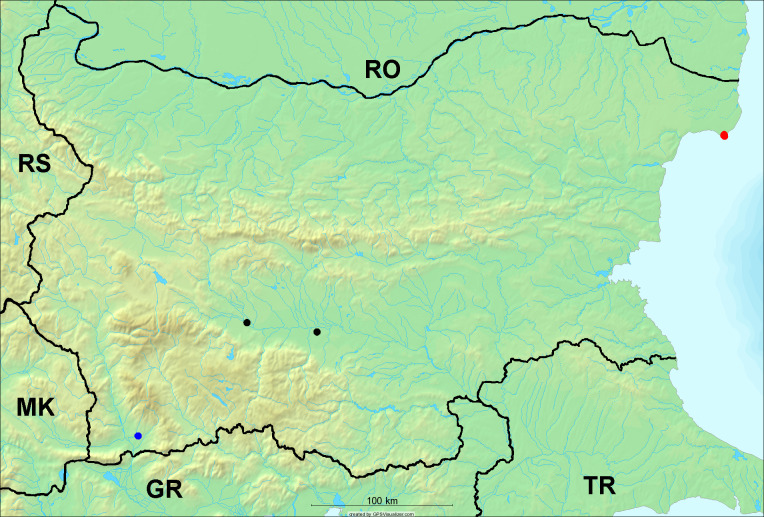
**Map with localities of *Cicadatra hyalina* in Bulgaria.** Black - literature data, blue - data from collections, red - bioacoustic data collected in this survey.

**Figure 19.**
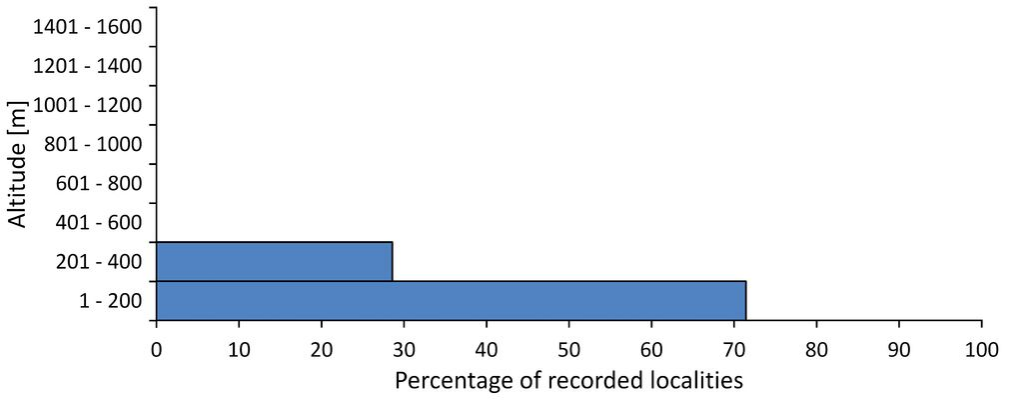
**Altitudinal distribution of *Cicadatra hyalina*.** The percentage of the recorded localities is displayed in 200-metre altitude zones.

**Figure 20.**
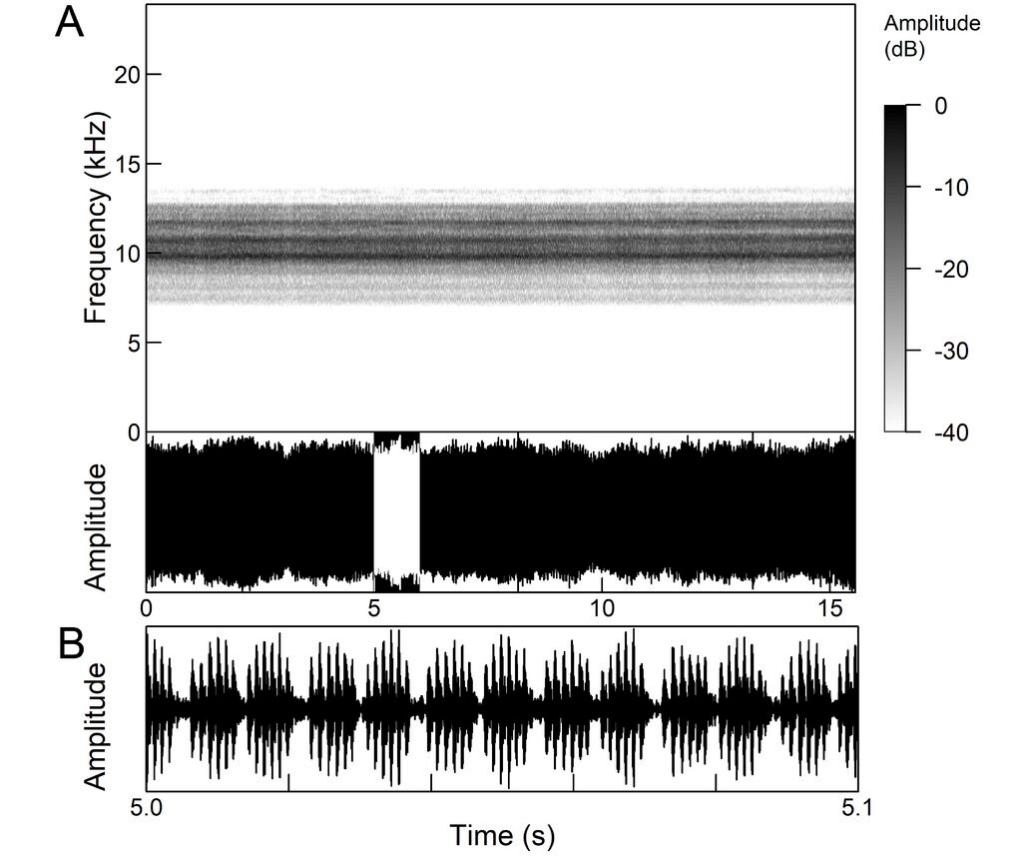
**Continuous calling song of *Cicadatra hyalina*.** (A) spectrogram and oscillogram of the continuous song; (B) oscillogram of the enlarged part corresponding to the inverted window in (A).

**Figure 21.**
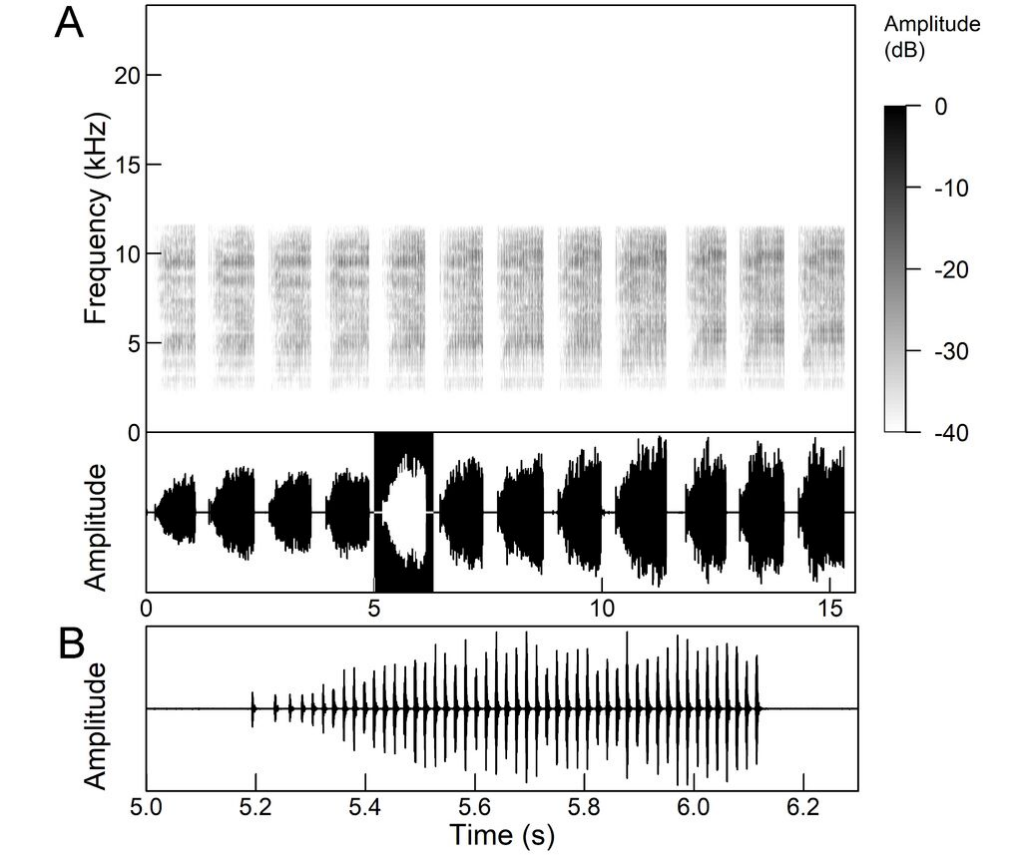
**Intermittent calling song of *Cicadatra hyalina*.** (A) spectrogram and oscillogram of the calling song; (B) oscillogram of one echeme corresponding to the inverted window in (A).

**Figure 22.**
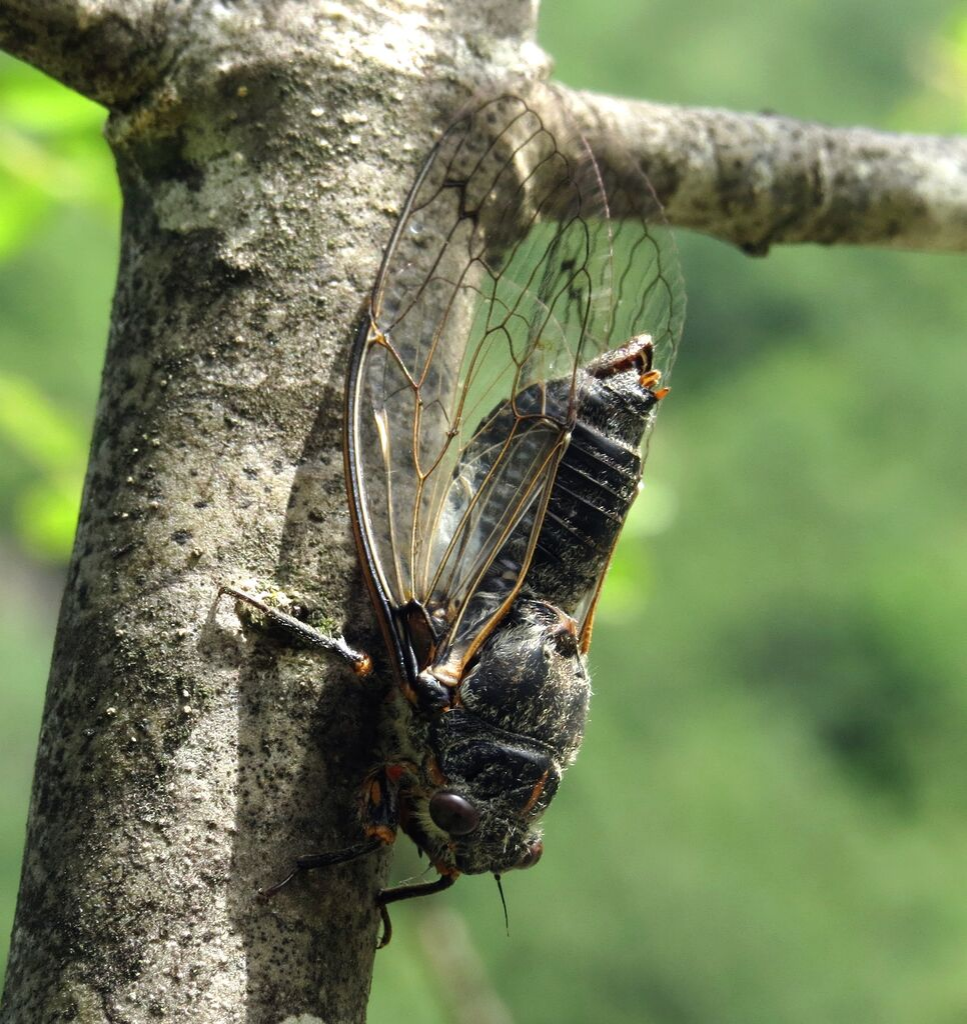
***Cicadatra persica* singing male.**

**Figure 23.**
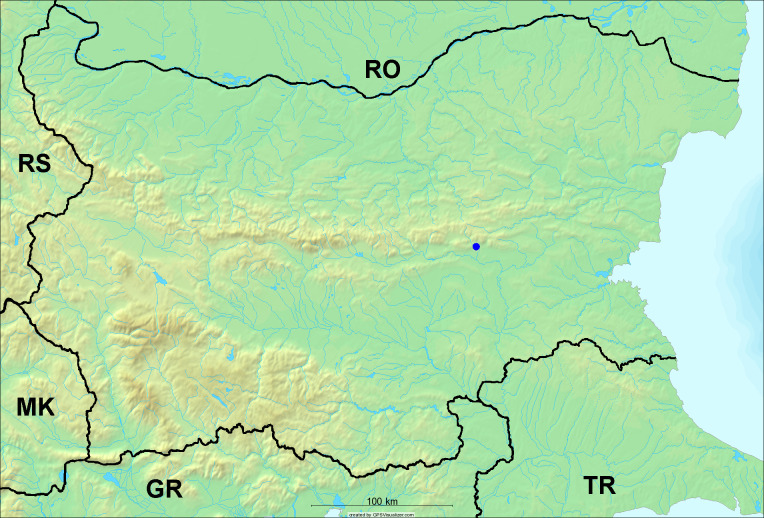
**Map with localities of *Cicadatra persica* in Bulgaria.** Blue - data from collections.

**Figure 24.**
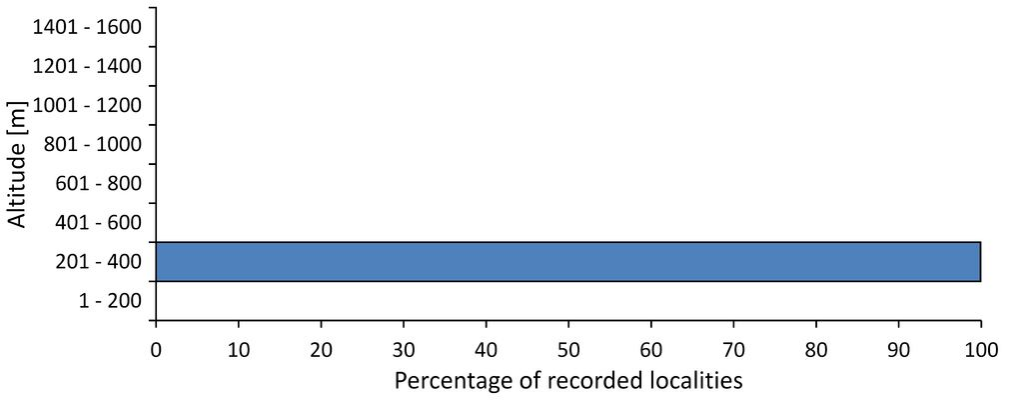
**Altitudinal distribution of *Cicadatra persica*.** The percentage of the recorded localities is displayed in 200-metre altitude zones.

**Figure 25.**
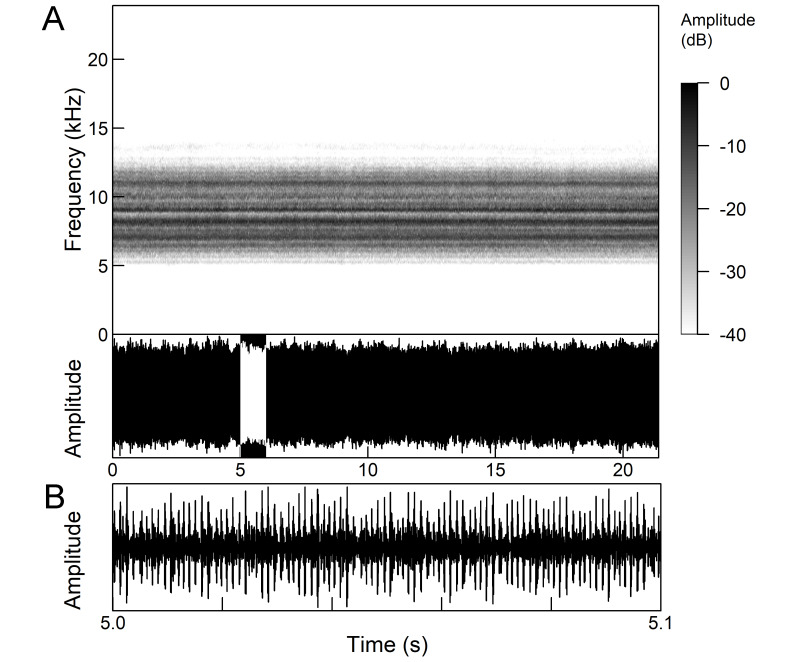
**Calling song of *Cicadatra persica*.** (A) spectrogram and oscillogram of the selected part of the continuous calling song; (B) oscillogram of the enlarged part corresponding to the inverted window in (A).

**Figure 26.**
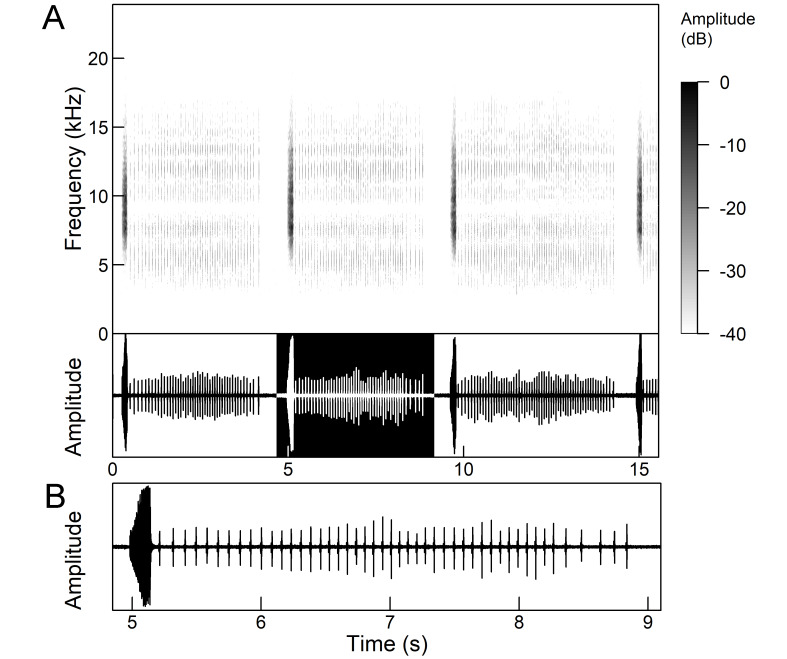
**Courtship song of *Cicadatra persica*.** (A) spectrogram and oscillogram of the selected part of the courtship song accompanied by wing clicks; (B) oscillogram of the enlarged part of the courtship song accompanied by wing clicks corresponding to the inverted window in (A).

**Figure 27.**
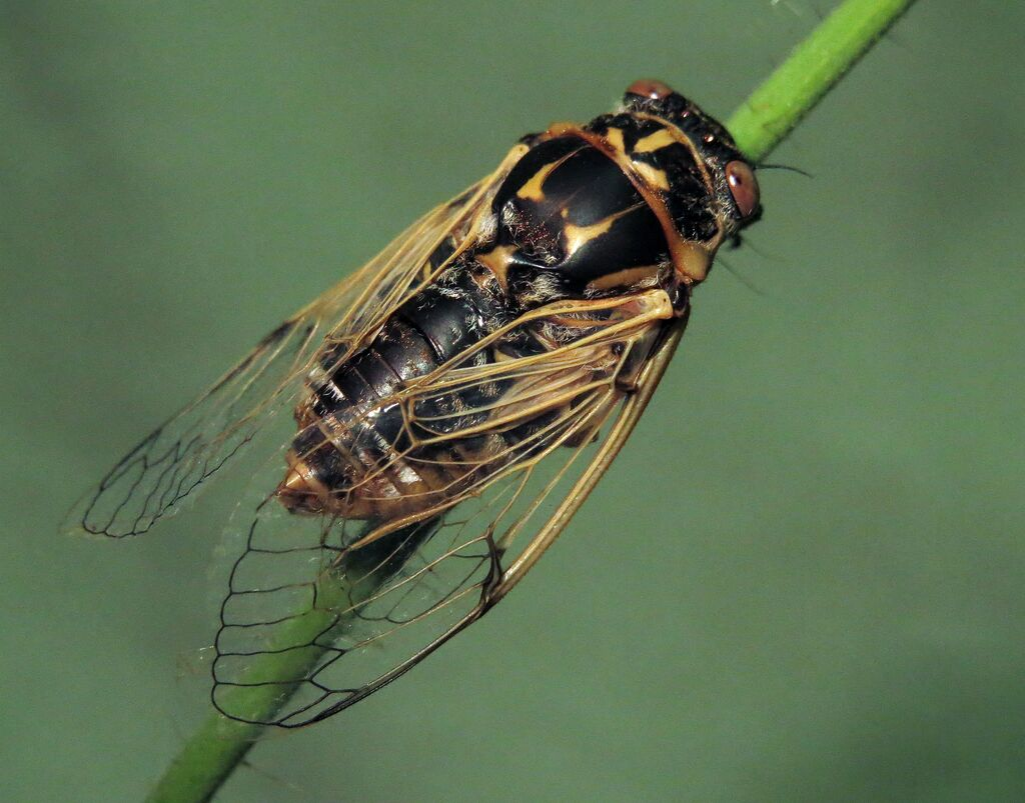
***Cicadatra platyptera* male.**

**Figure 28.**
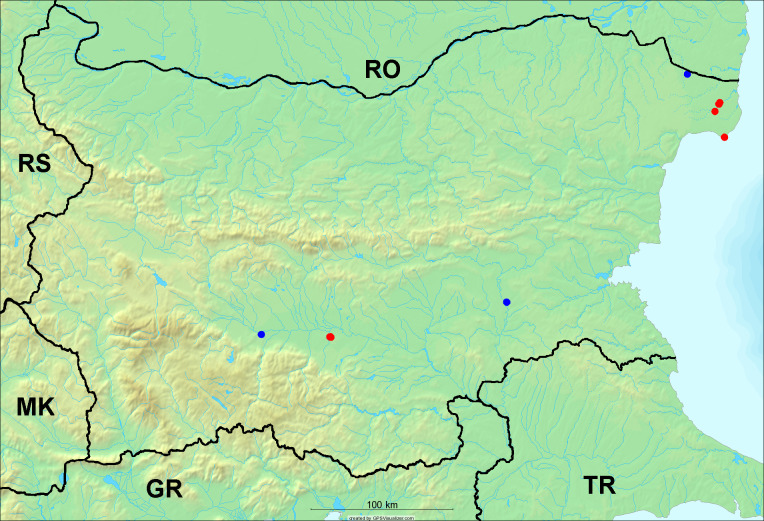
**Map with localities of *Cicadatra platyptera* in Bulgaria.** Blue - data from collections, red - bioacoustic data collected in this survey.

**Figure 29.**
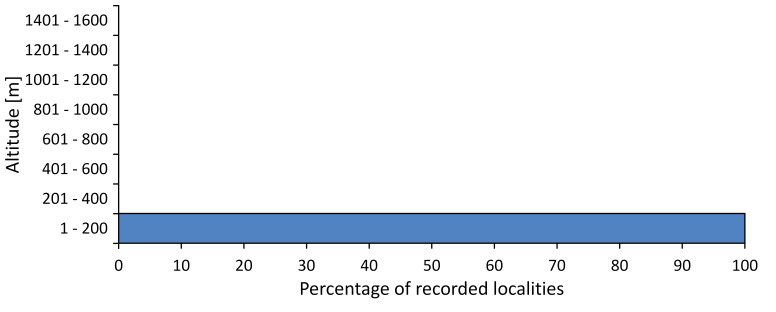
**Altitudinal distribution of *Cicadatra platyptera*.** The percentage of the recorded localities is displayed in 200-metre altitude zones.

**Figure 30.**
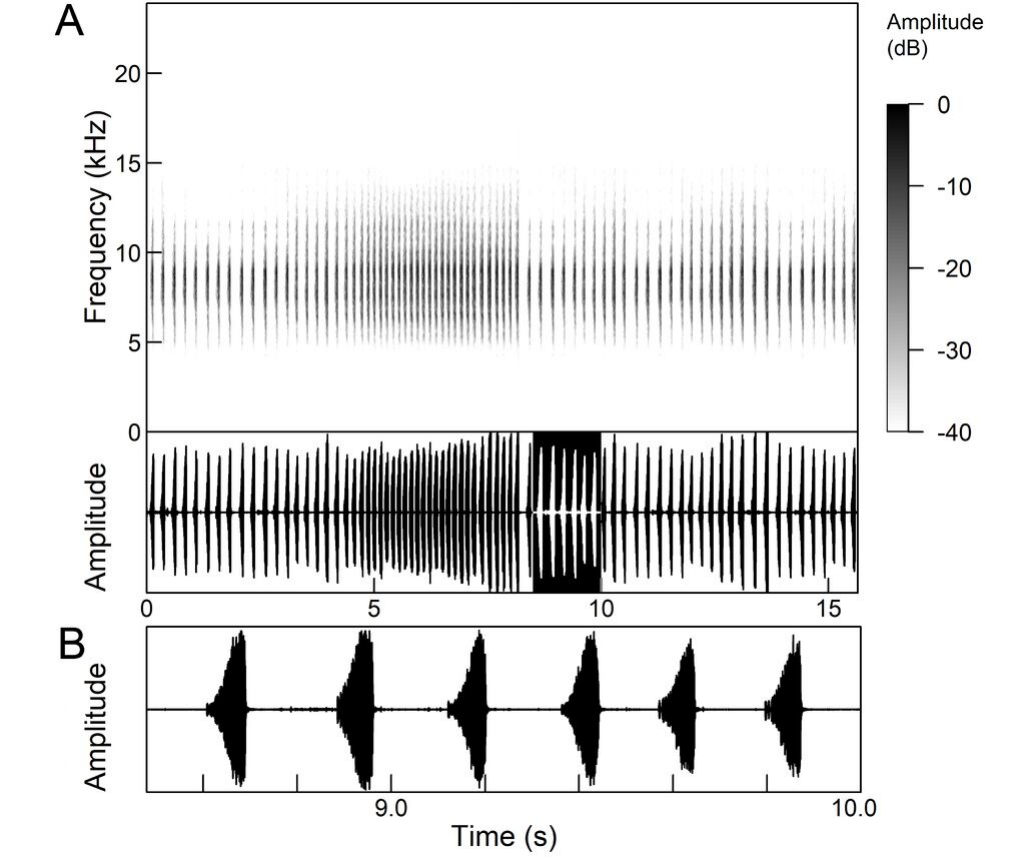
**Calling song of *Cicadatra platyptera*.** (A) spectrogram and oscillogram of the calling song; (B) oscillogram of six echemes corresponding to the inverted window in (A).

**Figure 31.**
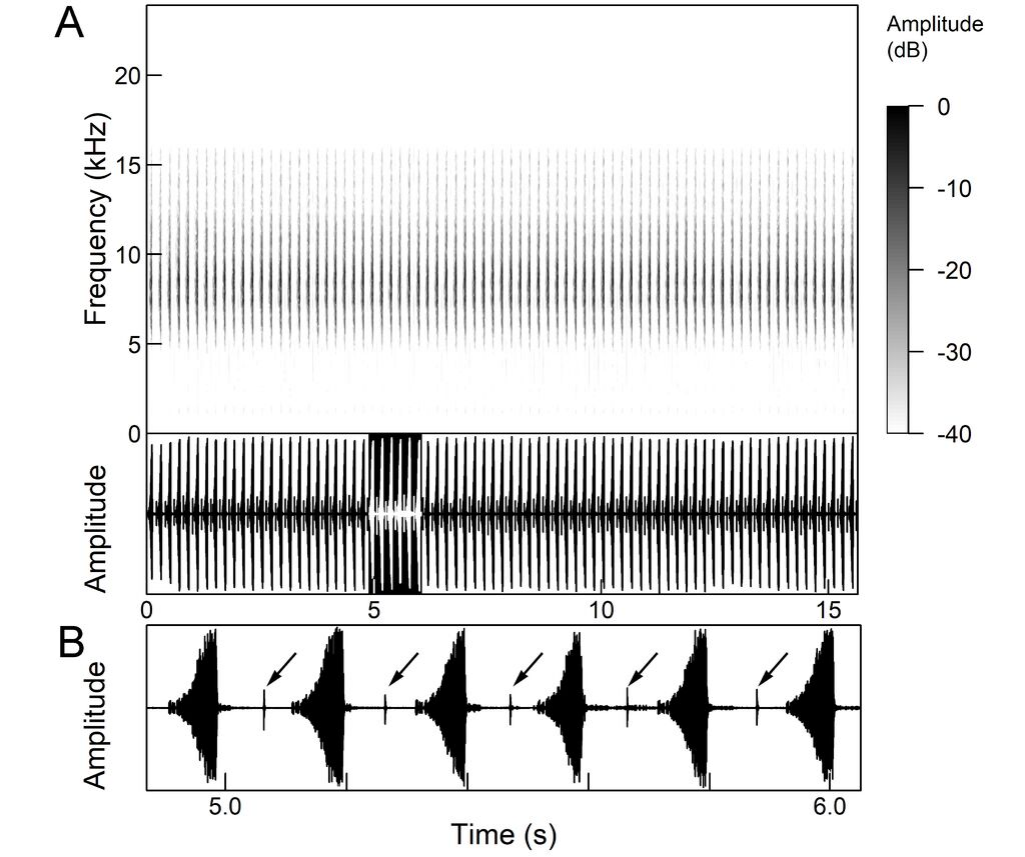
**Courtship song of *Cicadatra platyptera*.** (A) spectrogram and oscillogram of the courtship song; (B) oscillogram of the enlarged part corresponding to the inverted window in (B), with arrows indicating wing clicks.

**Figure 32.**
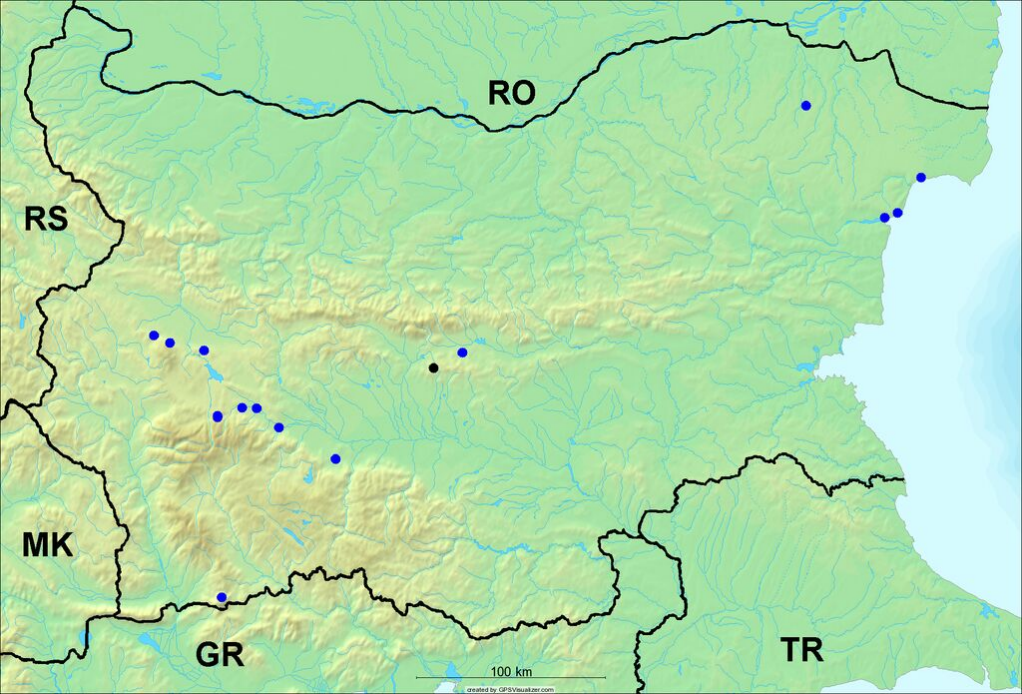
**Map with localities of *Cicadetta montana* s. lat. in Bulgaria.** Black - literature data, blue - data from collections.

**Figure 33.**
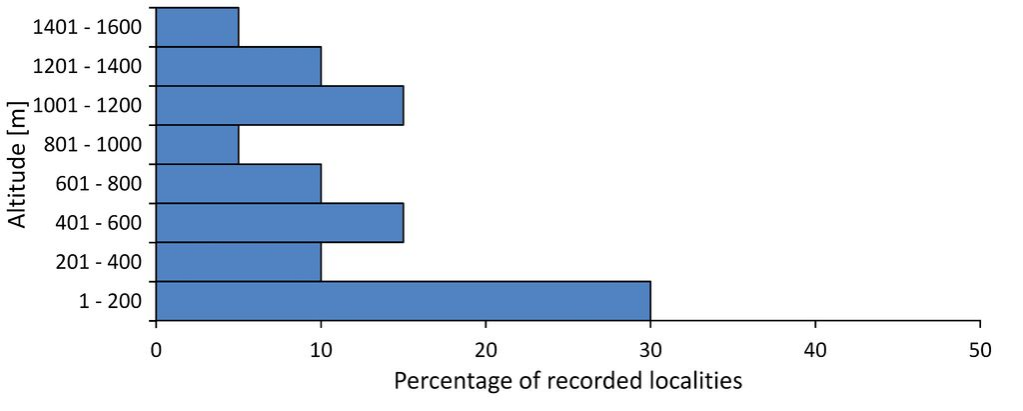
**Altitudinal distribution of *Cicadetta montana* s. lat.** The percentage of the recorded localities is displayed in 200-metre altitude zones.

**Figure 34.**
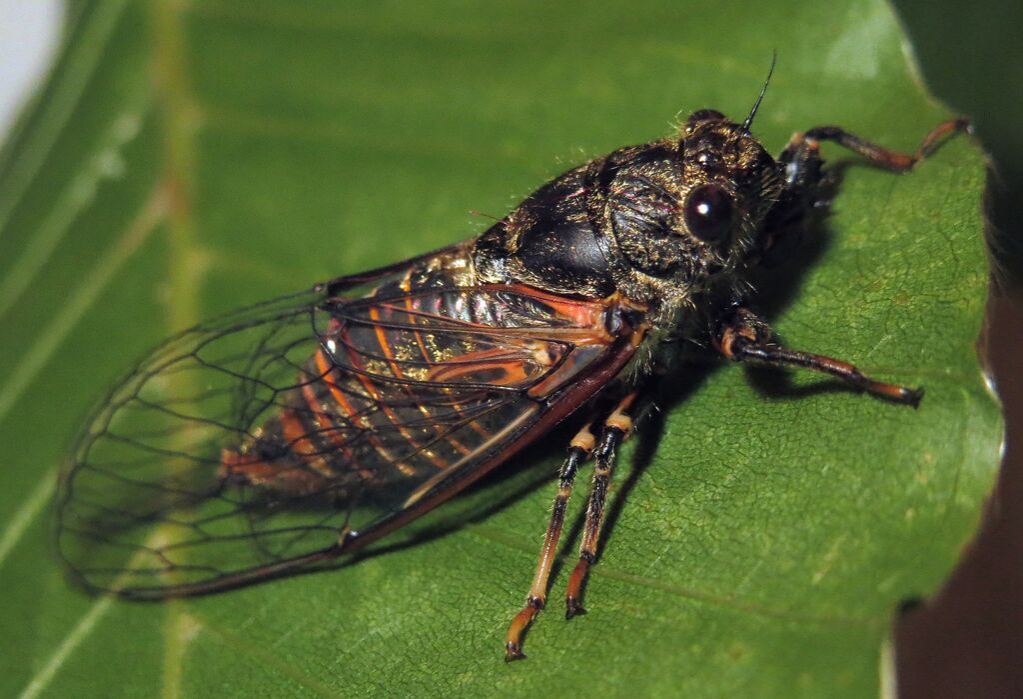
***Cicadetta montana* s. str. female.**

**Figure 35.**
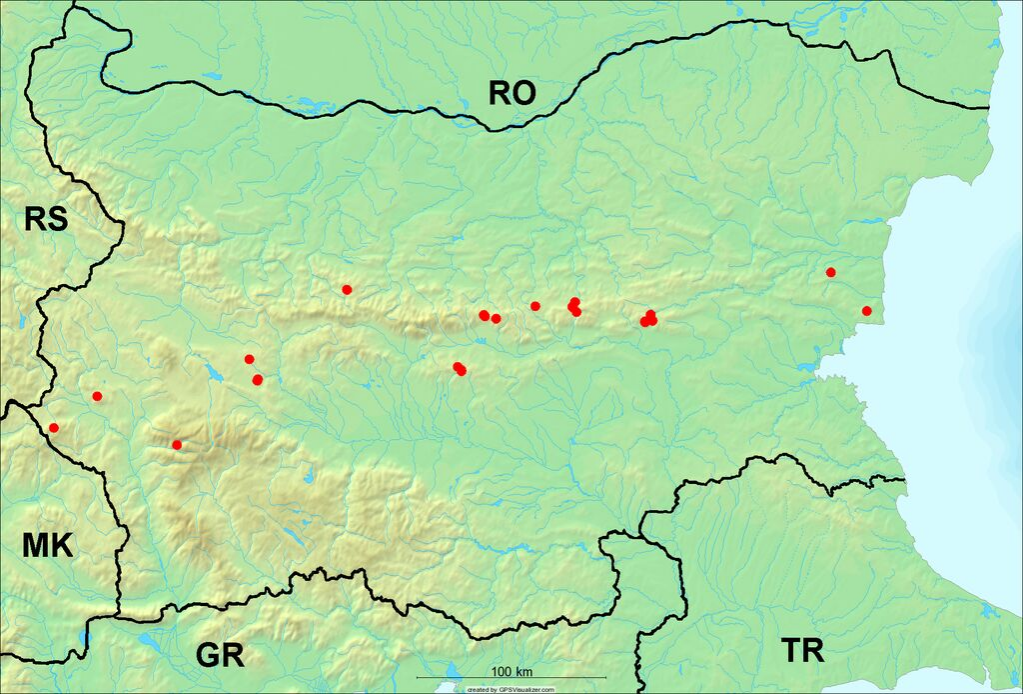
**Map with localities of *Cicadetta montana* s. str. in Bulgaria.** Red - bioacoustic data collected in this survey.

**Figure 36.**
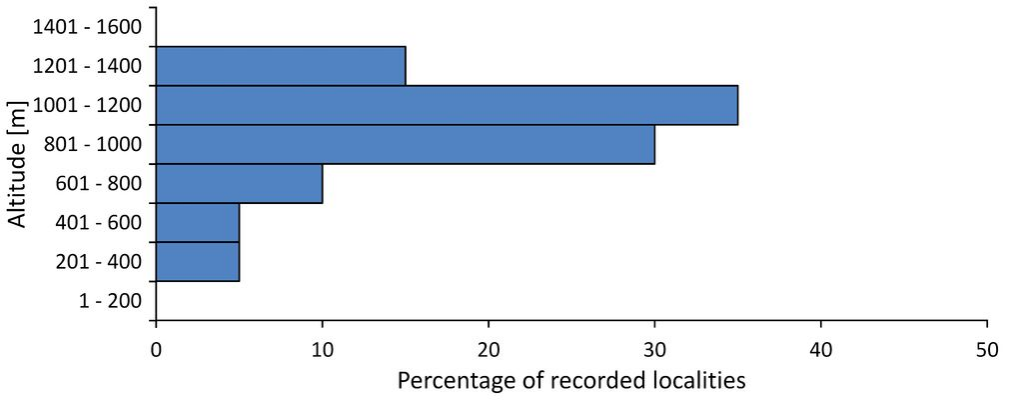
**Altitudinal distribution of *Cicadetta montana* s. str.** The percentage of the recorded localities is displayed in 200-metre altitude zones.

**Figure 37.**
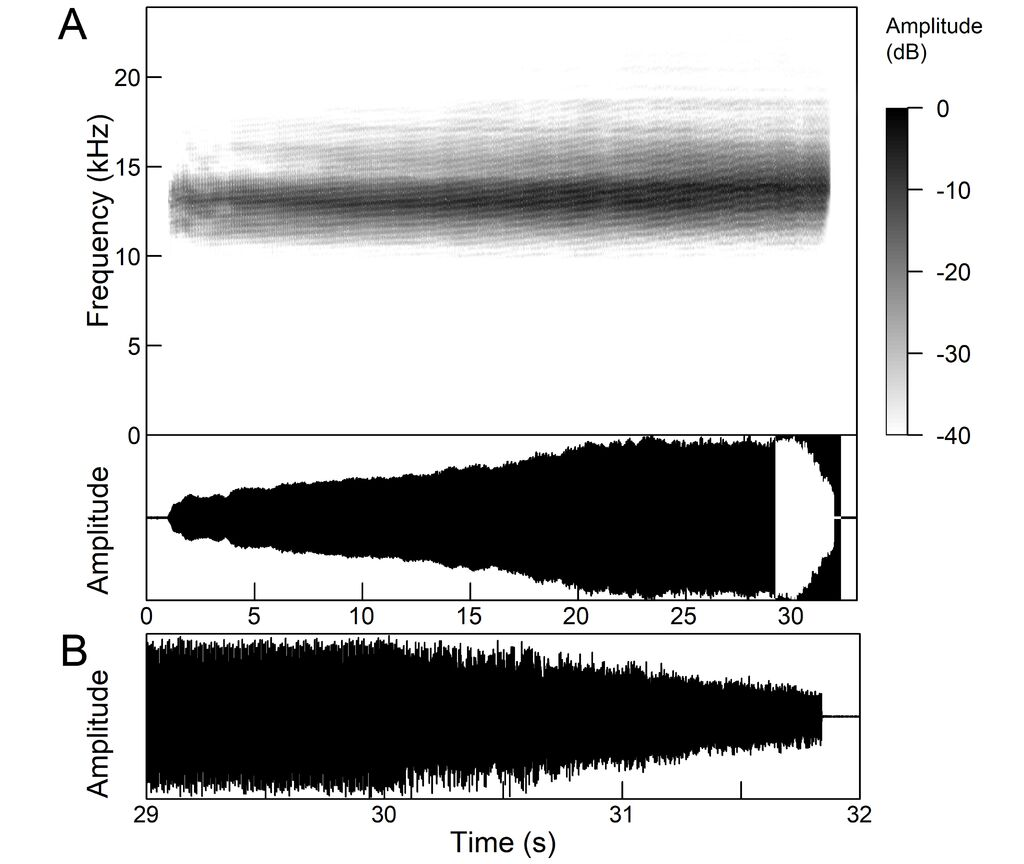
**Calling song of *Cicadetta montana* s. str.** (A) spectrogram and oscillogram of the calling song; (B) oscillogram of the end of the calling song corresponding to the inverted window in (A).

**Figure 38.**
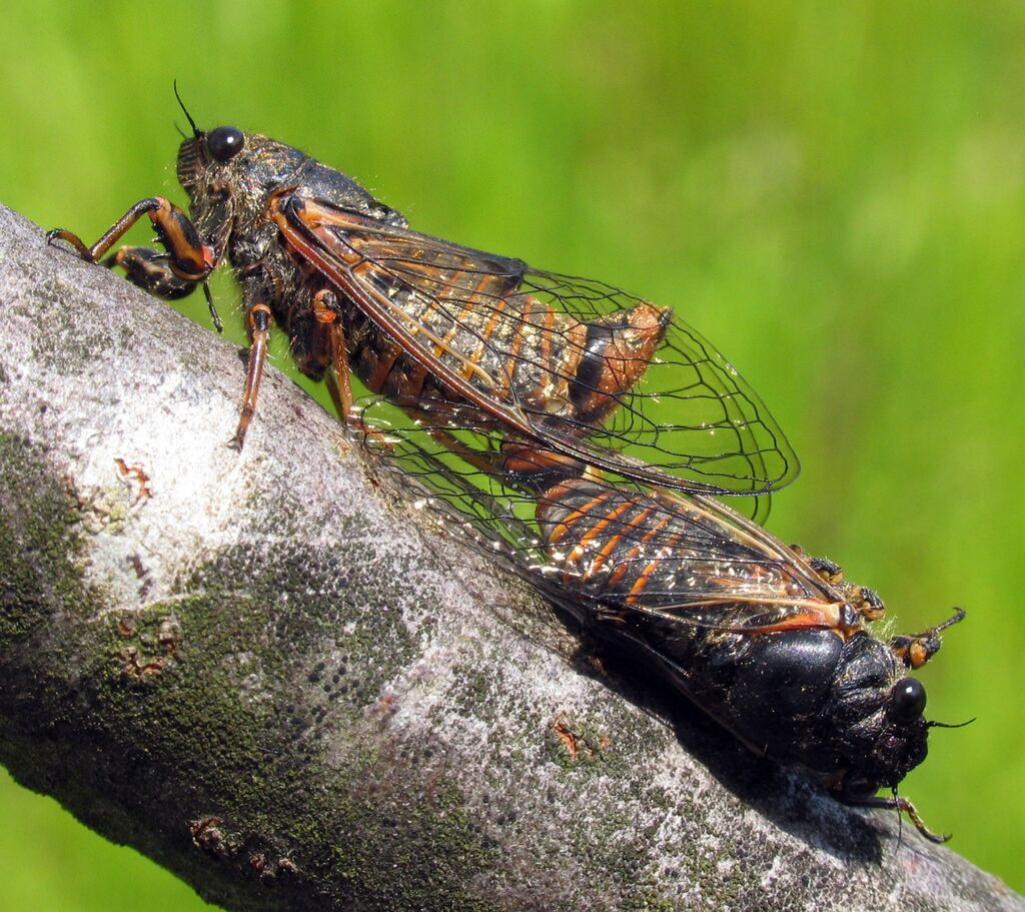
***Cicadetta brevipennis* s. lat. male and female in copula.**

**Figure 39.**
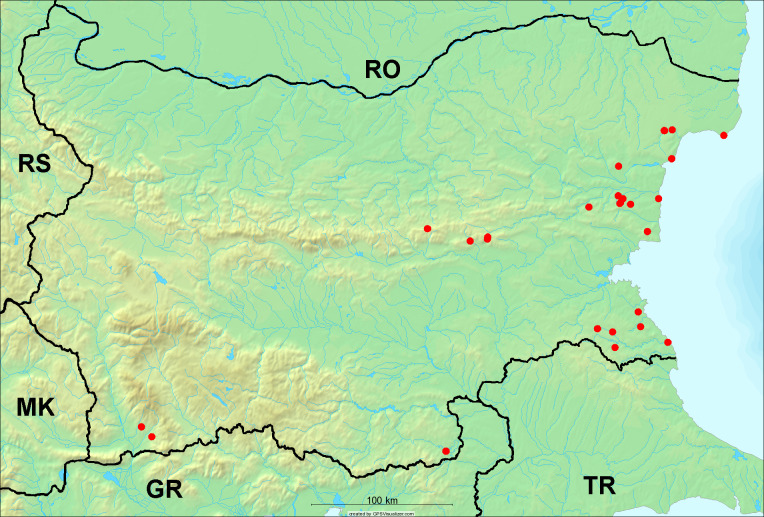
**Map with localities of *Cicadetta brevipennis* s. lat. in Bulgaria.** Red - bioacoustic data collected in this survey.

**Figure 40.**
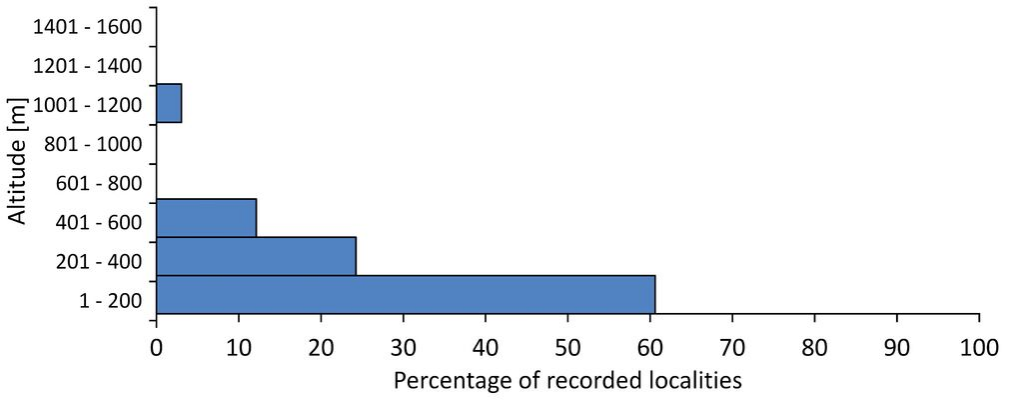
**Altitudinal distribution of *Cicadetta brevipennis* s. lat..** The percentage of the recorded localities is displayed in 200-metre altitude zones.

**Figure 41.**
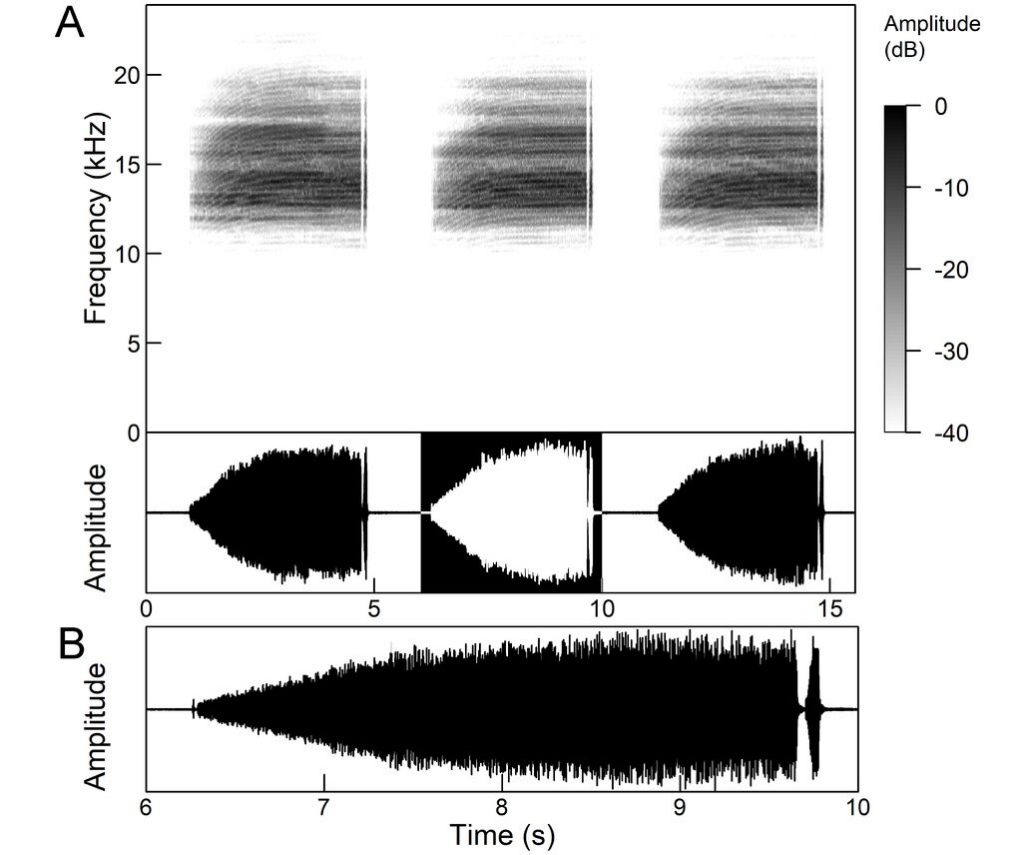
**Calling song of *Cicadetta brevipennis* s. lat..** (A) spectrogram and oscillogram of the three sequences of the long echeme followed by the short echeme of the calling song; (B) oscillogram of the long echeme followed by the short echeme corresponding to the inverted window in (A).

**Figure 42.**
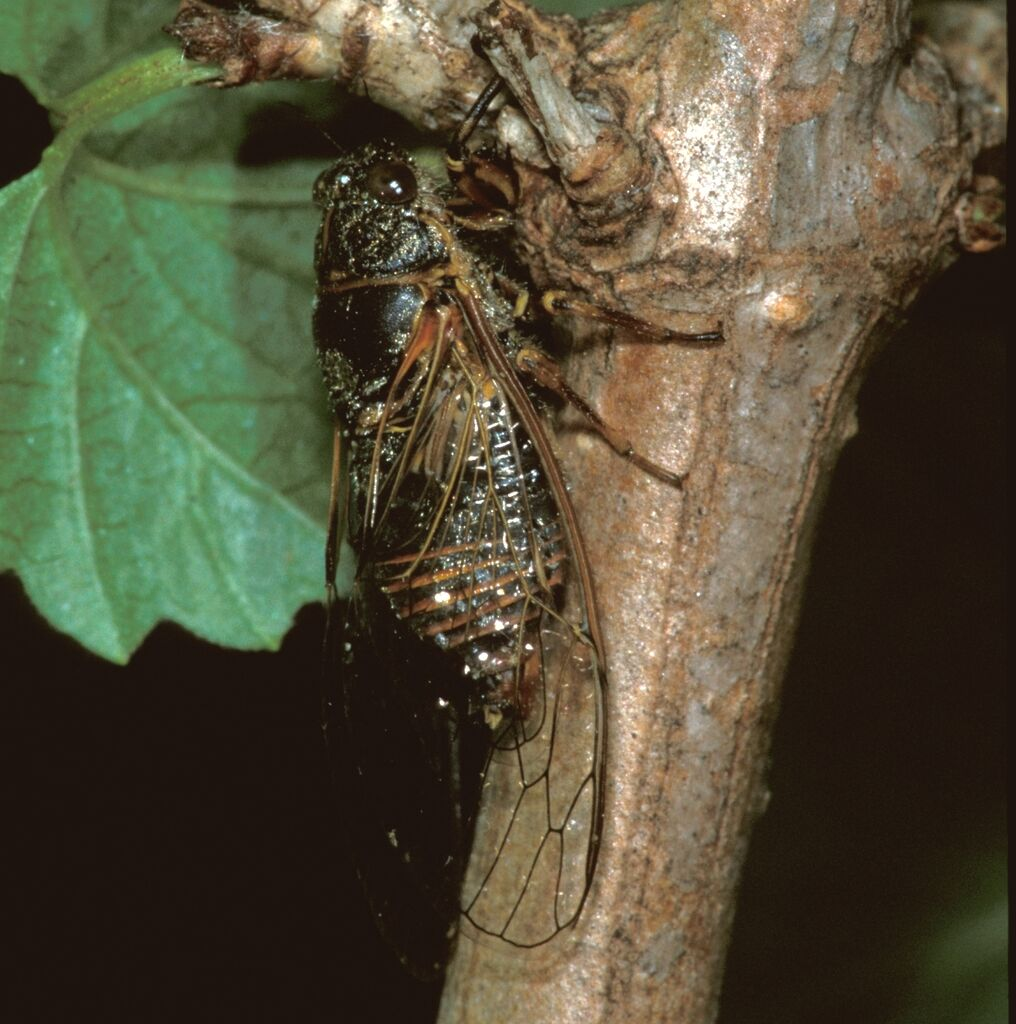
***Cicadetta cantilatrix* male.**

**Figure 43.**
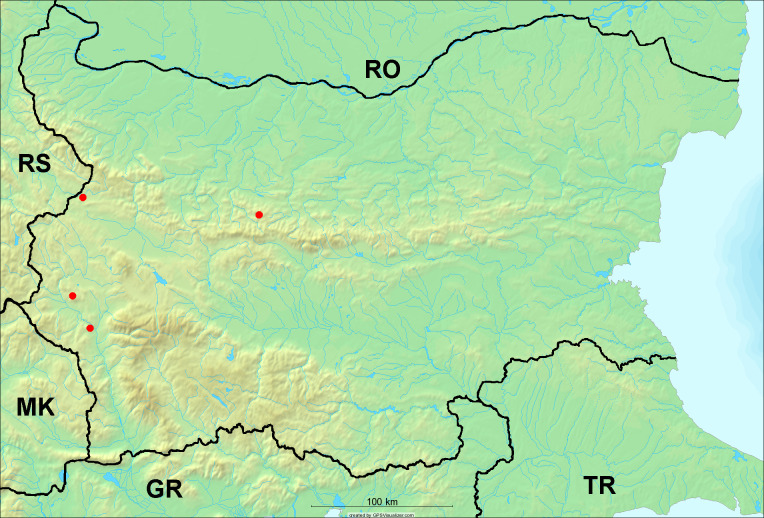
**Map with localities of *Cicadetta cantilatrix* in Bulgaria.** Red - bioacoustic data collected in this survey.

**Figure 44.**
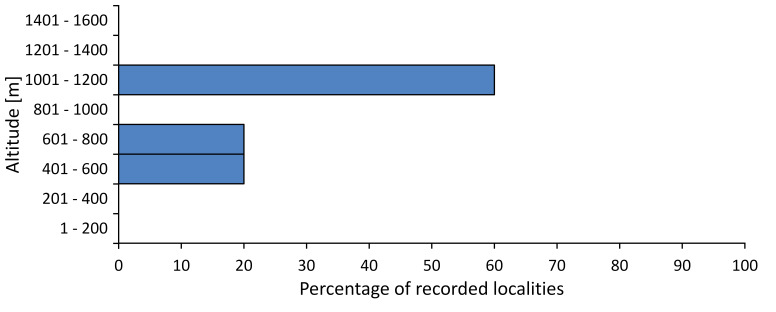
**Altitudinal distribution of *Cicadetta cantilatrix*.** The percentage of the recorded localities is displayed in 200-metre altitude zones.

**Figure 45.**
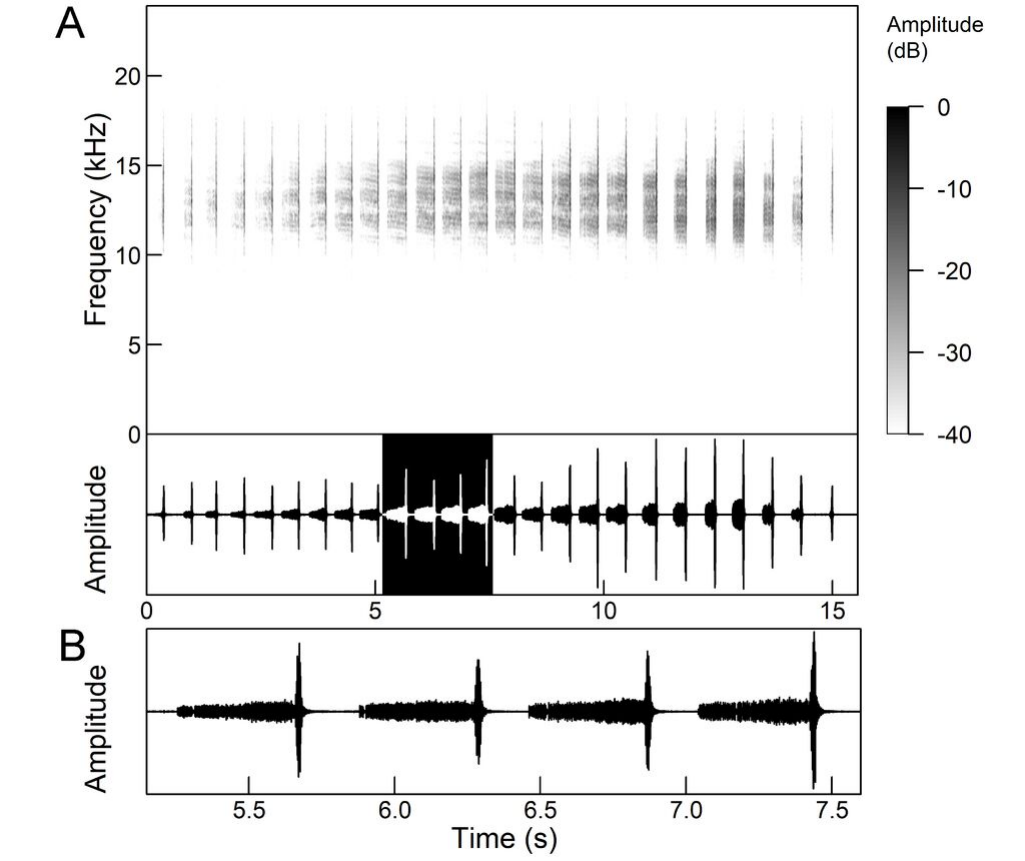
**Calling song of *Cicadetta cantilatrix*.** (A) spectrogram and oscillogram of the selected part of the calling song; (B) oscillogram of four two-phase echemes corresponding to the inverted window in (A).

**Figure 46.**
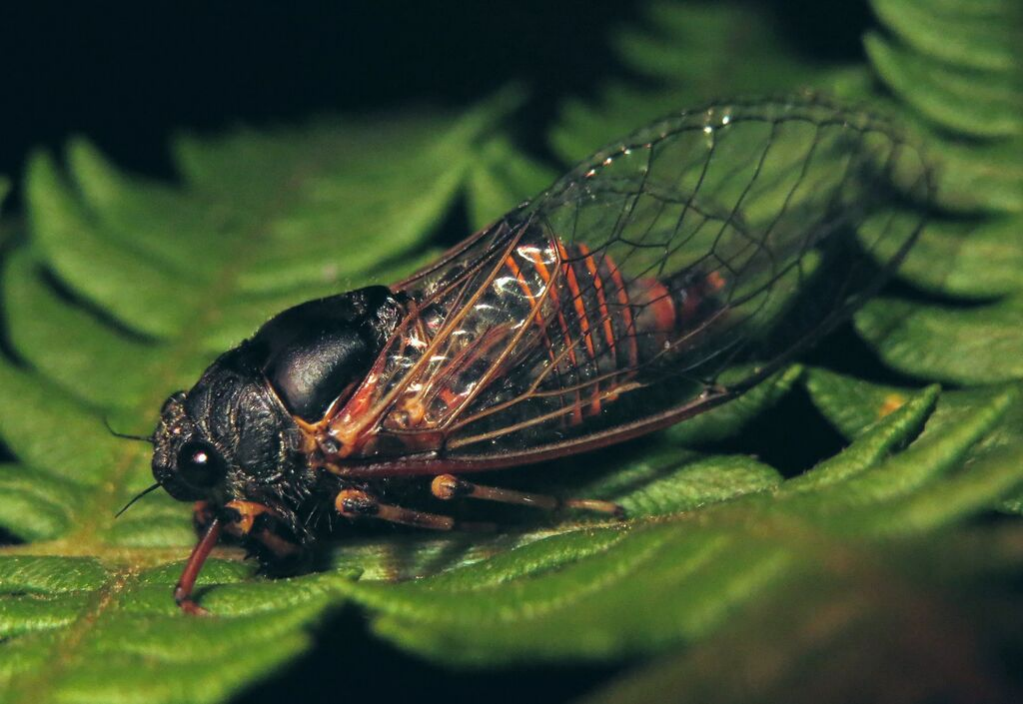
***Cicadetta macedonica* male.**

**Figure 47.**
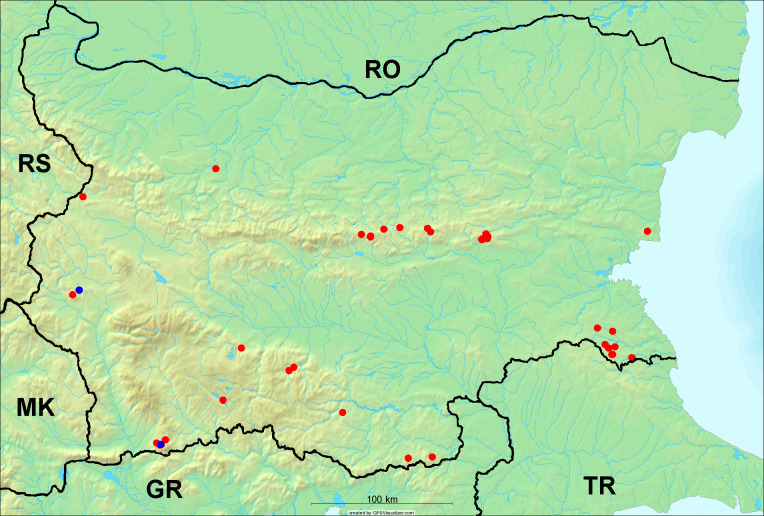
**Map with localities of *Cicadetta macedonica* in Bulgaria.** Blue - data from collections, red - bioacoustic data collected in this survey.

**Figure 48.**
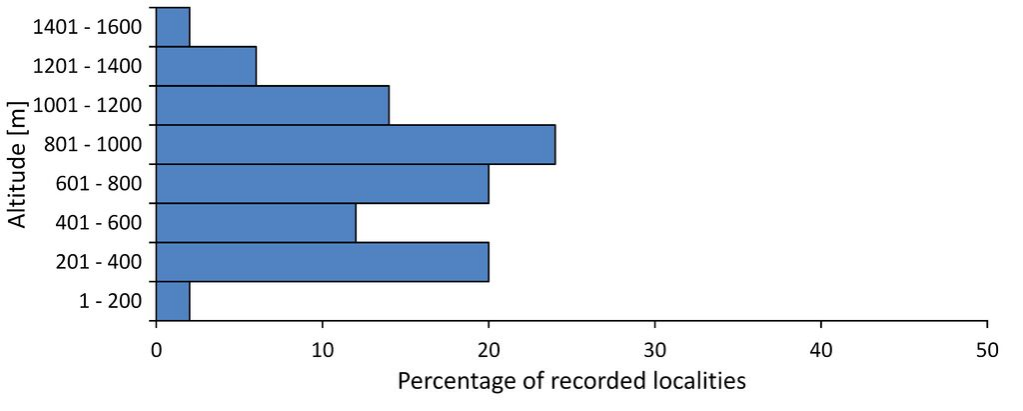
**Altitudinal distribution of *Cicadetta macedonica*.** The percentage of the recorded localities is displayed in 200-metre altitude zones.

**Figure 49.**
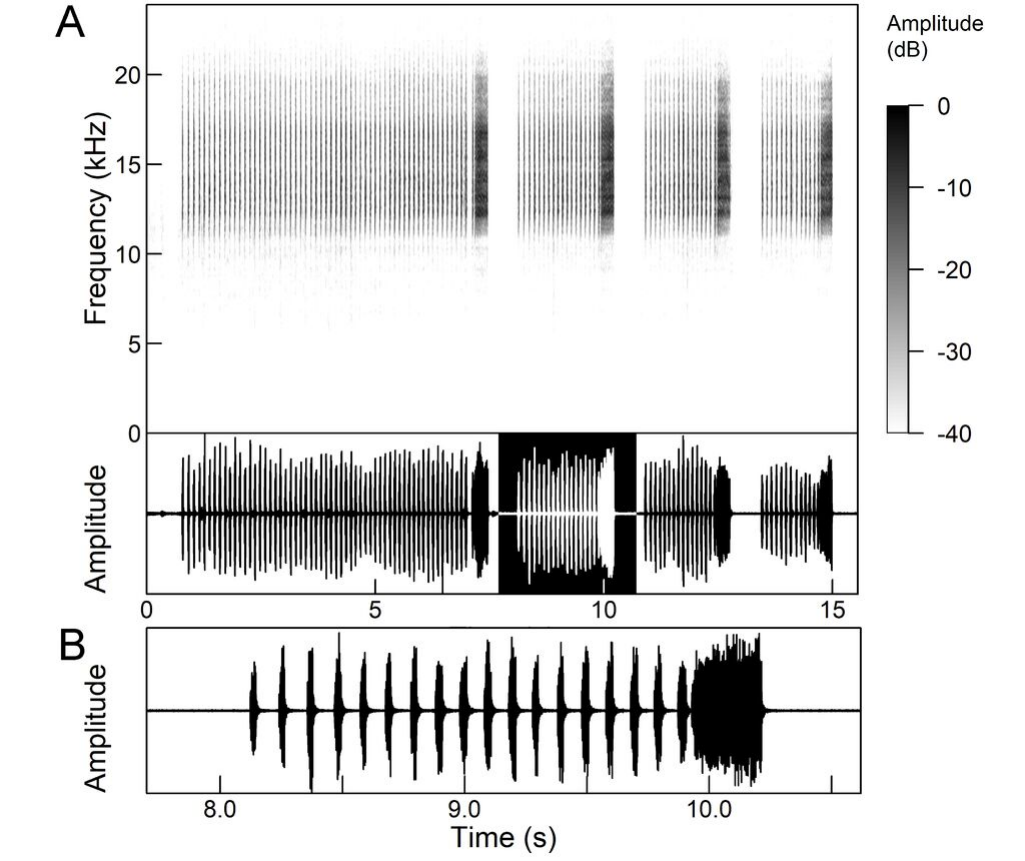
**Calling song of *Cicadetta macedonica*.** (A) spectrogram and oscillogram of four phrases of the calling song; (B) oscillogram of one phrase corresponding to the inverted window in (A).

**Figure 50.**
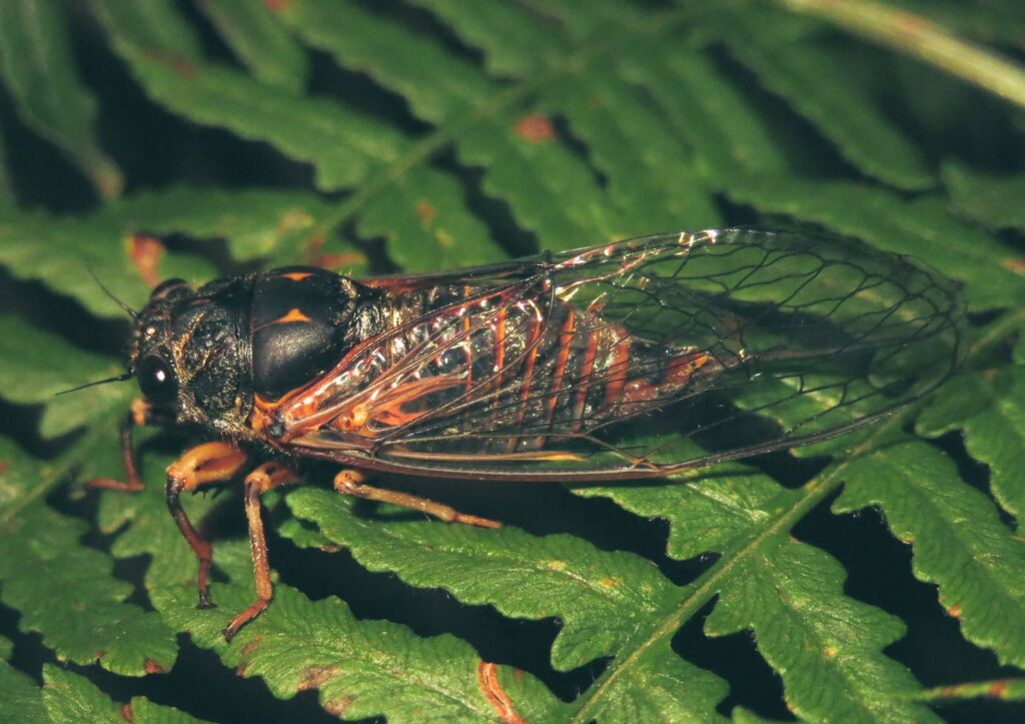
***Dimissalna dimissa* male.**

**Figure 51.**
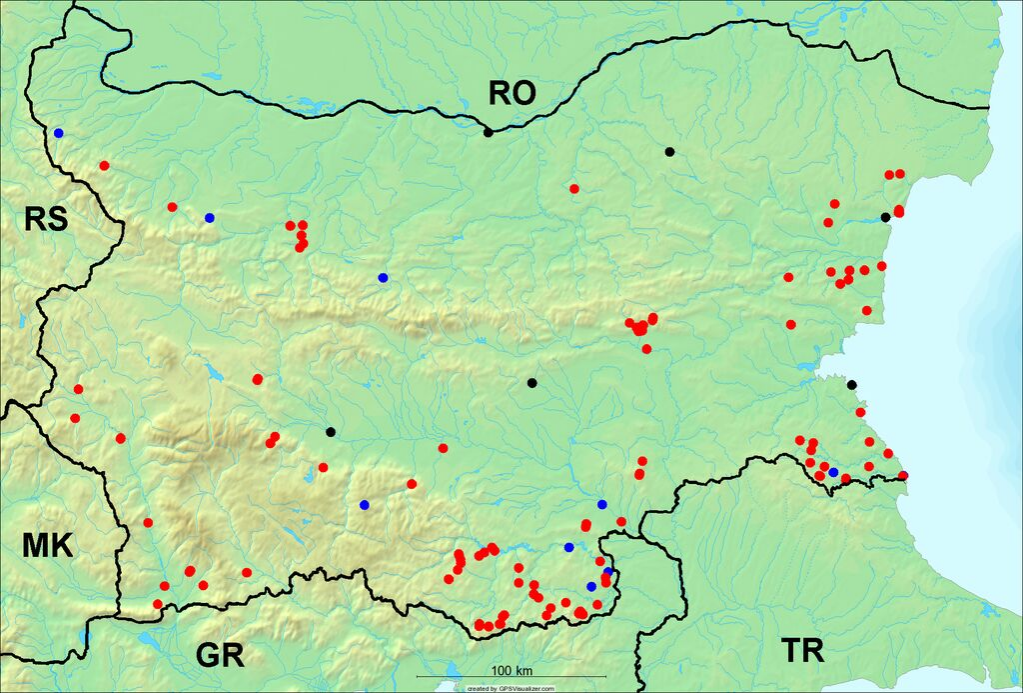
**Map with localities of *Dimissalna dimissa* in Bulgaria.** Black - literature data, blue - data from collections, red - bioacoustic data collected in this survey.

**Figure 52.**
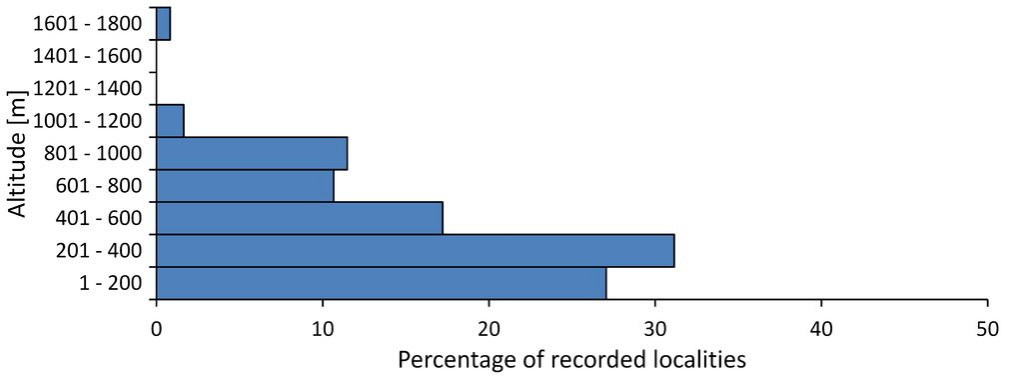
**Altitudinal distribution of *Dimissalna dimissa*.** The percentage of the recorded localities is displayed in 200-metre altitude zones.

**Figure 53.**
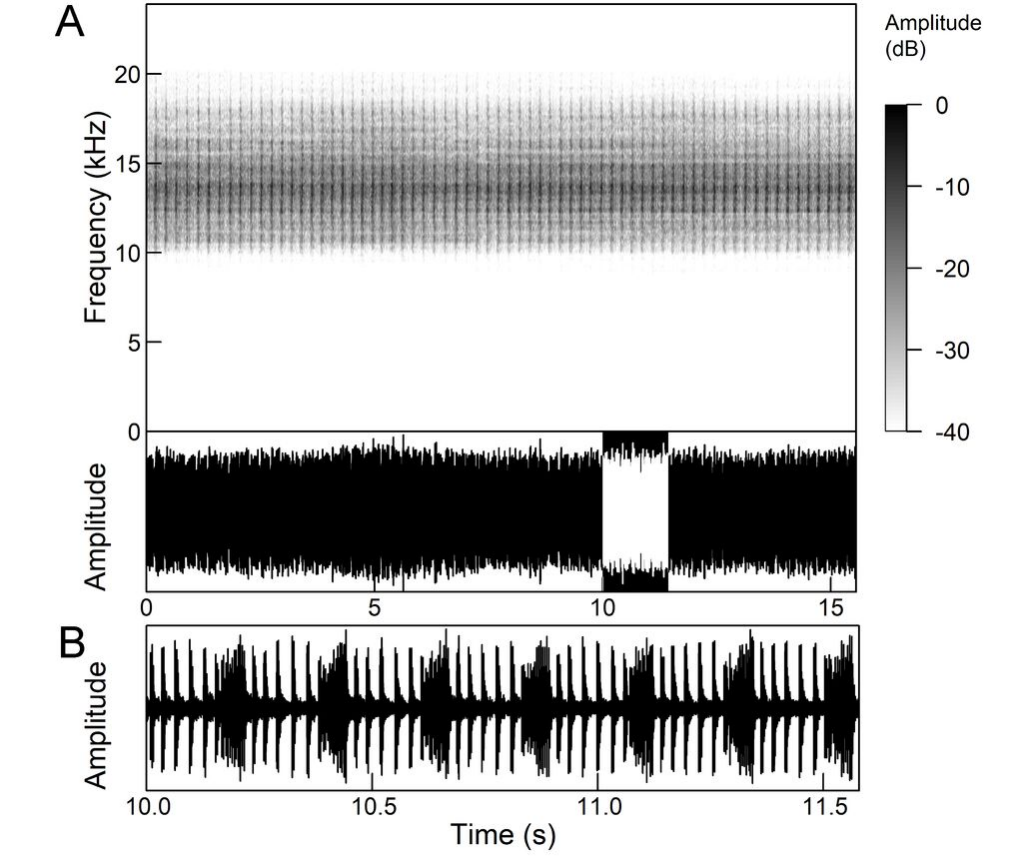
**First type of song of *Dimissalna dimissa*.** (A) spectrogram and oscillogram of the selected part; (B) oscillogram of the enlarged part corresponding to the inverted window in (A).

**Figure 54.**
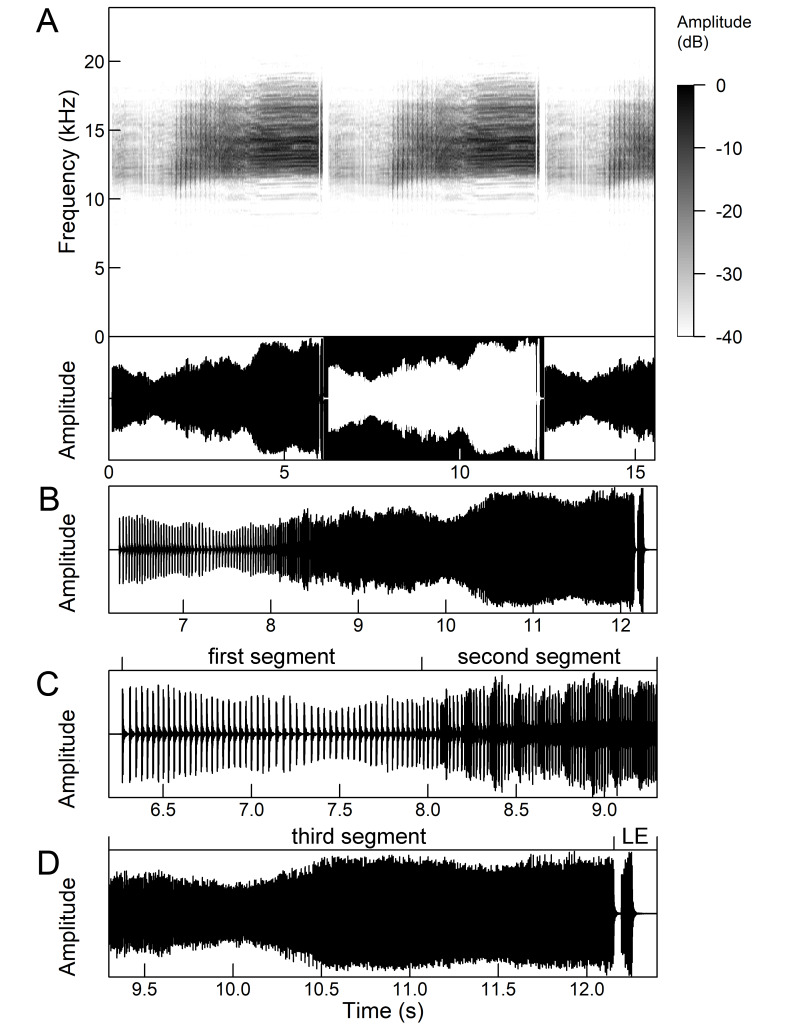
**Second type of song of *Dimissalna dimissa*.** (A) spectrogram and oscillogram of the second type of song; (B) oscillogram of one sequence of the second type of song corresponding to the inverse window in (A); (C) oscillogram of the first and second segments; (D) oscillogram of the third segment and final long echeme (LE).

**Figure 55.**
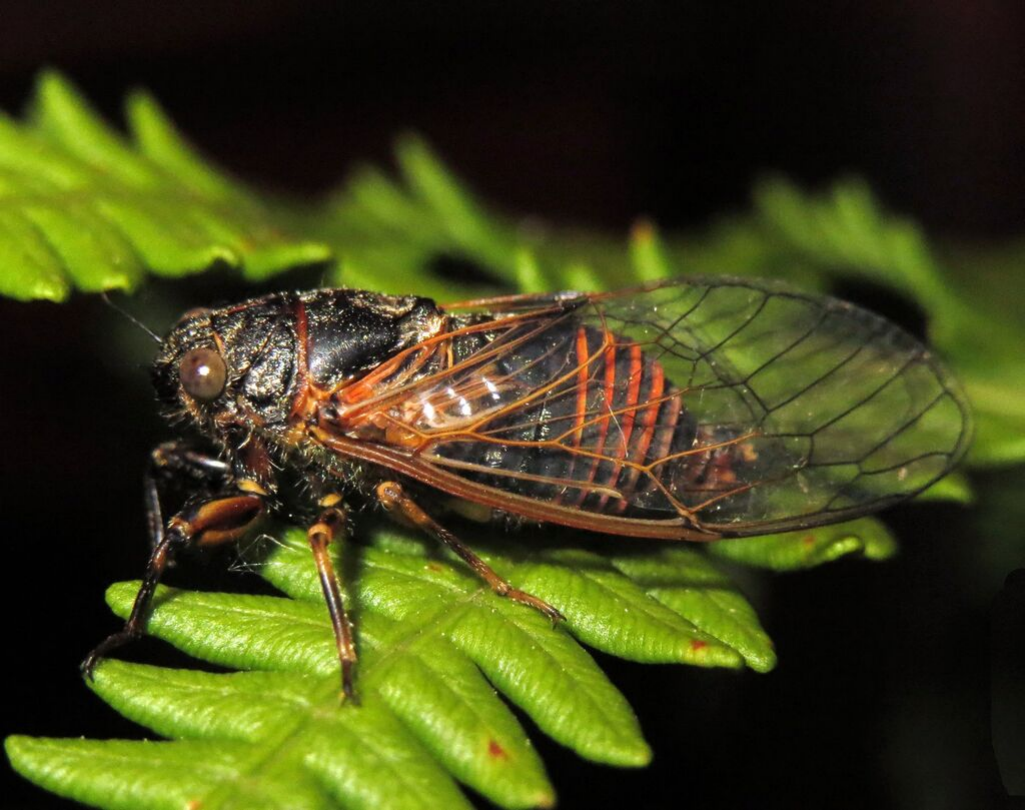
***Oligoglena tibialis* male.**

**Figure 56.**
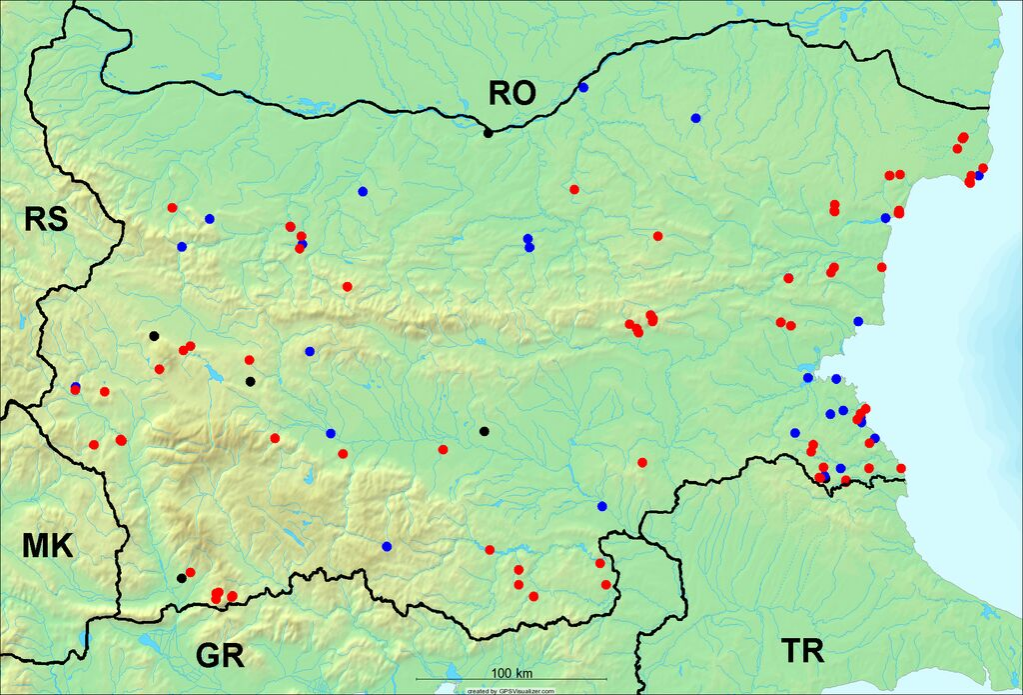
**Map with localities of *Oligoglena tibialis* in Bulgaria.** Black - literature data, blue - data from collections, red - bioacoustic data collected in this survey.

**Figure 57.**
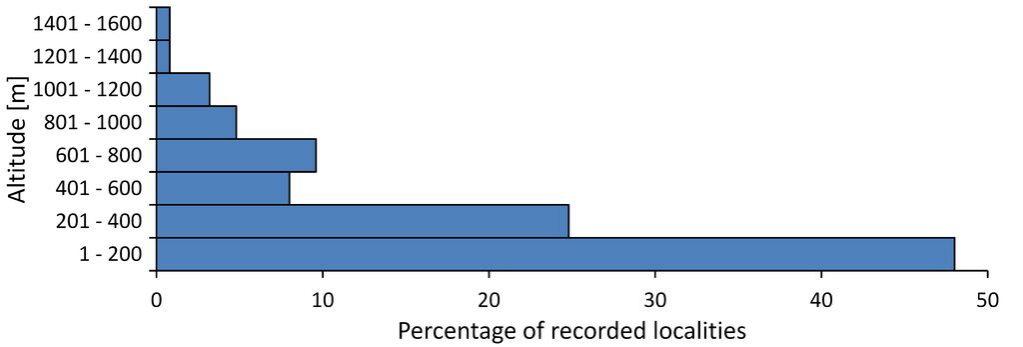
**Altitudinal distribution of *Oligoglena tibialis*.** The percentage of the recorded localities is displayed in 200-metre altitude zones.

**Figure 58.**
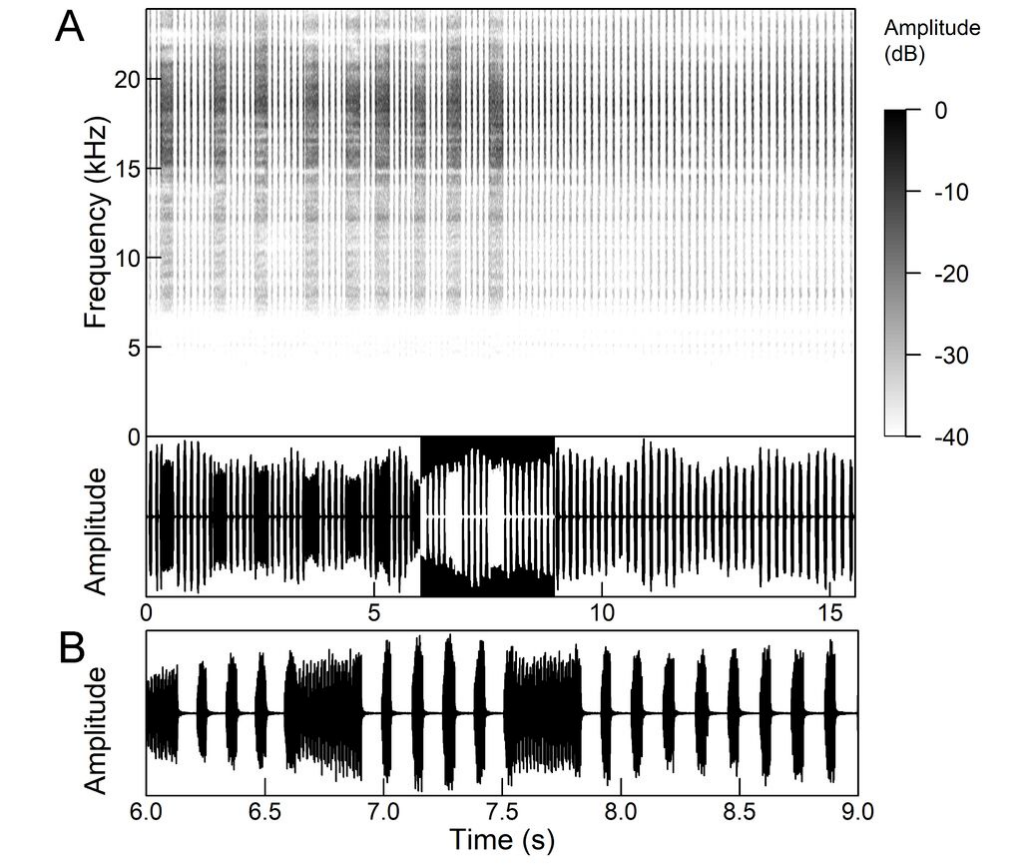
**Calling song of *Oligoglena tibialis*.** (A) spectrogram and oscillogram of the calling song, showing the transition from the first phrase to the second; (B) oscillogram of the enlarged part of the transition from the first phrase to the second one corresponding to the inverted window in (A).

**Figure 59.**
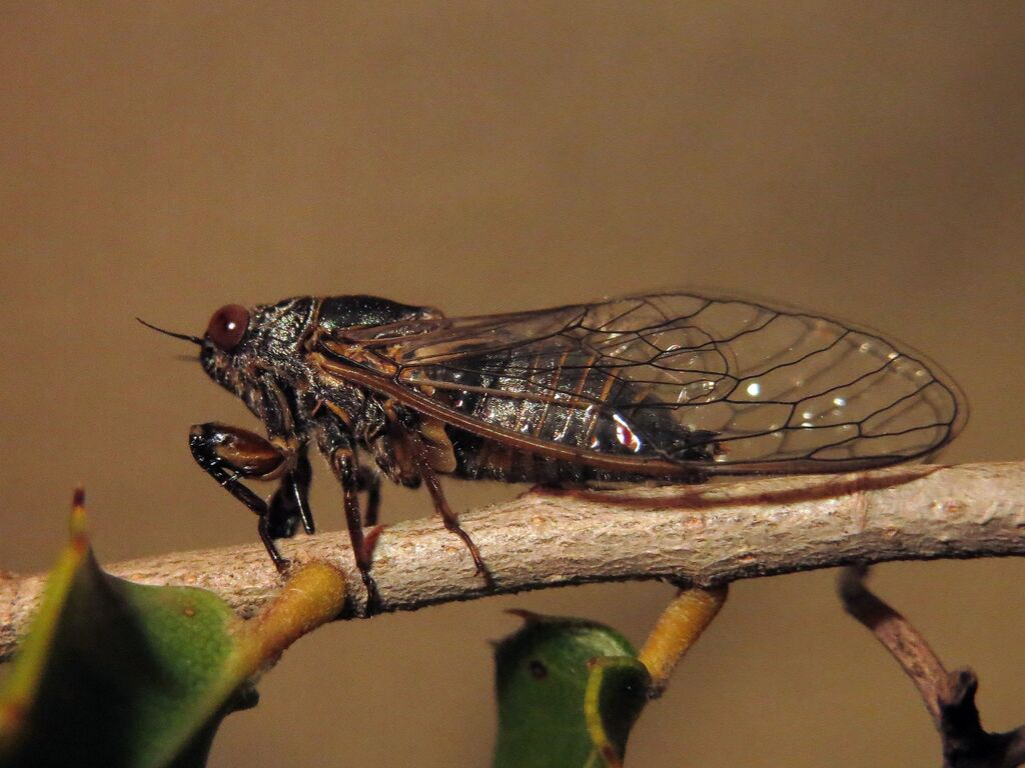
***Tettigettula pygmea* male.**

**Figure 60.**
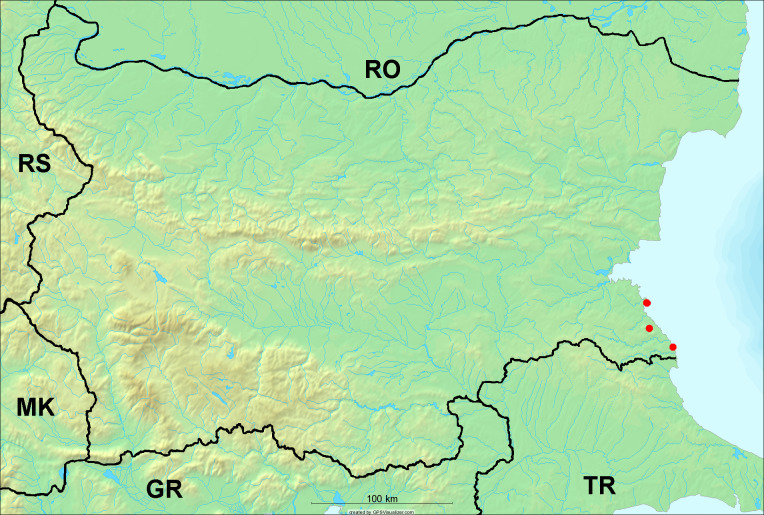
**Map with localities of *Tettigettula pygmea* in Bulgaria.** Red - bioacoustic data collected in this survey.

**Figure 61.**
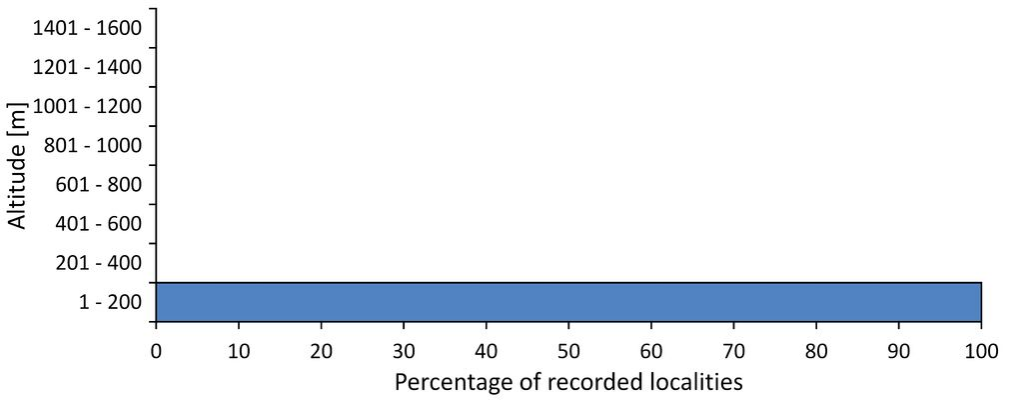
**Altitudinal distribution of *Tettigettula pygmea*.** The percentage of the recorded localities is displayed in 200-metre altitude zones.

**Figure 62.**
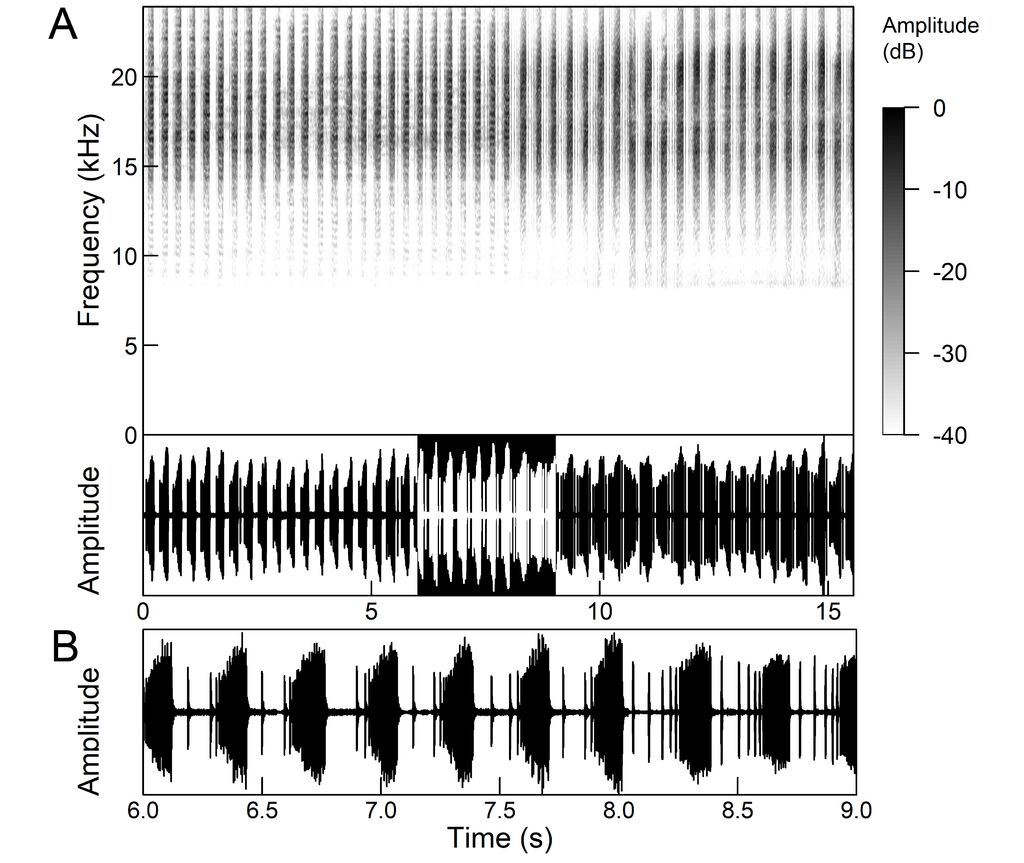
**First phrase of the calling song of *Tettigettulapygmea***. (A) spectrogram and oscillogram of the selected part of the first phrase of the calling song; (B) oscillogram of the enlarged part corresponding to the inverted window in (A).

**Figure 63.**
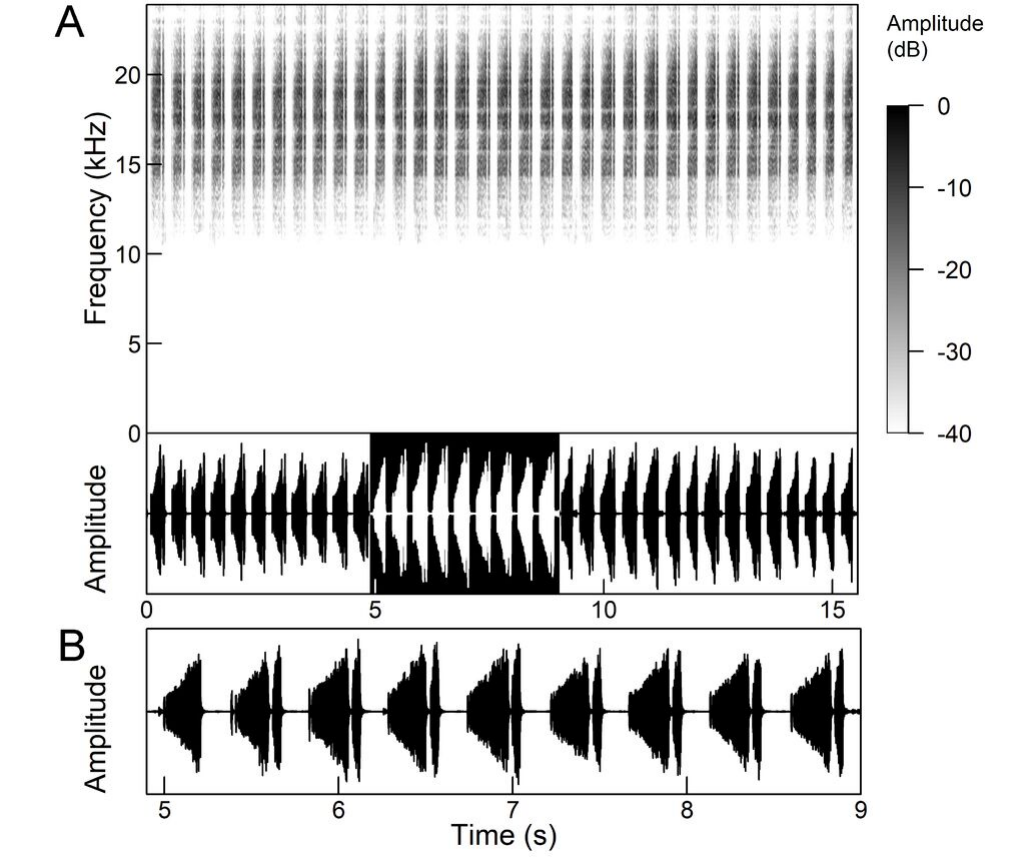
**Second phrase of the calling song of *Tettigettulapygmea***. (A) spectrogram and oscillogram of the selected part of the second phrase of the calling song; (B) oscillogram of the enlarged part corresponding to the inverted window in (A).

**Figure 64.**
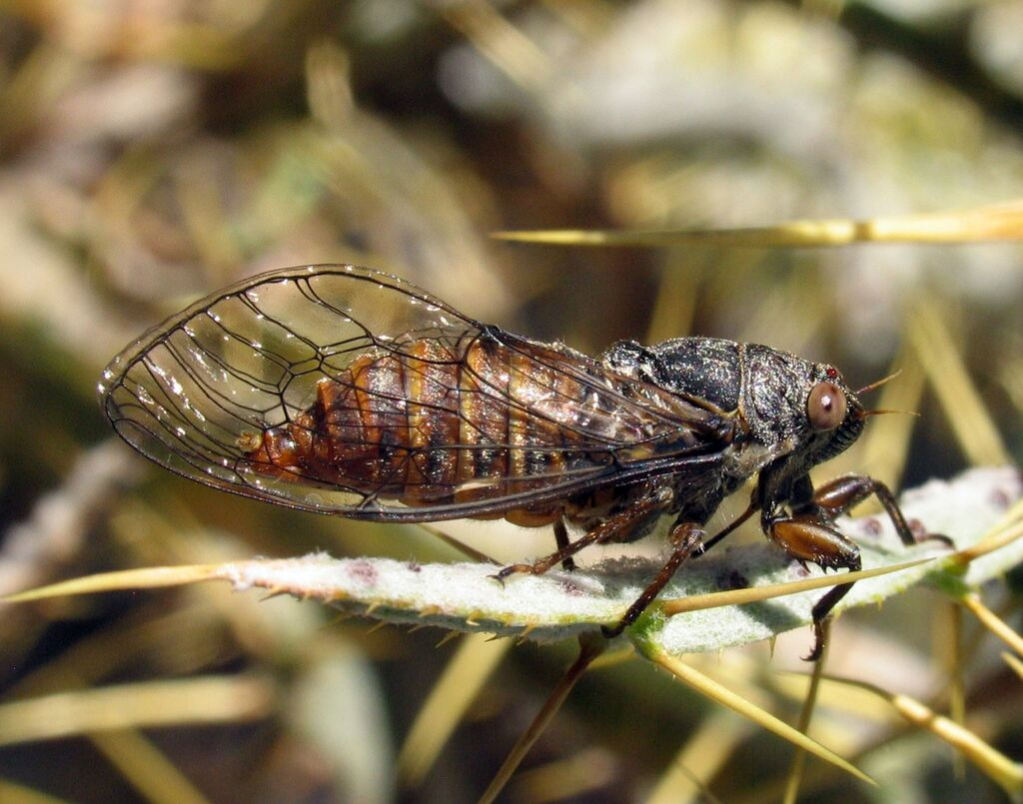
***Pagiphora annulata* male.**

**Figure 65.**
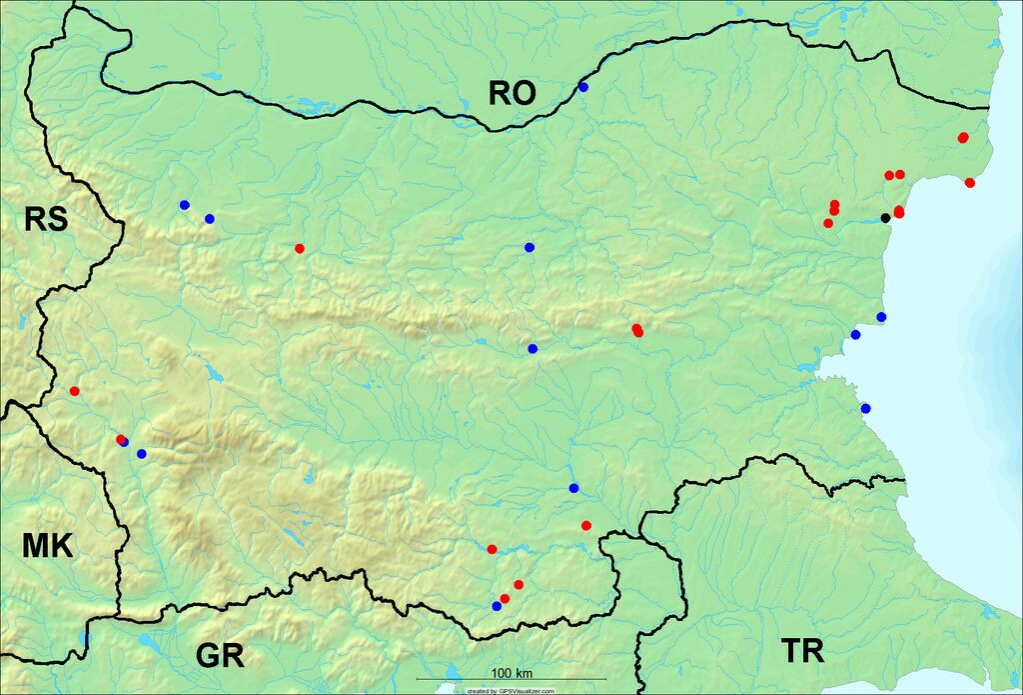
**Map with localities of *Pagiphora annulata* in Bulgaria.** Black - literature data, blue - data from collections, red - bioacoustic data collected in this survey.

**Figure 66.**
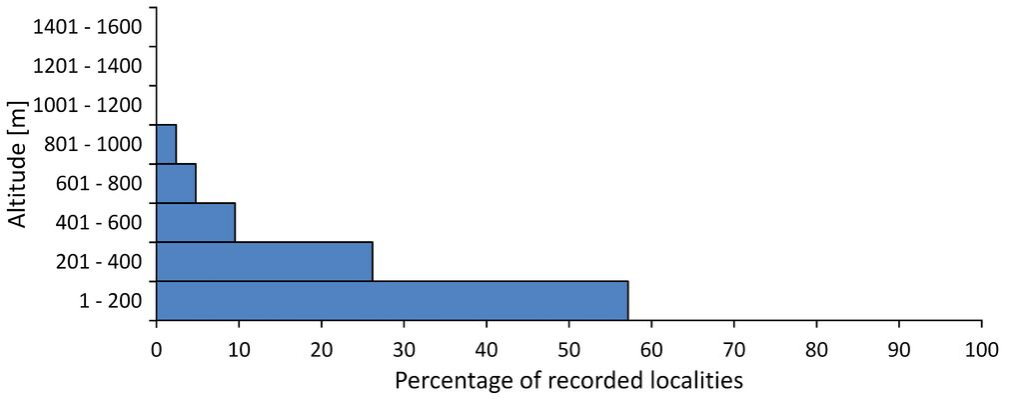
**Altitudinal distribution of *Pagiphora annulata*.** The percentage of the recorded localities is displayed in 200-metre altitude zones.

**Figure 67.**
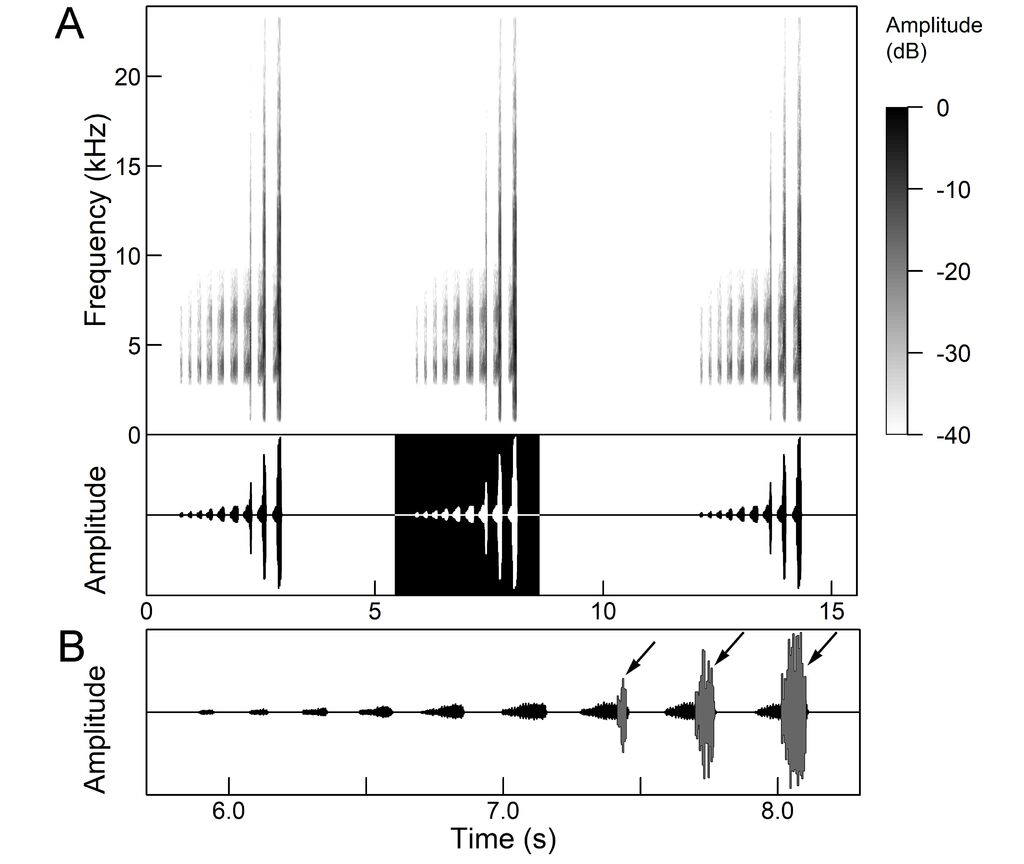
**Calling song of *Pagiphora annulata*.** (A) spectrogram and oscillogram of three phrases of the calling song; (B) oscillogram of one phrase corresponding to the inverted window in (A), black - tymbal echemes, grey and marked with arrows - wing flapping.

**Figure 68.**
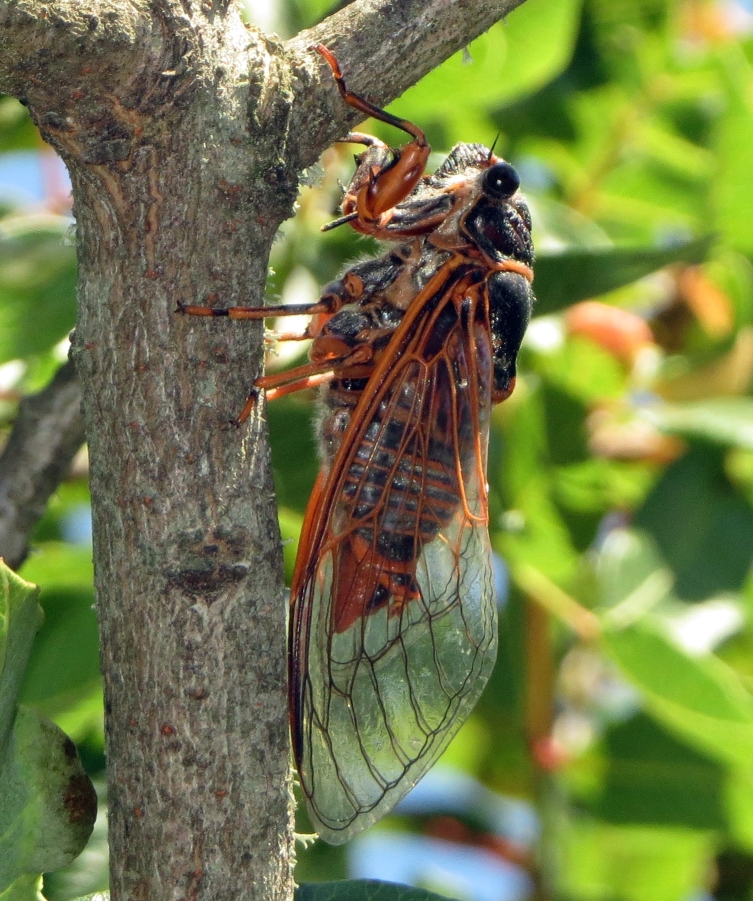
***Tibicina haematodes* male.**

**Figure 69.**
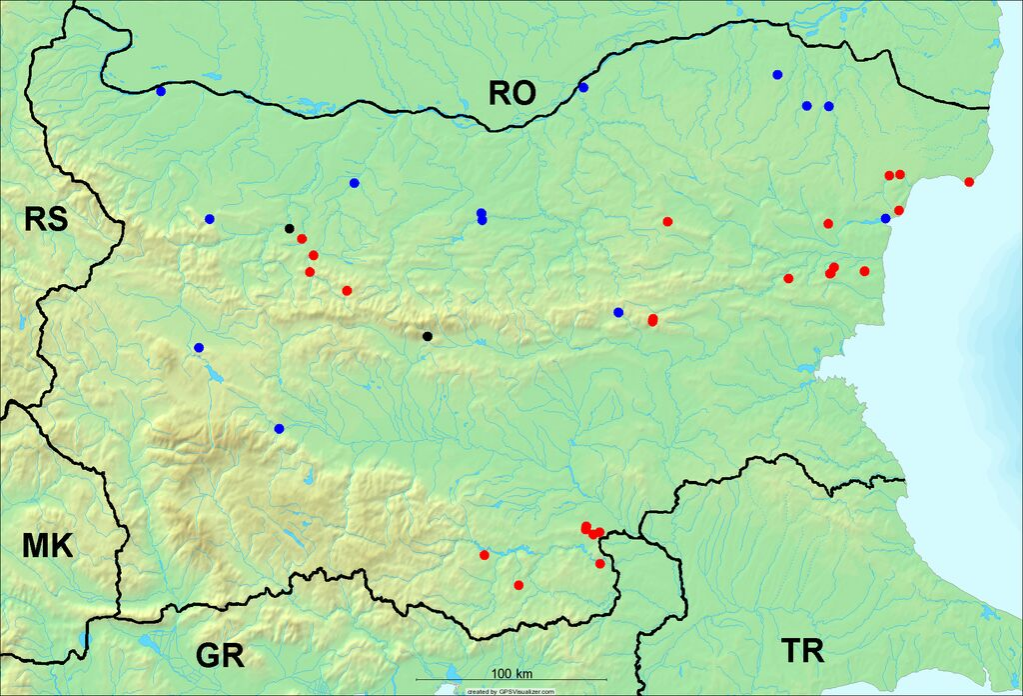
**Map with localities of *Tibicina haematodes* in Bulgaria.** Black - literature data, blue - data from collections, red - bioacoustic data collected in this survey.

**Figure 70.**
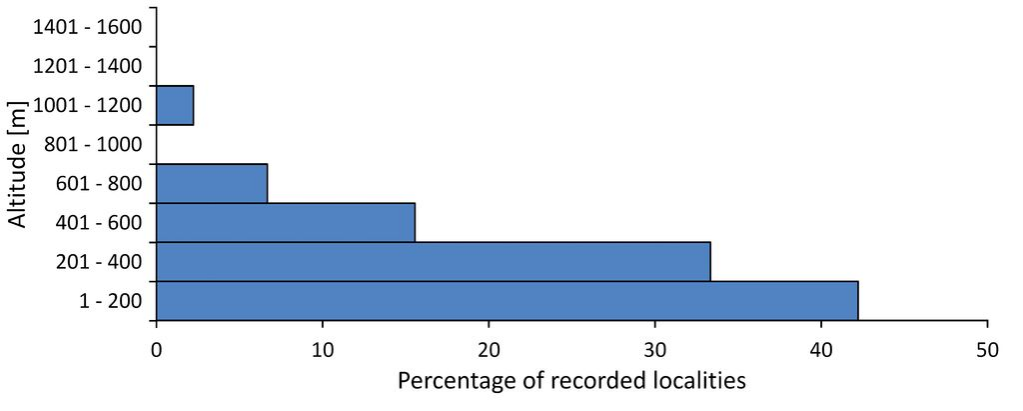
**Altitudinal distribution of *Tibicina haematodes*.** The percentage of the recorded localities is displayed in 200-metre altitude zones.

**Figure 71.**
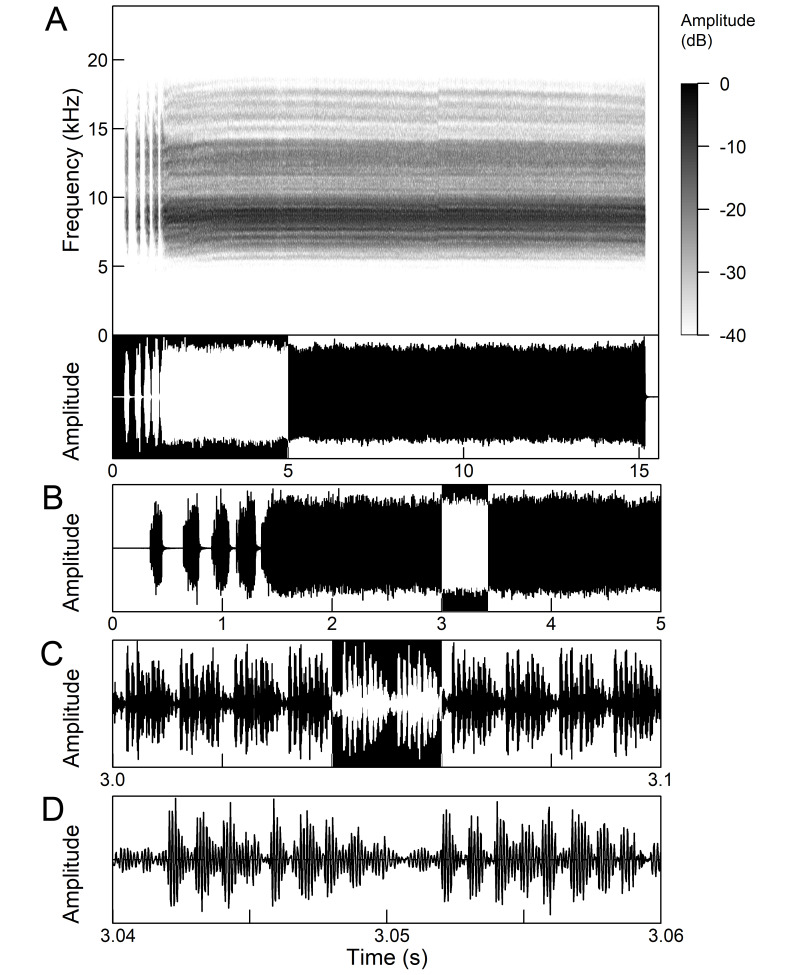
**Calling song of *Tibicina haematodes*.** (A) spectrogram and oscillogram of the calling song; (B) oscillogram of the beginning of the calling song with four introductory echemes corresponding to the inverse window in (A); (C) detailed oscillogram showing subgroups of pulses corresponding to the inverse window in (B); (D) detailed oscillogram showing pulses corresponding to the inverse window in (C).

**Figure 72.**
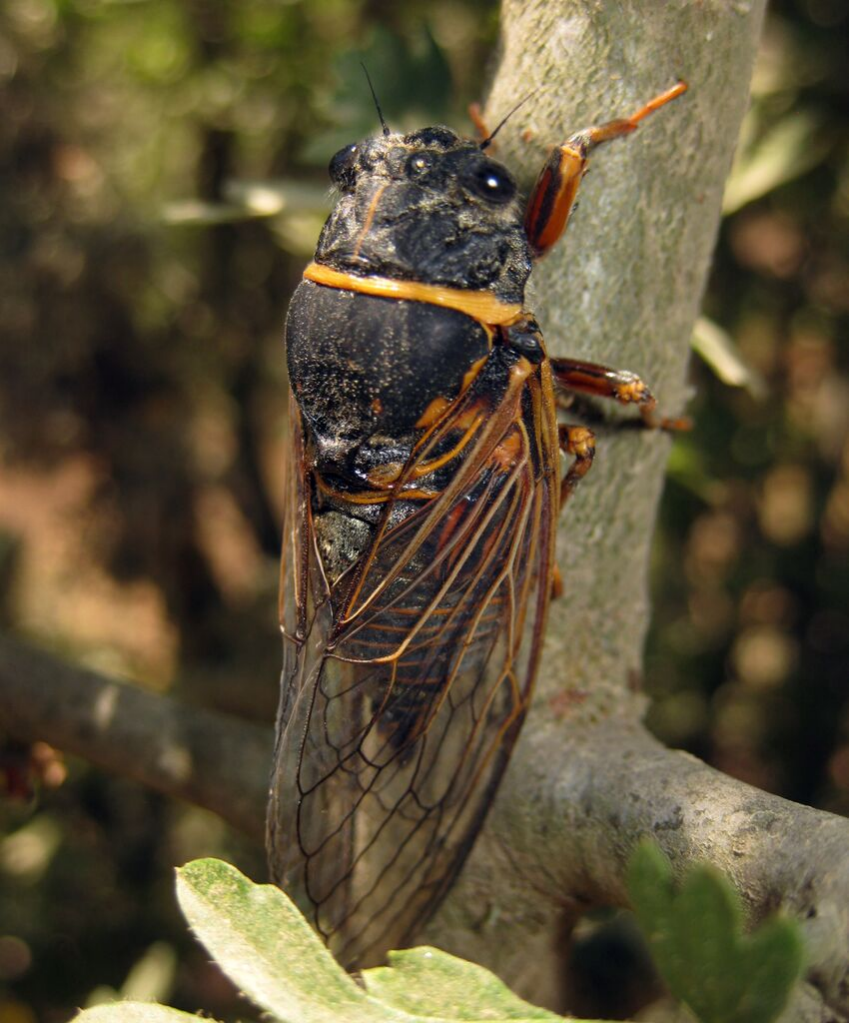
***Tibicina steveni* male.**

**Figure 73.**
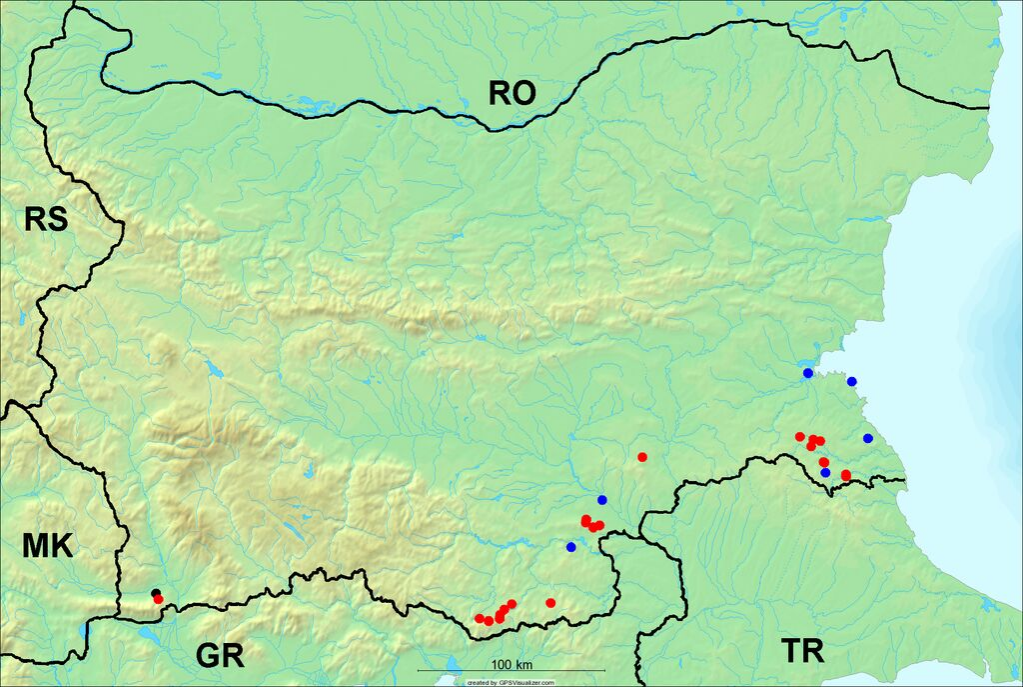
**Map with localities of *Tibicina steveni* in Bulgaria.** Black - literature data, blue - data from collections, red - bioacoustic data collected in this survey.

**Figure 74.**
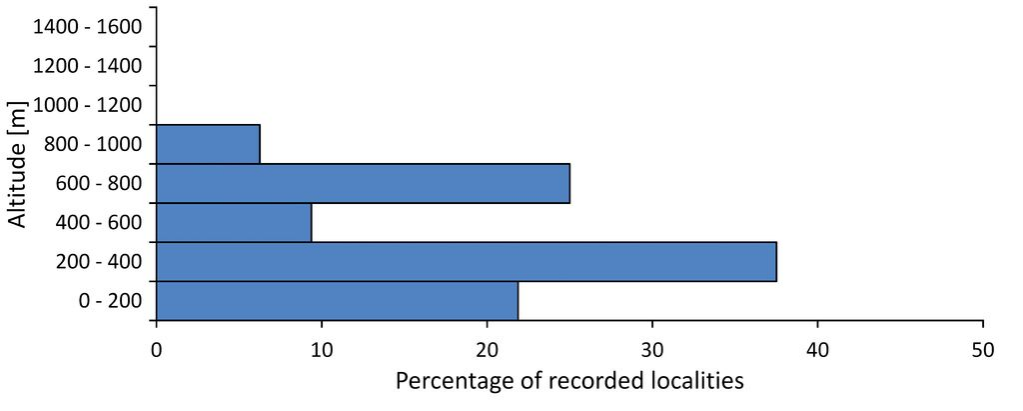
**Altitudinal distribution of *Tibicina steveni*.** The percentage of the recorded localities is displayed in 200-metre altitude zones.

**Figure 75.**
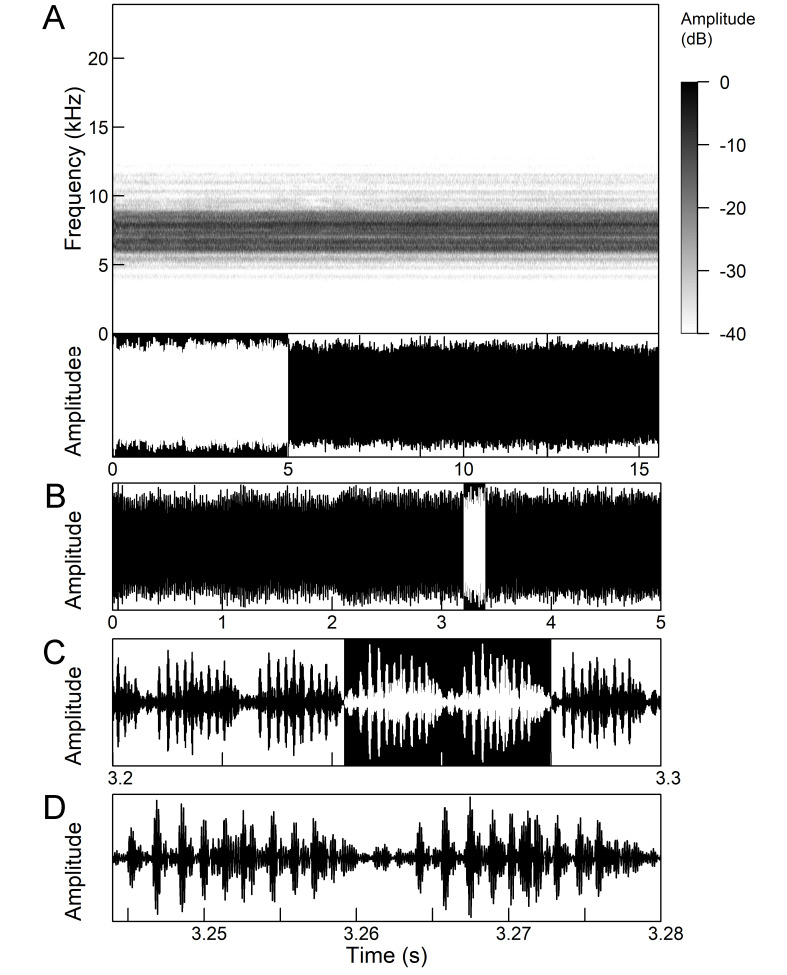
**Calling song of *Tibicina steveni*.** (A) spectrogram and oscillogram of the selected part of the calling song; (B) oscillogram of the enlarged part corresponding to the inverted window in (A); (C) detailed oscillogram showing subgroups of pulses corresponding to the inverted window in (B); (D) detailed oscillogram showing pulses corresponding to the inverted window in (C).

Table 1 summarises the number of provinces and localities where cicada species were found in Bulgaria. Surprisingly, the most widespread species is *Oligoglena tibialis* (Fig. 56), followed by *Dimissalna dimissa* (Fig. 51) and not one of the three largest cicada species, i.e. *Cicada orni* (Fig. 5), *Lyristes plebejus* (Fig. 9) or *Tibicina haematodes* (Fig. 69). Probably the knowledge about the distribution of *Cicada orni* (Fig. 5) is, at least, underestimated, because the lowland localities were not investigated proportionally to the localities at higher altitudes. The distribution of *Tibicina steveni* (Fig. 73) is limited to the mountains of southern Bulgaria and extends along the border with Greece. *Cicadatra hyalina* (Fig. 18), *Cicadatra platyptera* (Fig. 28), *Cicadetta cantilatrix* (Fig. 43) and *Tettigettula pygmea* (Fig. 60) are very rare in Bulgaria or have a very limited dustribution. *Cicadatra hyalina* (Fig. 18) and *Cicadatra platyptera* (Fig. 28) are limited by the availability of steppe habitats. In our opinion, suitable habitats for *Cicadetta cantilatrix* (Fig. 43) and *Tettigettula pygmea* (Fig. 60) are available in Bulgaria and further investigations are necessary, especially since both species are also distributed in neighbouring Greece (Gogala and Trilar, unpublished data), North Macedonia (Gogala et al. 2005) and Romania (Trilar et al. 2006a, Trilar and Gogala 2008).

**Figure 76.**
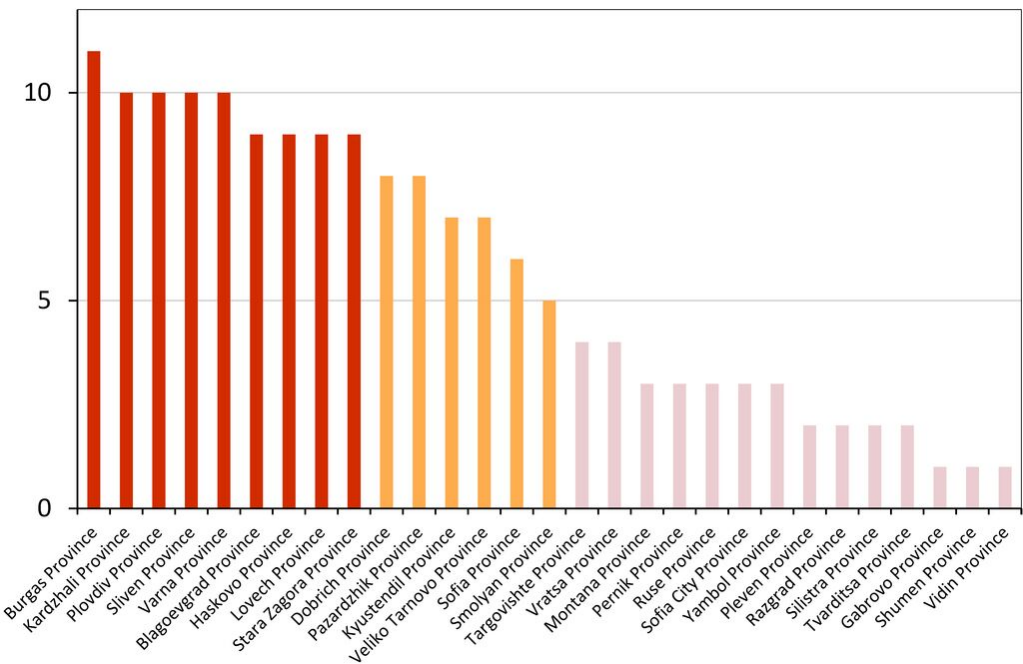
**Number of cicada species per province in Bulgaria.** Pink - 1-4 species per Province, orange - 5-8, red - 9-11.

**Figure 77.**
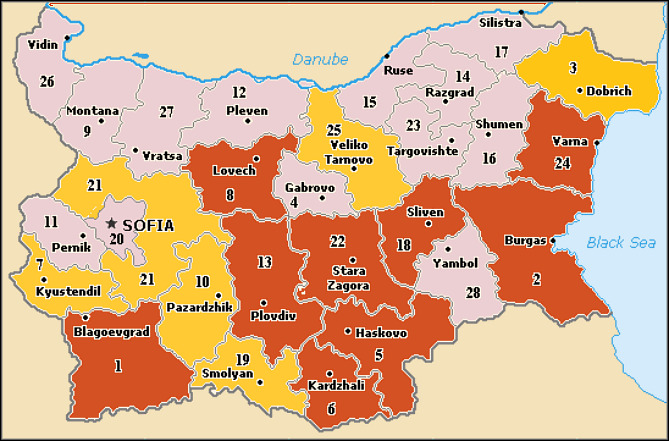
**Distribution of cicadas by province in Bulgaria.** Pink - 1-4 species per province, orange - 5-8, red - 9-11. Source of the map Pensionero 2011. 1 - Blagoevgrad Province, 2 - Burgas Province, 3 - Dobrich Province, 4 - Gabrovo Province, 5 - Haskovo Province, 6 - Kardzhali Province, 7 - Kyustendil Province, 8 - Lovech Province, 9 - Montana Province, 10 - Pazardzhik Province, 11 - Pernik Province, 12 - Pleven Province, 13 - Plovdiv Province, 14 - Razgrad Province, 15 - Ruse Province, 16 - Shumen Province, 17 - Silistra Province, 18 - Sliven Province, 19 - Smolyan Province, 20 - Sofia Province, 21 - Stara Zagora Province, 22 - Targovishte Province, 23 - Varna Province, 24 - Veliko Tarnovo Province, 25 - Vidin Province, 26 - Vratsa Province, 27 - Yambol Province

Cicadas are not evenly distributed in Bulgaria. Figs 76, 77 summarise the number of cicada species per province in Bulgaria. These results should be evaluated taking into account the fact that the same research efforts were not made in all provinces. The number of cicada species is lowest and probably underestimated in the pink-marked provinces (1-4 species per province) (Fig. 77), especially in Montana, Pernik, Ruse, Sofia City, Yambol, Tvarditsa, Gabrovo, Shumen and Vidin Provinces. During our fieldwork in these Provinces, we did not spend enough time in the lowlands while trying to reach higher places or we had bad weather conditions where the cicadas did not sing. There is probably not a single Province in Bulgaria where at least four lowland cicada species are present, namely *Cicada orni*, *Lyristes plebejus*, *Dimissalna dimissa* and *Oligoglena tibialis*.

The Provinces with a moderate number of species, which are marked orange (5-8 species per Province), have a lower altitudinal range and probably a lower habitat diversity. The Dobrich Province is a lowland area rich in steppe habitats and the mountain cicada species are missing. In contrast, Smolyan Province has mostly high mountains and the steppe cicada species are missing. Additionally, some areas in the Danube plain, such as Silistra, Razgrad and Pleven Province are lowland areas with a considerable amount of wetland habitats, which are not suitable for the development of the underground cicada larvae.

We found that the richest cicada biodiversity is found in areas with a greater diversity of relief, ranging from lowlands to low mountain ranges and high mountains. These areas are the Struma Valley with the Kresna Gorge and Pirin Mountain, Eastern Rhodopes, Balkan Mountains and Strandzha Mountain, in Figs 76, 77 marked in red (9-11 species per Province) and include Burgas, Kardzhali, Plovdiv, Sliven, Varna, Blagoevgrad, Haskovo, Lovech and Stara Zagora Provinces. In the lowlands, *Cicada orni*, *Lyristes plebejus*, *Cicadatra atra*, *Dimissalna dimissa* and *Oligoglena tibialis* are common everywhere and occasionally *Pagiphora annulata* and *Tibicina haematodes* are present. In the low mountain range, *Cicadetta macedonica* is everywhere and in some places also *Cicadetta cantilatrix* and *Cicadetta brevipennis* s. lat. and in the high mountains *Cicadetta montana* s. str.

In total, during this investigation, we discovered seven new species in the collections or in the field for the fauna of Bulgarian singing cicadas, namely *Cicadatra persica*, Cicadatra platyptera, *Cicadetta montana* s. str., *Cicadetta brevipennis* s. lat., *Cicadetta cantilatrix*, *Cicadetta macedonica* and *Tettigettula pygmea*. Amongst these species, *Cicadetta montana* s. str., *Cicadetta brevipennis* s. lat. and *Cicadetta macedonica* are relatively common and well distributed. All other species, including *Cicadatra hyalina* and *Cicadatra persica*, are rare and have a very limited distribution. In Bulgaria, we have data for *Cicadatra hyalina* from only four localities, *Cicadatra platyptera* from seven localities, *Cicadatra persica* from one locality, *Cicadetta cantilatrix* from four localities and for *Tettigettula pygmea* from three localities. This raises the question of the protection of these species and their habitats. There is also an urgent need for further research on the localities described in the above-mentioned literature and collections to confirm the recent occurrence of these rare species, to determine the state of their habitats and to propose conservation measures.

In literature, we also find the records for four additional species: *Cicadatra persica* (Háva 2016), *Cicadetta mediterranea* (Pelov 1968, Nast 1972, Nast 1987), *Tympanistalna gastrica* (Nedyalkov 1908, Nast 1972, Nast 1987) and *Saticula coriaria* (Yoakimov 1909), which we could not detect during fieldwork and these are discussed below.

The specimen of *Cicadatra persica* from Sozopol on the Black Sea coast cited by Háva (2016) is, in our opinion, a wrongly identified unusually coloured *Cicadatra atra*. We also found two male and one female specimens from Sliven in the collections of SOFM and MZPW. Using bioacoustic methods, we searched in Sliven and its surroundings under ideal weather conditions for singing cicadas and in suitable habitat (on the basis of our own field experience from North Macedonia (Gogala and Trilar 1998, Gogala and Trilar 2003, Gogala et al. 2005), but we could not detect any *Cicadatra persica*. As the habitat is in good condition and unaffected by human activities, further investigations are necessary to confirm the presence or absence of *Cicadatra persica* in Sliven and its surroundings.

*Cicadetta mediterranea* was collected (one female) by V. Pelov in August 1957 on dry open slopes north of the village of Podkova (41.398°N, 25.401°E; Kardzhali Province: Kirkovo Municipality) and published with the note that it was determined according to Melichar 1896 (Pelov 1968). Unfortunately, the collection was burnt in a fire in the laboratory (Pelov, personal communication) and the determination cannot be confirmed. In our opinion, it is a rather unusual habitat for this species, ecologically bound to a narrow coastal zone and to herbaceous plants in this habitat (Gogala and Popov 1997). Future research will solve this mystery.

*Tympanistalna gastrica* is distributed in central and southern Portugal (Sueur et al. 2004). The species was listed for northern and southern Bulgaria by Nedyalkov (1908) (under the name *Cicadetta gastrica* Stål) and later by Nast (1972), Nast (1987). In collections, we find *Pagiphora annulata* misidentified as *Tympanistalna gastrica* and we assume that this is also the case for Nedyalkov (1908).

*Saticula coriaria* is a north African species (Boulard 1981b), which was erroneously listed for Bulgaria by Yoakimov (1909). Yoakimov found the species, which he named *Cicadetta coriacea* Stål, in July 1907 near the lake Straldzhansko (Стралджанско езеро). We cannot speculate to which species Yoakimov was referring.

We propose to exclude both species, *Tympanistalna gastrica* (Stål 1854) and *Saticula coriaria* Stål 1866, from the list of Bulgarian cicadas.

We also solved the riddle with the literature quote from *Tibicina steveni* from Petrich, cited by Janković (1971). The citation was given as literature data, but without reference to the source and was erroneously placed in North Macedonia (Gogala et al. 2005). We recorded the singing males of this species on 23 July 2008, near Bozhkinoto on the slope of Belasitsa Mt. and confirm the presence of *Tibicina steveni* in this area.

Yoakimov (1909), Nast (1972) and Nast (1987) are also listed for the fauna of the Bulgarian singing cicadas *Cicadetta montana* (Scopoli 1772), which cannot be assigned to the species level, since a definitive proof of identity of the *Cicadetta montana* species complex is only possible with bioacoustic and/or molecular methods (i.e. Gogala and Trilar 2014, Wade et al. 2015). The same applies to most of the specimens of this complex, which are kept in BFUS, SOFM and ZISB collections. Recent bioacoustic studies have shown that *Cicadetta montana* (Scopoli 1772), once thought to be a single widespread Palaearctic cicada species, is, in fact, a complex of morphologically-similar sister species best characterised by their song patterns (i.e. Gogala and Trilar 2014, Sueur and Puissant 2007b, Hertach et al. 2016) and, to some extent, can also be separated by molecular methods (i.e. Wade et al. 2015). With the use of bioacoustic methods, we found four species from this complex in Bulgaria: *Cicadetta montana* s. str. (Scopoli 1772), *Cicadetta brevipennis* s. lat. Fieber 1876, *Cicadetta cantilatrix* Sueur & Puissant 2007 and *Cicadetta macedonica* Schedl 1999.

Using bioacoustic methods, we also discovered unknown song patterns similar to the songs of *Cicadetta montana* species complex, which could belong to three or four additional taxa. From a population with undescribed song patterns, we were also able to collect a reasonable number of specimens and the description of the species is in preparation. The second singing pattern shows cline changes in the time parameters from west to east with a step change at the middle of the geographical distribution, which could indicate two separate taxa. However, we could collect only four males from both distribution margins and a few females from one location, for which we are not convinced that they belong to the males recorded and collected. In the population of the third song pattern, we could not collect any specimens because the animals live high up in the treetops. Therefore, we need additional specimens and recordings to confirm this hypothesis.

The Bulgarian fauna of the singing cicada currently consists of 16 confirmed and 3-4 potential species. Twenty species from Bulgaria show the richness of the Mediterranean cicada fauna, which can be compared with the neighbouring countries. In Greece, 33 species (Gogala and Trilar, unpublished data) and 15 in North Macedonia (Gogala et al. 2005) and Romania (Trilar et al. 2006a, Trilar and Gogala 2008) have been recorded. The comparison with equally-well studied countries in Western and Central Europe shows the transition between the northern regions, which are home to only a few species (Germany: five species (Nickel et al. 2016), Austria: eight species (Schedl 2002, Schedl 2004, Trilar and Holzinger 2004), Switzerland: 10 species (Hertach and Nagel 2013) and Slovenia: 11 species (Gogala and Gogala 1999, Gogala and Trilar 2004)) and the southern regions, which are rich in cicada species (Portugal: 13 species (Sueur et al. 2004) and France: 20 species (Puissant 2006, Puissant and Sueur 2011)).

## Supplementary Material

F47A9B5A-CEB9-54F7-BA11-247F2C1ECBE6 10.3897/BDJ.8.e54424.suppl1 Supplementary material 1 *Cicada orni* faunistic data. Checklist and provisional atlas of singing cicadas (Hemiptera: Cicadidae) of Bulgaria, based on bioacoustics Data type Occurrences File: oo_449188.csv https://binary.pensoft.net/file/449188 Tomi Trilar, Ilia Gjonov, Matija Gogala

DA5560E5-C617-5D47-8A39-CBD84EEC3E6B 10.3897/BDJ.8.e54424.suppl2 Supplementary material 2 *Lyristes plebejus* faunistic data. Checklist and provisional atlas of singing cicadas (Hemiptera: Cicadidae) of Bulgaria, based on bioacoustics Data type Occurrences File: oo_449189.csv https://binary.pensoft.net/file/449189 Tomi Trilar, Ilia Gjonov, Matija Gogala

55FF76E8-AE39-59A0-8EDE-43B7EF590546 10.3897/BDJ.8.e54424.suppl3 Supplementary material 3 *Cicadatra atra* faunistic data. Checklist and provisional atlas of singing cicadas (Hemiptera: Cicadidae) of Bulgaria, based on bioacoustics Data type Occurrences File: oo_449191.csv https://binary.pensoft.net/file/449191 Tomi Trilar, Ilia Gjonov, Matija Gogala

3C336F89-D9D8-5468-AE1C-839195D52719 10.3897/BDJ.8.e54424.suppl4 Supplementary material 4 *Cicadatra hyalina* faunistic data. Checklist and provisional atlas of singing cicadas (Hemiptera: Cicadidae) of Bulgaria, based on bioacoustics Data type Occurrences File: oo_449193.csv https://binary.pensoft.net/file/449193 Tomi Trilar, Ilia Gjonov, Matija Gogala

38045A9B-FC8E-53DB-B306-97AEA19F08A8 10.3897/BDJ.8.e54424.suppl5 Supplementary material 5 *Cicadatra persica* faunistic data. Checklist and provisional atlas of singing cicadas (Hemiptera: Cicadidae) of Bulgaria, based on bioacoustics Data type Occurrences File: oo_449194.csv https://binary.pensoft.net/file/449194 Tomi Trilar, Ilia Gjonov, Matija Gogala

512078C9-255C-555A-A7A2-8D83EDBA3E75 10.3897/BDJ.8.e54424.suppl6 Supplementary material 6 *Cicadatra platyptera* faunistic data. Checklist and provisional atlas of singing cicadas (Hemiptera: Cicadidae) of Bulgaria, based on bioacoustics Data type Occurrences File: oo_449196.csv https://binary.pensoft.net/file/449196 Tomi Trilar, Ilia Gjonov, Matija Gogala

881CB1F8-1C13-5C22-BB4E-BDD1998D6671 10.3897/BDJ.8.e54424.suppl7 Supplementary material 7 *Cicadetta montana* s. lat. faunistic data. Checklist and provisional atlas of singing cicadas (Hemiptera: Cicadidae) of Bulgaria, based on bioacoustics Data type Occurrences File: oo_449205.csv https://binary.pensoft.net/file/449205 Tomi Trilar, Ilia Gjonov, Matija Gogala

8C7E91E4-9427-5A06-9ABC-48966D32CAE9 10.3897/BDJ.8.e54424.suppl8 Supplementary material 8 *Cicadetta montana* s. str. faunistic data. Checklist and provisional atlas of singing cicadas (Hemiptera: Cicadidae) of Bulgaria, based on bioacoustics Data type Occurrences File: oo_449204.csv https://binary.pensoft.net/file/449204 Tomi Trilar, Ilia Gjonov, Matija Gogala

77E4921C-C8C5-57C0-BE24-88508E0B27C9 10.3897/BDJ.8.e54424.suppl9 Supplementary material 9 *Cicadetta brevipennis* s. lat. faunistic data. Checklist and provisional atlas of singing cicadas (Hemiptera: Cicadidae) of Bulgaria, based on bioacoustics Data type Occurences File: oo_449210.csv https://binary.pensoft.net/file/449210 Tomi Trilar, Ilia Gjonov, Matija Gogala

25181905-72C3-5309-8BC5-3649F6E6DE57 10.3897/BDJ.8.e54424.suppl10 Supplementary material 10 *Cicadetta cantilatrix* faunistic data. Checklist and provisional atlas of singing cicadas (Hemiptera: Cicadidae) of Bulgaria, based on bioacoustics Data type Occurrences File: oo_449207.csv https://binary.pensoft.net/file/449207 Tomi Trilar, Ilia Gjonov, Matija Gogala

B4213826-39B6-543A-B20A-46A3FD76C4B1 10.3897/BDJ.8.e54424.suppl11 Supplementary material 11 *Cicadetta macedonica* faunistic data. Checklist and provisional atlas of singing cicadas (Hemiptera: Cicadidae) of Bulgaria, based on bioacoustics Data type Occurrences File: oo_449211.csv https://binary.pensoft.net/file/449211 Tomi Trilar, Ilia Gjonov, Matija Gogala

8AC988E4-0DC1-5001-A450-BD8412657FDB 10.3897/BDJ.8.e54424.suppl12 Supplementary material 12 *Dimissalna dimissa* faunistic data. Checklist and provisional atlas of singing cicadas (Hemiptera: Cicadidae) of Bulgaria, based on bioacoustics Data type Occurrences File: oo_449212.csv https://binary.pensoft.net/file/449212 Tomi Trilar, Ilia Gjonov, Matija Gogala

818B379C-1303-5794-B526-B8E6014830E0 10.3897/BDJ.8.e54424.suppl13 Supplementary material 13 *Oligoglena tibialis* faunistic data. Checklist and provisional atlas of singing cicadas (Hemiptera: Cicadidae) of Bulgaria, based on bioacoustics Data type Occurences File: oo_449233.csv https://binary.pensoft.net/file/449233 Tomi Trilar, Ilia Gjonov, Matija Gogala

1323D3B4-0D96-589D-8452-23AE7AFFEC1B 10.3897/BDJ.8.e54424.suppl14 Supplementary material 14 *Tettigettula pygmea* faunistic data. Checklist and provisional atlas of singing cicadas (Hemiptera: Cicadidae) of Bulgaria, based on bioacoustics Data type Occurrences File: oo_449215.csv https://binary.pensoft.net/file/449215 Tomi Trilar, Ilia Gjonov, Matija Gogala

E547249D-E28A-5882-BA21-04367AEE4DB1 10.3897/BDJ.8.e54424.suppl15 Supplementary material 15 *Pagiphora annulata* faunistic data. Checklist and provisional atlas of singing cicadas (Hemiptera: Cicadidae) of Bulgaria, based on bioacoustics Data type Occurrences File: oo_449218.csv https://binary.pensoft.net/file/449218 Tomi Trilar, Ilia Gjonov, Matija Gogala

544E6EBD-83D3-5951-AEDA-FB0134C35835 10.3897/BDJ.8.e54424.suppl16 Supplementary material 16 *Tibicina haematodes* faunistic data. Checklist and provisional atlas of singing cicadas (Hemiptera: Cicadidae) of Bulgaria, based on bioacoustics Data type Occurrences File: oo_449220.csv https://binary.pensoft.net/file/449220 Tomi Trilar, Ilia Gjonov, Matija Gogala

43CE967F-F6EB-5A8A-9589-F9B02C81B50F 10.3897/BDJ.8.e54424.suppl17 Supplementary material 17 *Tibicina steveni* faunistic data. Checklist and provisional atlas of singing cicadas (Hemiptera: Cicadidae) of Bulgaria, based on bioacoustics Data type Occurrences File: oo_449222.csv https://binary.pensoft.net/file/449222 Tomi Trilar, Ilia Gjonov, Matija Gogala

XML Treatment for Cicadinae

XML Treatment for Cicadini

XML Treatment for Cicada orni

XML Treatment for Cryptotympanini

XML Treatment for Lyristes plebejus

XML Treatment for Cicadettinae

XML Treatment for Cicadatrini

XML Treatment for Cicadatra atra

XML Treatment for Cicadatra hyalina

XML Treatment for Cicadatra persica

XML Treatment for Cicadatra platyptera

XML Treatment for Cicadettini

XML Treatment for Cicadetta montana

XML Treatment for Cicadetta montana

XML Treatment for Cicadetta brevipennis

XML Treatment for Cicadetta cantilatrix

XML Treatment for Cicadetta macedonica

XML Treatment for Dimissalna dimissa

XML Treatment for Oligoglena tibialis

XML Treatment for Tettigettula pygmea

XML Treatment for Pagiphorini

XML Treatment for Pagiphora annulata

XML Treatment for Tibicininae

XML Treatment for Tibicinini

XML Treatment for Tibicina haematodes

XML Treatment for Tibicina steveni

## Figures and Tables

**Table 1.** **Number of provinces and localities where cicada species were found in Bulgaria**

**Species**	**No of provinces**	**No of localities**
*Oligoglena tibialis*	23	125
*Dimissalna dimissa*	19	122
*Lyristes plebejus*	17	76
*Tibicina haematodes*	17	45
*Cicadetta macedonica*	16	50
*Cicada orni*	15	74
*Cicadatra atra*	14	79
*Pagiphora annulata*	12	45
*Cicadetta montana* s. str.	9	40
*Cicadetta brevipennis* s. lat.	7	33
*Tibicina steveni*	4	32
*Cicadatra hyalina*	4	4
*Cicadatra platyptera*	3	7
*Cicadetta cantilatrix*	3	4
*Tettigettula pygmea*	1	3
*Cicadatra persica*	1	1
